# Aglatimagene besadenovec (CAN-2409) with radiotherapy for patients with localised prostate cancer: a phase 3, multicentre, randomised, double-blind, placebo-controlled trial

**DOI:** 10.1016/S1470-2045(26)00071-9

**Published:** 2026-06

**Authors:** Theodore L DeWeese, Andrea Manzanera, John Sylvester, Thomas Wheeler, Thomas Schroeder, Glen Gejerman, Gregory Chesnut, Thomas M Facelle, Mark G Garzotto, Christopher Pieczonka, Nilay M Gandhi, Steven Sukin, Michael A Liss, Ronald Tutrone, Bryan Mehlhaff, Stephen J Savage, Megan Goody, Jenessa Vogt, Shangbang Rao, Maria Lucia Silva Polanco, Francesca Barone, W Garrett Nichols, Paul P Tak

**Affiliations:** Departments of Radiation Oncology, Urology, and Oncology, Johns Hopkins School of Medicine, Baltimore MD, USA; Candel Therapeutics, Needham, MA, USA; Atlantic Urology Clinics, Myrtle Beach, SC, USA; Department of Pathology & Immunology, Baylor College of Medicine, Houston, TX, USA; University of New Mexico, Albuquerque, NM, USA; New Jersey Urology, Summit Health, Saddlebrook, NJ, USA; Walter Reed National Military Medical Center, Bethesda, MD, USA; Colorado Clinical Research, Golden, CO, USA; VA Portland Health Care System/OHSU, Portland, OR, USA; Associated Medical Professionals of NY,US Urology Partners, Syracuse, NY, USA; Potomac Urology Center, Alexandria, VA, USA; Texas Urology Specialists, Tomball, TX, USA; University of California San Diego, San Diego, CA, USA; Chesapeake Urology Research Associates, Towson, MD, USA; Oregon Urology Institute, Springfield, OR, USA; Ralph H Johnson VA Medical Center, Charleston, SC, USA; Candel Therapeutics, Needham, MA, USA; Candel Therapeutics, Needham, MA, USA; Candel Therapeutics, Needham, MA, USA; Candel Therapeutics, Needham, MA, USA; Candel Therapeutics, Needham, MA, USA; Candel Therapeutics, Needham, MA, USA; Candel Therapeutics, Needham, MA, USA; Faculdade de Medicina, Universidade de Lisboa, Portugal

## Abstract

**Background:**

About 30% of men with localised prostate cancer undergoing radiotherapy with curative intent have disease recurrence associated with progression-related symptoms and substantial toxicity of salvage therapies. Previous studies with aglatimagene besadenovec (CAN-2409, hereafter referred to as aglatimagene) showed synergy with radiation and immune-mediated cytotoxicity in patients with prostate cancer. We aimed to assess whether addition of aglatimagene plus prodrug (valacyclovir) to standard-of-care external beam radiation therapy (EBRT) could improve disease-free survival in this population.

**Methods:**

We conducted a phase 3, randomised, double-blind, placebo-controlled trial at 51 medical centres (26 community and 25 institutional or military) across the USA and Puerto Rico in patients with intermediate or high-risk prostate cancer. Patients aged at least 18 years who were planning to undergo EBRT and with an Eastern Cooperative Oncology Group score of 0–2 were eligible. Patients were randomly assigned (2:1) via central block-randomisation to receive either three courses of intraprostatic aglatimagene (5 × 10^11^ viral particles) plus valacyclovir or placebo plus valacyclovir, with randomisation stratified by risk category and androgen deprivation therapy (ADT) use. Patients received standard-of-care EBRT (78 Gy in 2 Gy fractions) or hypofractionated EBRT (60 Gy in 3 Gy fractions or 70 Gy in 2·5 Gy fractions) with optional ADT. The primary endpoint was disease-free survival, defined as time-from-randomisation to prostate cancer recurrence or death in the intent-to-treat population (all randomly assigned patients). Safety was assessed in all individuals who received at least one injection. The trial is registered at ClinicalTrials.gov, NCT01436968, and long-term follow-up is ongoing.

**Findings:**

Between Feb 21, 2012, and Sept 9, 2021, 745 men (591 [79%] White, 121 [16%] Black) were randomly assigned to receive aglatimagene plus valacyclovir (n=496) or placebo plus valacyclovir (n=249). After a median follow-up of 50·3 months (IQR 35·2–63·3), median disease-free survival was not reached (95% CI 121·78 to not reached) in the aglatimagene plus valacyclovir group versus 86·1 (IQR 29·7–143·0) months in the placebo plus valacyclovir group (hazard ratio 0·70, 95% CI 0·52–0·94; p=0·016). Treatment-emergent adverse events (TEAEs) of grade 3 or worse occurred in 40 (8%) of 479 patients in the aglatimagene group and 17 (7%)of 232 patients in the placebo group. The most common TEAE of grade 3 or worse was acute kidney injury in both the aglatimagene group (nine [2%] of 479 patients) and the placebo group (four [2%] of 232 patients). Serious adverse events occurred in 28 (6%) of 479 patients in the aglatimagene group and 17 (7%) of 232 in the placebo group. Treatment-related serious adverse events occurred in eight (2%) patients in the aglatimagene group (four acute kidney injury, two pyrexia, and one each influenza-like symptoms and urinary retention) and five (2%) in the placebo group (four acute kidney injury, and one each increased creatinine levels and skin rash; one patient reported two serious adverse events). No treatment-related deaths were reported.

**Interpretation:**

Aglatimagene plus valacyclovir was associated with longer disease-free survival than placebo plus valacyclovir when added to standard of radiotherapy for the treatment of localised prostate cancer, offering a meaningful benefit without increasing clinically significant toxicity.

**Funding:**

Candel Therapeutics and US National Institutes of Health.

## Introduction

Prostate cancer is a global health concern, with approximately 1·4 million new cases in 2020.^1^ Standard curative intent treatments for patients with intermediate-to-high-risk localised prostate cancer include radical prostatectomy or radiotherapy, combined with androgen deprivation therapy (ADT) when indicated.^2^ The treatment goal for these patients with clinically significant, localised prostate cancer is the prevention of cancer recurrence while minimising treatment-related side-effects. In patients managed with initial radiotherapy, local control is crucial because of the known increased risk of locoregional disease progression, metastatic disease, and prostate cancer-related mortality after prolonged follow-up in patients with positive prostate biopsies at least 2 years after radical therapy.^3^ In addition, after tumour recurrence, salvage therapies including long-term ADT are associated with side-effects and impaired quality of life (QOL).^4^ There is still a significant unmet need, as around 30% of patients will develop recurrent disease despite radical prostatectomy or radiotherapy.^5,6^

Aglatimagene besadenovec (aglatimagene or CAN-2409) is a replication-defective adenoviral vector carrying the herpes simplex virus thymidine kinase gene (*HSV-tk*). When injected into the prostate and used in combination with a prodrug (eg, valacyclovir), *HSV-tk* phosphorylates the prodrug into a cytotoxic nucleotide analogue that disrupts DNA replication in cells actively proliferating or undergoing DNA repair. Disrupting DNA repair induces tumour cell death, which leads to release of tumour antigens in the context of an inflamed tumour microenvironment, resulting in immunisation against the patient’s own variety of tumour antigens—primarily mediated by CD8^+^ T cells—and long-term tumour control, as previously shown in animal models of cancer.^7^ Consistent with these preclinical experiments, phase 1b clinical trials of aglatimagene plus prodrug in patients with localised prostate cancer showed that aglatimagene induces tumour cell necrosis and infiltration by CD8^+^ T cells in the prostate, which was confirmed in a larger study.^8–10^ These findings were replicated in phase 1b clinical trials of aglatimagene plus prodrug in pancreatic cancer, non-small-cell lung cancer, and glioblastoma.^11–13^

An open-label, phase 2a clinical trial of aglatimagene plus prodrug combined with radiotherapy in patients with low to high-risk localised prostate cancer showed freedom-from-failure rates of 91–94%, outperforming standard-of-care radiotherapy in concurrent studies with freedom-from-failure rates of 33–88%.^10,14,15^ In that trial, pathological complete response was observed in 94% of the available 2-year biopsies in patients who received aglatimagene plus prodrug combined with standard-of-care, compared with 37–73% in published populations who received standard of care alone.^14,16,17^ The beneficial effect of this combination was consistent with preclinical findings in a prostate cancer mouse model that showed synergy between radiotherapy and aglatimagene plus prodrug.^18^

Together, these studies provided the rationale for this phase 3, randomised, placebo-controlled clinical trial of aglatimagene plus valacyclovir in patients with intermediate to high-risk, localised prostate cancer who received standard-of-care external-beam radiotherapy (EBRT) with optional short-course ADT, treated with curative intent. Because our treatment goal was novel compared with those in previous trials in patients with more advanced disease, namely achieving disease-free survival in an increased proportion of the patients who aimed for cure, we sought agreement on the primary endpoint with the US Food and Drug Administration (FDA) before the start of the trial; therefore, the study was conducted under a Special Protocol Assessment agreed with the FDA. We aimed to assess whether, when added to standard radiotherapy, aglatimagene plus valacyclovir significantly reduced the risk of prostate cancer recurrence compared with placebo without adding significant toxicity, which would be expected to improve disease outcomes and reduce the need for salvage therapies.^3^

## Methods

### Study design and participants

This was a phase 3, multicentre, randomised, double-blind, placebo-controlled trial in patients with intermediate-risk to high-risk, localised prostate cancer who elected to undergo radiotherapy with curative intent. The study was completed at 51 medical centres (26 community and 25 institutional or military centres) across the USA and Puerto Rico, selected for their clinical expertise, patient population, trial experience, capacity to conduct the study in accordance with the protocol, and Good Clinical Practice. The trial followed the principles of Good Clinical Practice, with approval by the institutional review boards of all participating sites that included review of study design, safety, and risks. The trial was approved by a central institutional review board (BRANY IRB; approval number 2010–166), with additional approvals from local institutional review boards at participating sites as required. All patients provided written informed consent. There was no patient or public involvement in the study design, conduct, and reporting of this trial. The protocol is available in the appendix.

Eligible men were aged at least 18 years with newly diagnosed, histologically confirmed prostate adenocarcinoma either meeting National Comprehensive Cancer Network (NCCN) intermediate-risk criteria (prostate-specific antigen [PSA] 10–20 ng/mL, Gleason score 7, and tumour stage T2b–T2c) or with one high-risk criterion (PSA >20 ng/mL, Gleason score 8–10, or tumour stage T3a).^19^ Eligible patients had Eastern Cooperative Oncology Group performance scores of 0–2 and were planning to undergo prostate-only EBRT.^20^ Laboratory criteria included serum aspartate amino transferase (AST) of more than three-times the upper limit of normal, serum creatinine lower than 2 mg/dL, creatinine clearance below 30 mL/min, white blood count greater than 3000 per μL, and platelets greater than 100 000 per μL. Exclusion criteria included NCCN low-risk or more than one high-risk criteria, liver disease, HIV, immunosuppressant use, regional lymph node involvement, distant metastases, previous prostate cancer treatment, or planned whole-pelvic irradiation or orchiectomy. Risk stratification was based on standard-of-care assessments that included CT or MRI (pelvis and abdomen). All patients meeting criteria for high-risk disease were required to have a bone scan and pelvic CT or MRI before enrolment. Patient race and ethnicity were self-identified at enrolment on the basis of US federal data standards.^21^

This trial is registered at ClinicalTrials.gov, NCT01436968, and long-term follow-up is ongoing.

### Randomisation and masking

Patients were randomly assigned (2:1) to receive either aglatimagene plus valacyclovir or placebo plus valacyclovir. All patients received standard-of-care EBRT with optional short-course ADT. A central, computer-generated, block-randomisation method stratified by NCCN risk category and ADT use was used to generate treatment group assignment. The system used five randomly selected block sizes to maintain allocation concealment and reduce predictability. All patients, clinical and study staff, and study sponsor were masked to the treatment allocation. Randomisation treatment allocation occured centrally. Active treatment and placebo were identical in appearance, packaging, and treatment schedule. Allocation is being concealed until final database lock except in the event of a medical emergency requiring unmasking.

### Procedures

Patients received three courses of aglatimagene or placebo via ultrasound-guided intraprostatic-injections in outpatient clinics at least 14 days apart (2–8 weeks and 0–3 days pre-EBRT, and 2–3 weeks post-second injection of aglatimagene or placebo). Each vial of aglatimagene contained 5 × 10^11^ adenoviral vector particles in 2 mL buffer; placebo contained 2 mL buffer without vector. Each dose was administered in four 0·5 mL injections into the four quadrants of the prostate (appendix p 7). Valacyclovir (2 g three times a day for 14 days orally) started the day after each injection, with dose-adjustment for renal impairment. The trial was designed to incorporate current standard-of-care EBRT across all part ici pating sites. Patients received standard EBRT (78 Gy in 2 Gy fractions) or hypofractionated EBRT (60 Gy in 3 Gy fractions or 70 Gy in 2·5 Gy fractions). The protocol for this trial (PrTK03) included detailed specifications for clinical target volume and planned target volume, critical normal structures, and prescription dose. On Feb 7, 2019, the protocol was amended to allow the use of moderate hypofractionated EBRT to align with emerging changes in standard of care. Short-course ADT (4–6 months) was optional per the treating physician’s clinical judgement.

Patient assessments occurred at baseline, 1–2 weeks after each injection, 3, 6, 12, 18, and 24 months post-EBRT completion, and thereafter every 6 months through year 5. Long-term follow-up will continue for at least 10 years. Assessments included PSA measurements, digital rectal exams, prostate biopsies (baseline and approximately 2-years post-EBRT), QOL patient questionnaires (Extended prostate cancer index composite [EPIC]-26), acute toxicity monitoring 6-weeks post-study treatment completion (including safety laboratory assessments 1–2 weeks after each injection), and late toxicity monitoring 5-years post-EBRT. Adverse events were graded using common terminology criteria for adverse events (CTCAE) criteria, version 4.0.^22,23^ Treatment-emergent adverse events (TEAEs) were defined as any adverse event with onset after the administration of study medication (ie, the first aglatimagene or placebo injection) until 30 days after the last administration of study treatment, or any event that was present at baseline, but worsened in intensity through 30 days after the last administration of study treatment. MedDRA version 27.1 was used for coding TEAEs.

An independent, unmasked Data Safety Monitoring Board reviewed toxicity and trial progress and a masked adjudication committee assessed causes of death (appendix pp 2–3). A masked central pathology review was done on all prostate biopsies, following two readers plus one adjudicator methodology: confirmation by two readers was required to establish presence of tumour cells (appendix p 12). Patients could discontinue the trial at any time at their own request or at the investigator’s discretion due to safety concerns or non-compliance. Follow-up continued for clinical outcomes unless consent was withdrawn. The trial was conducted under a Special Protocol Assessment agreed with the US FDA.

### Outcomes

The primary endpoint was disease-free survival in the intent-to-treat (ITT) population, defined as time-from-randomisation to an event of local (including positive biopsy) or regional failure, distant metastases, or all-cause death (or most recent follow-up if no event occurred); these events are defined in the statistical analysis plan (SAP; appendix). Cause of death was determined by site investigator. A predefined secondary endpoint was analysis of prostate cancer-specific disease-free survival that excluded deaths not associated with prostate cancer. Secondary endpoints included PSA nadir 0·2 ng/mL or less, freedom from biochemical failure defined as PSA nadir plus 2 ng/mL, prostate cancer-specific survival, overall survival, immune stimulation, and QOL up to 2 years post-treatment.^24^ Analysis of freedom from biochemical failure rate was not conducted due to the small number of events. Immune studies were not performed in this trial due to operational challenges encountered in the collection of biosamples.

### Statistical analysis

For sample size calculation, using the primary endpoint (disease-free survival in the ITT population) with a 2:1 randomisation ratio, around 98 events would provide 90% power to detect a hazard ratio (HR) of 0·5 for prostate cancer recurrence using stratified log-rank test at alpha level=0·05. It was estimated that 711 randomised patients would provide the required disease-free survival events.^10^ All statistical tests were two-sided; p values of 0·05 or less indicated statistical significance. Curves for disease-free survival were estimated using Kaplan–Meier methods. The proportional hazards assumption was evaluated using Schoenfeld residuals.

For duration of disease-free survival, Cox multivariable regression model was used to estimate the hazard of disease-free survival between groups, adjusting for stratification factors at significance level of 5%. All patients were censored at the last trial visit at the time of analysis unless they had a disease-free survival event. Sensitivity analyses using the per-protocol population, unstratified analytical methods, and multiple supplementary estimands were performed to evaluate the robustness of the primary estimand by different strategies for handling intercurrent events, such as initiation of new anticancer therapy, death before recurrence assessment, extended loss to follow-up, and missed consecutive visits. The per-protocol population was patients who completed all three courses of aglatimagene or placebo plus valacyclovir (≥7 days) and radiation therapy with or without ADT, and excluded patients with major treatment deviations (eg, those who received long term ADT, pelvic or lymph node radiation, or brachytherapy).

Predefined subgroup analyses based on the stratification factors and baseline characteristics (ie, NCCN risk groups, ADT use, age, race, and ethnicity) were conducted. Treatment effects within subgroups were assessed using Cox proportional hazards models. HRs and 95% CIs were estimated within each subgroup. Median follow-up was obtained by inverse Kaplan–Meier method. Details of the handling of missing data can be found in the SAP (appendix). The ITT population included all randomly assigned patients and was used for baseline and efficacy analyses; the safety population included all patients who received at least one administration of aglatimagene or placebo.

An exploratory, post-hoc analysis of pathological complete response based on central blinded evaluation was conducted, defined as negative biopsy (absence of tumour cells) in the 2-year biopsies performed at 22–26 months post EBRT. Proportions were compared between arms using the χ^2^ test.

As a post-hoc analysis, patients within the intermediate risk group were classified as having favourable or unfavourable intermediate risk disease according to NCCN criteria. Favourable intermediate risk disease was defined by the presence of only one intermediate-risk factor, Gleason grade group 1 or 2 (Gleason score 3 plus 3 or 3 plus 4), and fewer than 50% positive biopsy cores. Unfavourable intermediate risk disease was defined by the presence of two or three intermediate risk factors, Gleason grade group 3 (Gleason score 4 plus 3), or 50% or more positive biopsy cores.

Adverse events were summarised by patient incidence by category, term, and severity. In any tabulation, a patient contributes only once to the count. A post-hoc analysis compared safety between patients aged ≥65 years and <65 years, and was not prespecified; results are descriptive. The mixed-effects model for repeated measures (MMRM) including treatment, visit (as categorical), treatment by visit, stratification factors as fixed effects, baseline score as a covariate, and participants being the random effect, was used to estimate changes in QOL score over time. p values from comparison of least-square-mean estimates from two treatment groups were provided from MMRM. Missing scores were not imputed using the MMRM approach assuming an unstructured variance-covariance matrix for the repeated measures.

We used SAS statistical analysis program version 9.4. The full protocol and SAP are in the appendix.

### Role of the funding source

The study sponsor Candel Therapeutics designed and conducted the trial in collaboration with principal investigators. Candel Therapeutics employees contributed to study design, clinical site operations, data collection, data analysis, data interpretation, and writing of this manuscript. The US National Institutes of Health grant (CA124032) was used for study design and clinical site operations.

## Results

**Figure 1:**
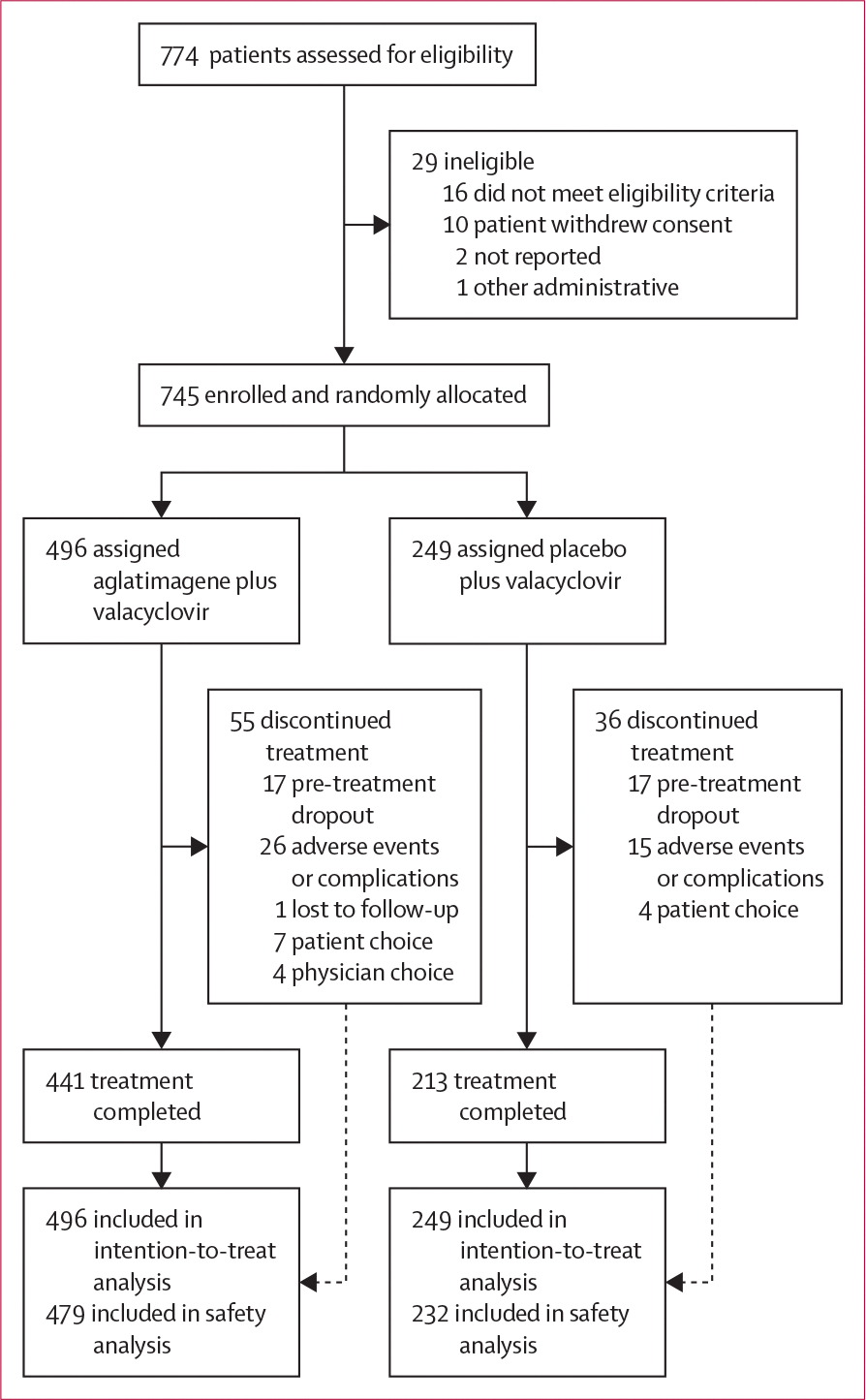
Trial profile

Between Feb 21, 2012, and Sept 9, 2021, of 774 men screened, 745 were randomly assigned to receive aglatimagene (n=496) or placebo (n=249) plus valacyclovir at 51 centres across the USA and Puerto Rico (appendix pp 4–6). The ITT population included all 745 randomly-assigned patients and the safety population included 711 patients who received at least one injection of aglatimagene or placebo. Overall, 441 (90%) of 496 patients on the aglatimagene arm and 213 (86%) of 249 on the placebo arm completed treatment (figure 1). Median follow-up was 50·3 months (IQR 35·2–63·3).

Baseline characteristics are shown in table 1. Median age was 69 years (IQR 64–73), 591 (79%) of 745 patients were White, 121 (16%) were Black or African American (representative of the population with prostate cancer in the USA), 25 (5%)did not report this information, four (1%) were Asian, two (<1%) were American Indian or Alaskan Native, and two (<1%) were Native Hawaiian or other Pacific Islander. At baseline, 635 (85%) of 745 patients were staged as having intermediate-risk disease, 239 (38%) of these 635 patients had favourable intermediate-risk disease and 396 (62%) had unfavourable intermediate-risk disease. 110 (15%) of 745 patients were staged as having high-risk disease. Median PSA level was 6·7 ng/mL (IQR 5·1–9·9), and most patients (634 [85%] of 745) had a Gleason score of 7 (409 [55%] with a score of 3 plus 4 [grade group 2] and 225 [30%] with a score of 4 plus 3 [grade group 3]). Short-course ADT was planned for 366 (49%) of 745 patients.

645 [91%] of 711 treated patients received all three injections of aglatimagene or placebo and at least 7 days of valacyclovir after each injection (appendix pp 8–9). All 711 patients received EBRT, with 515 (72%) receiving standard-fractionated EBRT (78 Gy median dose) and 177 (25%) receiving moderate-hypofractionated EBRT (60 Gy median dose), and 19 (3%) were not reported. Overall, 294 (41%) of 711 patients received ADT for 1–6 months, including 76 (72%) of 105 in the high-risk category, whereas 349 (58%) of 606 patients in the intermediate-risk category received no ADT (appendix p 10; see appendix p 11 for ADT exposure in the ITT population). ADT was administered for more than 6 months in 18 (17%) of 105 patients in the high-risk category and 22 (4%) of 606 patients in the intermediate-risk category.

**Figure 2:**
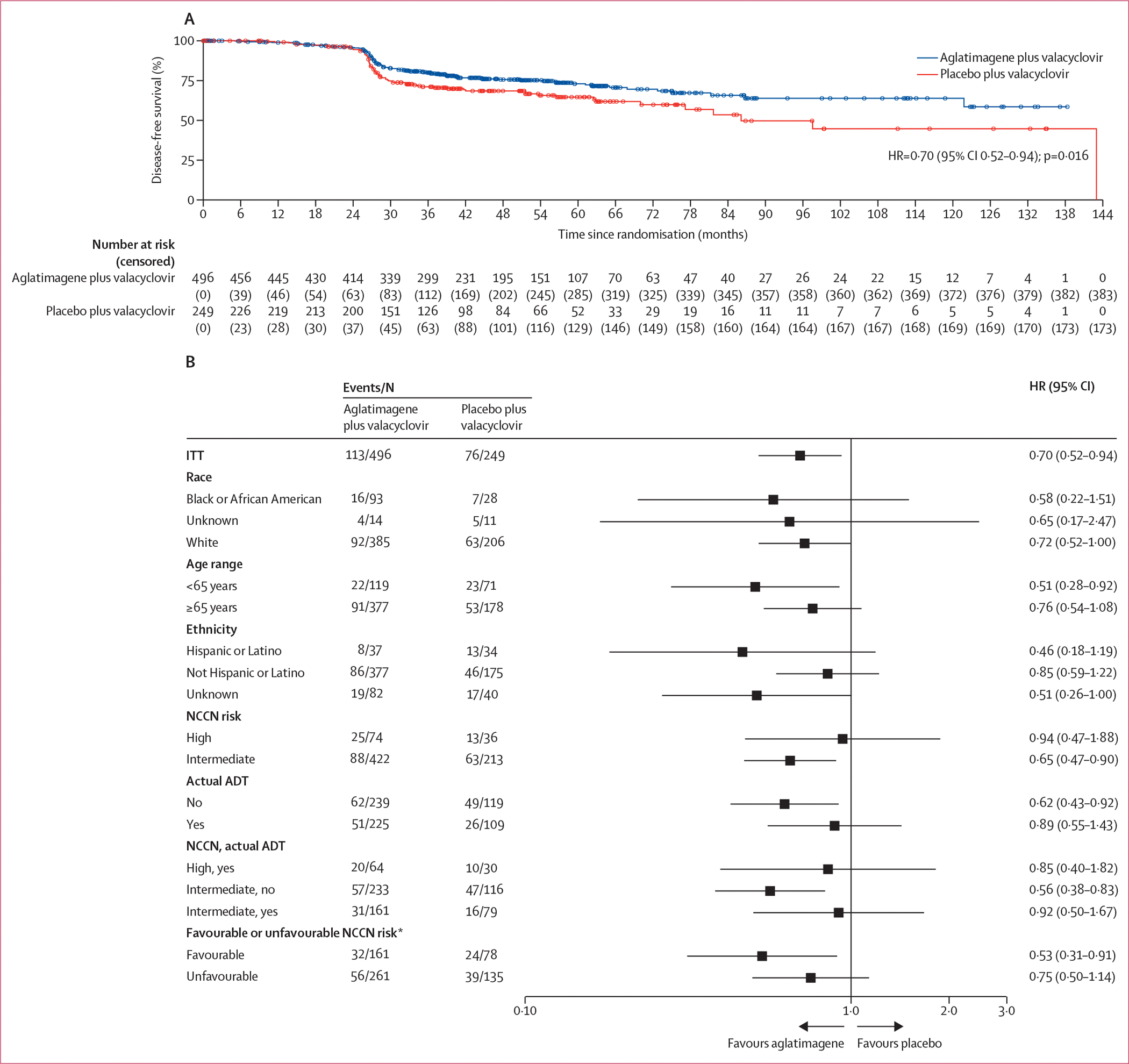
Disease-free survival by treatment group and subgroups (ITT population) (A) Kaplan–Meier curve of disease-free survival (ITT population). The p value was computed from log-rank test using stratification factors (risk category intermediate/high and androgen-deprivation therapy use yes/no) at randomisation. (B) Forest plot of HR and 95% CI for disease-free survival by subgroups (ITT population). HR was calculated from Cox regression model considering stratified factors from randomisation. Due to the small number of events between treatment groups, there is no HR for: race subgroups American Indian or Alaska Native, Native Hawaiian or Other Pacific Islander, or Asian; NCCN and planned ADT use high, no; or NCCN and actual ADT use high, no. ADT=androgen-deprivation therapy. HR=hazard ratio. ITT=intent-to-treat. NCCN=National Comprehensive Cancer Network. *Post-hoc-analysis.

In the primary efficacy analysis (ITT population), aglatimagene was associated with longer disease-free survival than placebo (stratified HR 0·70; 95% CI 0·52–0·94; stratified log-rank p=0·016; figure 2). Disease-free survival events occurred in 113 (23%) of 496 patients in the aglatimagene group and 76 (31%) of 249 patients in the placebo group over a median 50·3-month follow-up (IQR 35·2–63·3). Median disease-free survival was not reached (95% CI 121·78 to not reached) in the aglatimagene group versus 86·1 months (IQR 29·7–143·0) in the placebo group. Sensitivity analyses and the use of supplementary estimands confirmed the robustness of the primary estimand (data not shown). No evidence of a violation of the proportional hazards assumption was observed. Prespecified subgroups showed similar results, although many subgroups were small resulting in wide confidence intervals that included one (figure 2). Results from a post-hoc exploratory subgroup analysis in the intermediate-risk population classified as favourable and unfavourable is shown in figure 2.

**Figure 3:**
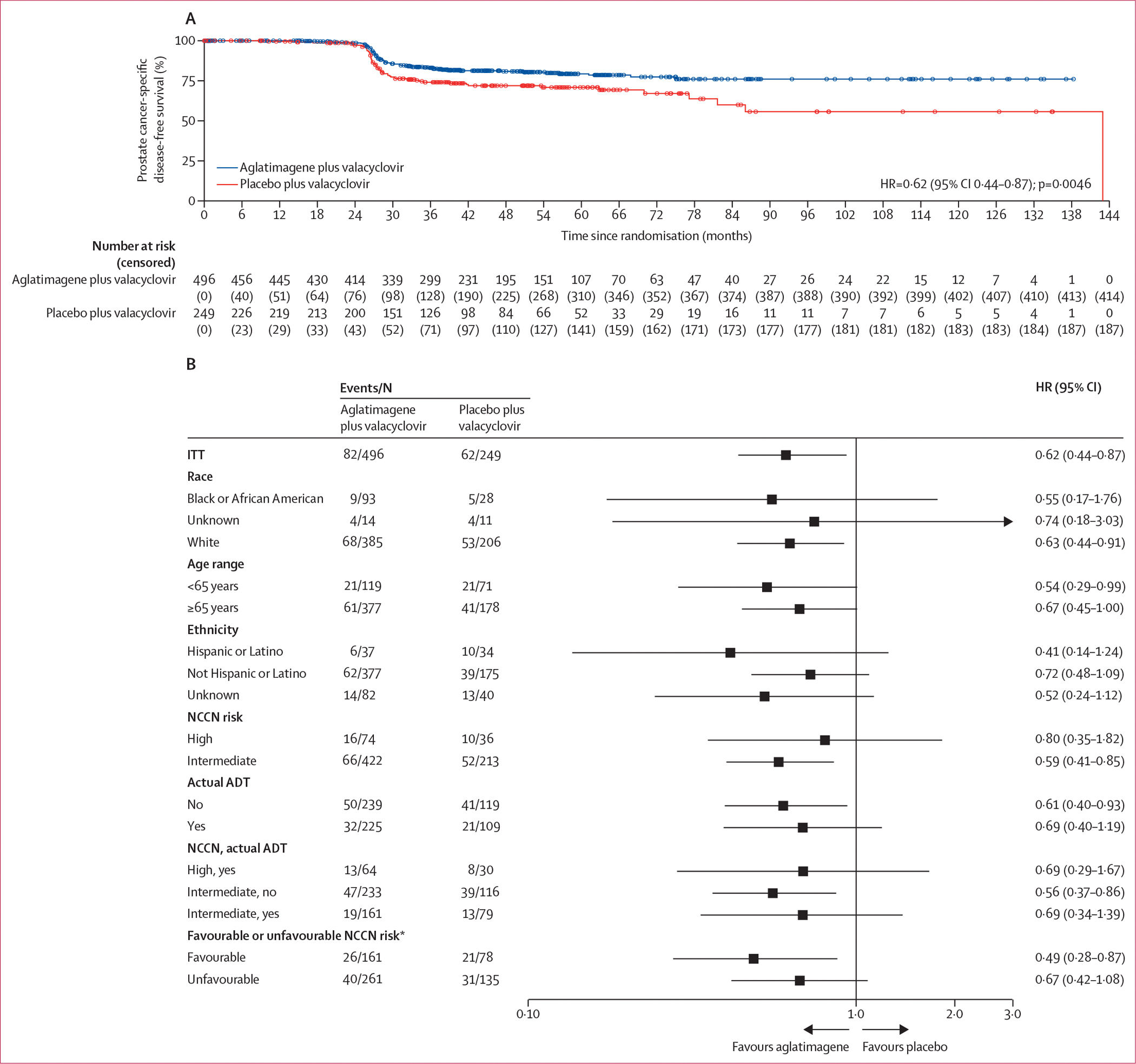
Prostate cancer-specific disease-free survival (time to prostate cancer recurrence or death due to prostate cancer) by treatment group (ITT population) (A) Kaplan–Meier curve of prostate-specific disease-free survival (ITT population). The p value was computed from log-rank test using stratification factors (risk category intermediate/high and androgen-deprivation therapy use yes/no) at randomisation. (B) Forest plot of HR and 95% CI for prostate-specific disease-free survival by subgroup (ITT population). HR was calculated from cox regression model considering stratified factors from randomisation. Due to the small number of events between treatment groups, there is no HR for: race subgroups American Indian or Alaska Native, Native Hawaiian or Other Pacific Islander, or Asian; NCCN and planned ADT use high, no; or NCCN and actual ADT use high, no. ADT=androgen-deprivation therapy. HR=hazard ratio. ITT=intent-to-treat. NCCN=National Comprehensive Cancer Network. *Post-hoc analysis.

For prostate cancer-specific disease-free survival (ITT population), aglatimagene showed improvement over placebo (stratified HR 0·62; 95% CI 0·44–0·87; stratified log-rank p=0·0046; figure 3). Prostate cancer-specific disease-free survival events occurred in 82 (17%) of 496 patients in the aglatimagene group and 62 (25%) of 249 patients in the placebo group; median disease-free survival was not reached (95% CI not reached to not reached) in the aglatimagene group, versus 143·0 months (IQR 34·2–143·0) in the placebo group. Exploratory analyses prostate cancer-specific disease-free survival in prespecified subgroups are shown in figure 3. Post-hoc analysis of the intermediate-risk group further classified as favourable and unfavourable and with and without ADT are shown in appendix (p 14).

Patients with disease recurrence subsequently underwent salvage anticancer treatments that included ADT and other hormone therapies (ie, leuprolide, enzalutamide, and abiraterone), prostatectomy, radiation therapy, and experimental treatments in clinical trials (appendix p 13).

The proportion of patients achieving PSA nadir of 0·2 ng/mL or less was 333 (67%) in the aglatimagene group compared with 146 (59%) in the placebo group (p=0·016). Prostate cancer-specific overall survival was not estimated as there were only two prostate cancer-related deaths. At a median follow-up of 50·2 months (IQR 37·0–63·7), overall survival was comparable between the aglatimagene and placebo groups (stratified HR 1·18 [95% CI 0·64–2·15]; stratified log-rank p=0·59; appendix p 15), median overall survival was not reached in either group at the time of anaylsis (95% CI not reached to not reached for both groups). No deaths were attributed to study drug by the masked adjudicators or investigators. Deaths were distributed across organ systems in both groups with no temporal relationship to study drug injections (appendix p 16). In the aglatimagene group, there were 36 (7%) deaths (one of which was related to prostate cancer), most commonly from the infections and infestation system organ class (eight [2%]), followed by general disorders and admininstration site conditions (seven [1%]), neoplasms (seven [1%]), cardiac disorders (five [1%]), nervous system disorders (four [1%]), injury, poisoning, and procedural complications (three [1%]), respiratory, thoracic, and mediastinal disorders (one [<1%]), and social circumstances (one [<1%]). In the placebo group, there were 15 (6%) deaths (one of which was related to prostate cancer), primarily due to general disorders and admininstration site conditions (five [2%]), followed by neoplasms (four [2%]), cardiac disorders (three [1%]), infections and infestations (one [<1%]), neoplasms (one [<1%]), and renal and urinary disorders (one [<1%]; appendix p 16). Two deaths (one in each treatment group) were due to prostate cancer (appendix pp 17–19); both patients were in the NCCN high-risk group.

Overall, 451 (61%) of 745 patients completed the 2-year post-treatment biopsy; in 313 (42%) of patients the biopsy was done at the 22–26-month timepoint (appendix p 20). Aglatimagene improved pathological complete response rates in a post-hoc blinded review of 2-year post-EBRT evaluable biopsies (excluding 6 indeterminate biopsies) done at the 22–26-month timepoint, with negative biopsies in 167 (80%) of 209 patients in the aglatimagene group and 62 (63%) of 98 patients in the placebo group (χ^2^ p=0∙0018; appendix p 20). Reasons for patients not completing the 2-year biopsy included patient refusal, medical reasons, previous disease-free survival event, study discontinuation, COVID-19 pandemic, and lost to follow-up.

Overall, 626 (88%) of 711 patients had treatment-emergent adverse events. Most TEAEs (in 569 [80%] of 711 patients) were grades 1–2, and no grade 5 TEAEs (deaths) were reported. TEAEs of grade 3 or worse were similar and infrequent in both groups: 40 (8%) of 479 patients in aglatimagene group versus 17 (7%) of 232 patients in the placebo group, with the most common being acute kidney injury occurring in 2% of each group (nine of 479 patients in the aglatimagene group and four of 232 patients in the placebo group; table 2; appendix pp 21–27).

Study treatment-related TEAEs were reported in 477 (67%) of 711 patients (table 2). The most common treatment-related TEAEs occurring in at least 10% of patients in either treatment group were chills, influenza-like symptoms, pyrexia, pollakiuria, and nausea; table 3). Chills and influenza-like symptoms were self-limiting, resolving within 24–72 h. The most common grade 3–4 treatment-related TEAE was acute kidney injury, occurring in five (1%) of 479 patients in the aglatimagene group and three (1%) of 232 patients in the placebo group. All other grade 3–4 treatment-related adverse events occurred in 1% or fewer patients in both groups (table 3).

The most frequently reported injection procedure-related adverse events overall were urinary urgency and urinary tract pain. Urinary tract infections considered related to experimental treatment were observed in seven (2%) patients in the aglatimagene group and four (2%) patients in the placebo group (table 3).

Serious adverse events occurred in 28 (6%) of 479 patients in the aglatimagene group versus 17 (7%) of 232 in the placebo group, and treatment-related serious adverse events occurred in eight (2%) versus five (2%) patients (table 2; appendix pp 29–30). Treatment-related serious adverse events in the aglatimagene group were acute kidney injury (four [1%] of 479 patients), pyrexia (two [<1%]), influenza-like symptoms (one [<1%]), and urinary retention (one [<1%]) and in the placebo group were acute kidney injury (four [2%] of 232 patients), increased blood creatinine concentrations (one [<1%]), and skin rash (one [<1%]; one patient reported two separate serious adverse events; appendix p 31). There were similar rates of toxicities typically associated with radiotherapy across treatment groups, including renal and urinary, gastrointestinal, and reproductive system TEAEs (table 2). A post-hoc analysis showed no clinically significant differences in harms (TEAE incidence and severity of grade 3 or worse) between age groups of 65 years and older versus younger than 65 years (data not shown). Patient-reported QOL results were similar between treatment groups over 2 years (appendix pp 32–33).

## Discussion

The primary goal of curative-intent treatment (either radical prostatectomy or radiotherapy) for patients with localised prostate cancer is to eradicate the tumour, consistent with NCCN Clinical Practice Guidelines and European Association of Urology Guidelines. However, approximately 30% of patients with intermediate-risk to high-risk prostate cancer who undergo radical treatment will have disease recurrence, with salvage therapies often associated with significant side-effects and decreased QOL without a definitive cure.^4,5^ Previous studies have shown that local disease recurrence at least 2 years post-radical therapy is associated with a highly significant increased risk of metastatic disease and prostate cancer-related mortality after median follow-up of around 10 years.^3^ Although a 2-year biopsy is not part of standard clinical practice, it has previously been used as a gold standard research tool in clinical trials of radiation therapy with curative intent.^26,27^ Histological changes precede clinical evidence of recurrence. Accordingly, prostate biopsies positive for cancer cells are highly predictive of clinically meaningful outcomes including biochemical failure (10-times higher odds of developing biochemical failure [p<0·00001]), development of metastases (3-times higher odds of developing distant metastasis [p<0·00001]), and prostate cancer-specific mortality (5-times higher odds of dying from their prostate cancer [p<0·00001]) after extended follow up.^3^ Thus, histological detection of cancer represents an early and objective measure of disease recurrence.^27^

Research based on patient-completed surveys has shown the importance of the perception of being prostate cancer-free following treatment in patients who elect radical therapy for localised prostate cancer.^28^ These patients are often willing to accept the risk of long-term complications, including urinary incontinence and erectile dysfunction, hoping to achieve the most definitive oncological outcome. Fear of recurrence and cancer-related anxiety are common in patients with localised prostate cancer and are often increased after biochemical failure following radical therapy.^29^ Therefore, the aim of this study was to evaluate whether the addition of aglatimagene plus valacyclovir to standard-of-care radiotherapy could increase the proportion of patients with disease-free survival in those who seek curative treatment upon diagnosis of localised prostate cancer. The choice for this primary endpoint was a central component of the Special Protocol Assessment agreed with the FDA.

This phase 3 clinical trial showed a statistically significant and clinically meaningful improvement in disease-free survival for aglatimagene plus radiotherapy versus radiotherapy alone (HR 0·70, 95% CI 0·52–0·94; stratified log-rank p=0·016). The rate of recurrence after EBRT observed in the placebo group is comparable to that reported in similar patient populations.^5,6^ Some other clinical trials reported lower risk of recurrence after standard-of-care radiotherapy, which might be explained by differences in patient selection, including risk profile and ethnic background.^30–32^ As expected, mortality in this population of older men during the median 50·3-month follow-up was mainly due to causes not related to prostate cancer; there were only two deaths due to prostate cancer (one per treatment group).^33^ Prostate cancer is typically a slow-growing tumour, and the impact of potential cure on prostate cancer-related mortality is expected only after at least 10 years of follow-up.^3^ In addition, we report a highly significant effect on prostate cancer-specific outcomes, with improvement in prostate cancer-specific disease-free survival in the aglatimagene group compared with placebo. There was a significant increase in the proportion of patients achieving PSA nadir of 0·2 ng/mL or less in the aglatimagene group compared with placebo. Previous studies have shown that PSA levels at 6-months post-radiotherapy in localised prostate cancer are predictive of subsequent metastasis-free survival, prostate cancer-specific mortality, and overall survival in patients treated with radiotherapy, independent of the use of short-term or long-term ADT.^34^ The clinical data in our trial were supported by histological analysis: aglatimagene significantly improved the rate of pathological complete response in 2-year biopsies compared with placebo in a post-hoc analysis, suggesting the cancer had been eradicated at a microscopic level, consistent with the previous phase 2a clinical trial.^16^

Aglatimagene plus valacyclovir was generally well tolerated. The aglatimagene injection is simple in the hands of the urologist or radiation oncologist, takes around 20 min in an outpatient clinic setting, does not require local anaesthesia, and is generally well tolerated by patients.^35^ Our trial shows that, taken together, aglatimagene plus valacyclovir appears to offer a favourable benefit–risk profile to patients with localised prostate cancer who elect to undergo radiotherapy with curative intent, with a significant decrease in tumour recurrence after only three treatment courses that are feasible and well tolerated.

Prevention of tumour recurrence is not only expected to reduce cancer-related anxiety due to recurrence but would also avoid the need for salvage therapies including ADT post-recurrence.^4^ The beneficial effect of aglatimagene plus valacyclovir in localised prostate cancer is consistent with recent encouraging overall survival data in a randomised controlled phase 2a clinical trial of aglatimagene plus valacyclovir in borderline resectable pancreatic ductal adenocarcinoma and an open-label phase 2a clinical trial of aglatimagene plus valacyclovir in therapy-resistant non-small-cell lung cancer.^36,37^ Together, these data support the clinical activity of this therapeutic regimen across different solid tumour types.

This trial has several limitations. First, the 50·3-month median follow-up makes it impossible to show the impact of aglatimagene on metastasis-free survival and overall survival in this study, which would require follow-up of approximately 10 years.^3,33^ However, previous work has consistently shown the relationship between local recurrence after at least 2 years and subsequent locoregional disease progression, metastatic disease, and prostate cancer-related mortality after longer follow-up.^3^ The primary goal of curative treatment for patients with localised prostate cancer is to eradicate the tumour. The data presented here suggests that this goal was achieved in a higher proportion of patients treated with aglatimagene than with placebo. Second, most patients with a single high-risk factor (15% of our study population) did not receive long-term ADT, as recommended by the most recent NCCN guidelines. Of note, on the basis of real-world studies, at least 30% of patients with localised, high-risk prostate cancer will not receive long-term ADT based on contraindications, side-effects, and patient preference.^38^ Third, the trial was not statistically powered for independent testing of the various subgroups. Finally, we did not measure neutralising antibodies against adenovirus in this trial. In a previous clinical study of aglatimagene in patients with various solid tumours, no correlation was found between antibody titre, safety, and antitumour response.^39^ These findings are consistent with our preclinical data showing that baboons treated with intratumoral aglatimagene who were negative for antiviral antibodies at baseline did not develop de novo antiviral antibodies following treatment.^40^ These findings suggest that the mechanism of action of aglatimagene, driven by tumour cell killing and subsequent activation of anti-tumour immunity, is not meaningfully impaired by baseline or induced humoral immunity to adenovirus. However, we cannot entirely exclude the possibility that neutralising antibodies might have enhanced or impaired the therapeutic efficacy of aglatimagene in this trial.^39–41^ Despite these limitations, the study has a major strength. To our knowledge, this study is the first, multicentre, randomised phase 3 clinical trial that achieved its primary and secondary endpoints in localised prostate cancer in more than 20 years, offering a potential framework shift in the treatment of patients with intermediate-risk to high-risk localised prostate cancer who seek cure. If approved, aglatimagene immunotherapy could represent, to the best of our knowledge, the first new therapy for men with localised prostate cancer in over 20 years.

## Supplementary Material

MMC1

## Figures and Tables

**Table 1:** Baseline characteristics (intent-to-treat population)

	Aglatimagene plus valacyclovir (n=496)	Placebo plus valacyclovir (n=249)

**Age, years**		
Median	69	68
IQR	65–73	47–73
**Age group**		
Age <65 years	119 (24%)	71 (29%)
Age ≥65 years	377 (76%)	178 (71%)
**Race**		
American Indian or Alaska Native	1 (<1%)	1 (<1%)
Asian	3 (1%)	1 (<1%)
Black or African American	93 (19%)	28 (11%)
Native Hawaiian or Other Pacific Islander	0	2 (1%)
White	385 (78%)	206 (83%)
Not reported	14 (3%)	11 (4%)
**Ethnicity**		
Hispanic or Latino	37 (7%)	34 (14%)
Not Hispanic or Latino	377 (76%)	175 (70%)
Not reported	82 (17%)	40 (16%)
**Baseline National Comprehensive Cancer Network risk category**		
High	74 (15%)	36 (14%)
Intermediate	422 (85%)	213 (86%)
Favourable intermediate*	161 (38%)	78 (37%)
Unfavourable intermediate*	261 (62%)	135 (63%)
**Baseline prostate-specific antigen, ng/mL**		
Median	6·815	6·500
IQR	5·000–9·920	5·130–9·920
**Gleason grade groups**		
Gleason grade group 1	19 (4%)	5 (2%)
Gleason grade groups 2 and 3	417 (84%)	217 (87%)
Gleason grade group 2	264 (53%)	145 (58%)
Gleason grade group 3	153 (31%)	72 (29%)
Gleason grade groups 4 and 5	60 (12%)	27 (11%)
**Tumour stage**		
T1c	357 (72%)	171 (69%)
T2a	73 (15%)	39 (16%)
T2b	37 (7%)	19 (8%)
T2c	29 (6%)	20 (8%)
T3a	0	0
**Planned androgen-deprivation therapy use**		
Yes	244 (49%)	122 (49%)
No	252 (51%)	127 (51%)
**Eastern Cooperative Oncology Group performance status**		
0	456 (92%)	233 (94%)
1	35 (7%)	16 (6%)
2	5 (1%)	0

Data are n (%) unless otherwise specified.

* The denominator for the percentages of patients with unfavourable or favourable disease is the number of patients in the intermediate-risk category. Gleason grade group defined according to the International society of Urological Pathology grading system: grade group 1 corresponds to Gleason score ≤6 (3+3), grade group 2 to Gleason 3+4=7, grade group 3 to Gleason 4+3=7, grade group 4 to Gleason score 8, and grade group 5 to Gleason scores 9–10).^25^

**Table 2:** Overview of harms and treatment-emergent adverse events (safety population)

	Aglatimagene plus valacyclovir (n=479)	Placebo plus valacyclovir (n=232)

Any TEAE	434 (91%)	192 (83%)
Study treatment-related TEAE	354 (74%)	123 (53%)
TEAE of grade ≤2	394 (82%)	175 (75%)
TEAE of grade ≥3	40 (8%)	17 (7%)
TEAE of grade 4	6 (1%)	2 (1%)
Treatment-emergent deaths	0	0
Any serious adverse event	28 (6%)	17 (7%)
Study treatment-related serious adverse event	8 (2%)	5 (2%)
Adverse event or serious adverse event leading to death	0	0
Discontinued treatment due to an adverse event or serious adverse event	26 (5%)	14 (6%)
Discontinued treatment due to a treatment-related adverse event or serious adverse event	18 (4%)	10 (4%)
Prodrug dose modification	54 (11%)	27 (12%)
TEAEs by system organ class of interest		
Renal and urinary disorders	275 (57%)	125 (54%)
Gastrointestinal disorders	211 (44%)	96 (41%)
Reproductive system disorders	18 (4%)	9 (4%)
Immune system disorders*	1 (<1%)	0

Data are n (%) patients. TEAE=treatment-emergent adverse event.

* One patient in the aglatimagene group had a TEAE of hypersensitivity, which was assessed by the investigator as unrelated to study drug and due to an allergic reaction to vancomycin (used to treat a urinary tract infection); the hypersensitivity resolved without complications.

**Table 3:** Treatment-related TEAEs reported in at least one patient in either treatment group (safety population)

	Aglatimagene plus valacyclovir (n=479)			Placebo plus valacyclovir (n=232)		
	Grade 1–2	Grade 3	Grade 4	Grade 1–2	Grade 3	Grade 4

Chills	160 (33%)	0	0	20 (9%)	0	0
Influenza like illness	144 (30%)	2 (<1%)	0	32 (14%)	0	0
Pyrexia	120 (25%)	0	0	9 (4%)	0	0
Fatigue	86 (18%)	1 (<1%)	0	33 (14%)	1 (<1%)	0
Pollakiuria	58 (12%)	0	0	34 (15%)	0	0
Nausea	53 (11%)	0	0	19 (8%)	0	0
Headache	44 (9%)	1 (<1%)	0	12 (5%)	0	0
Diarrhoea	30 (6%)	0	0	18 (8%)	0	0
Micturition urgency	19 (4%)	0	0	16 (7%)	0	0
Malaise	28 (6%)	0	0	5 (2%)	0	0
Haematuria	22 (5%)	0	0	10 (4%)	0	0
Urinary tract pain	18 (4%)	0	0	14 (6%)	0	0
Vomiting	26 (6%)	0	0	3 (1%)	0	0
Dizziness	17 (4%)	0	0	9 (4%)	0	0
Decreased appetite	13 (3%)	0	0	5 (2%)	0	0
Urinary retention	13 (3%)	1 (<1%)	0	4 (2%)	0	0
Urinary tract obstruction	11 (2%)	0	0	2 (<1%)	0	0
Arthralgia	10 (2%)	0	0	2 (<1%)	0	0
Hot flush	8 (2%)	0	0	4 (2%)	0	0
Rectal haemorrhage	8 (2%)	0	0	4 (2%)	0	0
Urinary tract infection	7 (2%)	0	0	4 (2%)	0	0
Abdominal pain	7 (2%)	0	0	3 (1%)	0	0
Acute kidney injury	0	4 (<1%)	1 (<1%)	1 (<1%)	3 (1%)	0
Constipation	7 (2%)	0	0	1 (<1%)	0	0
Muscular weakness	5 (1%)	1 (<1%)	0	1 (<1%)	1 (<1%)	0
Myalgia	5 (1%)	0	0	3 (1%)	0	0
Nasal congestion	4 (<1%)	0	0	3 (1%)	0	0
Hyperhidrosis	5 (1%)	0	0	1 (<1%)	0	0

Data are n (%). No grade 5 TEAEs (deaths) were reported. TEAE=treatment-emergent adverse event.

## Data Availability

Researchers can submit a methodologically sound proposal to access raw or analysed data from 9 months to 36 months after publication, directed to info@candeltx.com. Candel Therapeutics (the study Sponsor) will promptly review the request, determine whether the requested data can be shared, and will respond within 8 weeks of receiving the request. Patient-related data were collected as part of a clinical trial and could be subject to patient confidentiality restrictions. Any data that can be shared will be released via a material transfer agreement.

## Research in context

### Evidence before this study

Around 30% of men with localised prostate cancer treated with curative-intent radiotherapy have disease recurrence, often resulting in anxiety, disease progression, and the need for salvage therapies with substantial side-effects. Earlier clinical trials of aglatimagene besadenovec (aglatimagene or CAN-2409), an adenoviral-based immunotherapy delivered with a prodrug (valacyclovir), showed promising antitumour activity across multiple solid tumours, including prostate cancer.

### Added value of this study

This phase 3, randomised, double-blind, placebo-controlled trial (n=745) evaluated the effect of aglatimagene plus prodrug in addition to standard-of-care external beam radiotherapy on disease-free survival in patients with localised, intermediate-risk or high-risk prostate cancer. Aglatimagene was associated with longer disease-free survival than placebo (hazard ratio 0·70, 95% CI 0·52–0·94; p=0·016), with secondary endpoints supporting the primary outcome.

### Implications of all the available evidence

Addition of aglatimagene to standard-of-care radiotherapy can improve disease-free survival in patients with localised prostate cancer, without clinically significant added toxicity. These findings support a potential framework shift in the management of intermediate-risk to high-risk localised prostate cancer, with the potential to reduce cancer-related anxiety, avoid salvage therapies associated with adverse effects, and improve long-term oncological outcomes.
