# Supplementary material for: Aglatimagene besadenovec (CAN-2409) with radiotherapy for patients with localised prostate cancer: a phase 3, multicentre, randomised, double-blind, placebo-controlled trial
Source: Lancet Oncol. Author manuscript; Available in PMC 2026 Aug 5. (PMC13437160; doi:10.1016/S1470-2045(26)00071-9)
Supplement: Supplementary file 1 [file NIHMS2186231-supplement-MMC1.pdf]

THE LANCET
Oncology

Supplementary appendix

This appendix formed part of the original submission and has been peer reviewed. We post it as supplied by the authors.

Supplement to: DeWeese TL, Manzanera A, Sylvester J, et al. Aglatimagene besadenovec (CAN-2409) with radiotherapy for patients with localised prostate cancer: a phase 3, multicentre, randomised, double-blind, placebo-controlled trial. *Lancet Oncol* 2026; **27:** 673–78.

Supplement to:

Aglatimagene besadenovec (CAN-2409) with radiotherapy for localized prostate cancer: a phase 3, randomised, placebo-controlled clinical trial

This appendix has been provided by the authors to give readers additional information about the clinical trial.

Table of Contents

Part 1

S1. List of DSMB members, central pathology review members, and adjudicators

S2. List of study centers

S3. Administration route of Aglatimagene besadenovec/Placebo

S4. Summary of Exposure overall and risk groups

S5. Overall Summary of Disease-Free Survival and Prostate Cancer-Specific Disease-Free Survival Events

S6. Prostate cancer-specific disease-free survival outcomes by ADT use and NCCN Subgroups for ITT population

S7. Kaplan-Meier Curve of Overall Survival (Intent-to-Treat Population)

S8. Summary of Cause of Death by System Organ Class (Intent-to-Treat Population)

S9. Summary of Two-Year Biopsies Results

S10. Summary of Safety

S11. Quality-of-Life (QoL) results: Change from baseline in EPIC-26 scores by treatment group

Part 2

PrTK03 Protocol

Part 3

PrTK03 Statistical Analysis Plan

S1. List of DSMB members, central pathology review members, and adjudicators

Independent, Unblinded Data Safety Monitoring Board (DSMB) Members:

- **Celestia Higano, MD**, Professor, Dept of Medicine and Urology, University of Washington, Seattle Cancer Care Alliance (DSMB chair)
- **Adam Kibel, MD**, Chief of Urology, Brigham and Women's Hospital, Dana Farber Cancer Institute (DSMB member)
- **Stuart Mushlin, MD**, Master Clinician, Brigham and Women's Hospital, Department of Medicine and Primary Care (DSMB Member)
- **Anthony Zietman, MD**, Jenet and William Shipley Professor of Radiation Oncology, Harvard Medical School and Massachusetts General Hospital (DSMB Member)
- **Scott Evans, PhD,** Professor Dept of Epidemiology and Biostatistics, Director of Biostatistics Center, Milken Institute of Public Health, George Washington University (DSMB member)

Blinded Central Pathology Review Members:

- **Thomas Wheeler, MD**, Distinguished Emeritus Professor, Department of Pathology & Immunology Baylor College of Medicine
- **Rajal Shah, MD**, Dr. Charles T. Ashworth Professor of Pathology, UT Southwestern Medical Center
- **Feng Deng, MD, PhD**, Director of Urologic Pathology, New York Langone Medical Center

Blinded Adjudication Members

- **Gopal Gupta, MD**, Professor of Urology, Surgery, and Radiology and Fellowship Director of Urologic Oncology, Loyola University Medical Center
- **Munveer Bhangoo, MD**, Staff Physician, Genitourinary/Gastrointestinal/Thoracic Oncology, Division of Hematology-Oncology, Scripps-MD Anderson Cancer Center

- **Kara Watts, MD**, Associate Professor of Urology, Montefiore Medical Center/Albert Einstein College of Medicine

S2. List of study centers

Principal Investigator	Institution	Enrollment (N=745)
John Sylvester	GenesisCare USA	112 (15)
Thomas Schroeder	University of New Mexico	83 (11)
Glen Gejerman	New Jersey Urology Research and Consulting LLC	81 (11)
Gregory Chesnut	Walter Reed National Military Medical Center	37 (5)
Joshua Meeks	Jesse Brown VA Medical Center	33 (4)
Thomas Facelle	Foothills Urology (Advanced Urology)	30 (4)
Christopher Pieczonka	Associated Medical Professionals	26 (4)
Nilay Gandhi	Potomac Urology Center	25 (3)
Mark Garzotto	VA Portland Health Care System	22 (3)
Steven Sukin	Texas Urology Specialists Sukin	22 (3)
Steven Kester	Clinical Research Center of Florida	19 (3)
Daniel Song	Johns Hopkins	19 (3)
Michael Liss	South Texas San Antonio VA	19 (3)
Ronald Tutrone	Chesapeake Urology Research Associates	18 (2)
Maximiliano Sorbellini	Southern Arizona VA Health Care System	18 (2)
Bryan Mehlhaff	Oregon Urology Institute	16 (2)
Paul Kim	Precision Radiation Oncology	16 (2)
Stephen Savage	Ralph H. Johnson Charleston VA	13 (2)
Shawn Zimberg	Integrated Medical Professionals	13 (2)
Osvaldo Padron	Florida Urology Partners	9 (1)
Andrew Trainer	Adult & Pediatric Urology, P.C.	8 (1)
Elizabeth Perazza	VA Caribbean Healthcare System	8 (1)

Principal Investigator	Institution	Enrollment (N=745)
Dana Nanigian	VA Northern California Health Care System	7 (1)
Michael Chang	Hunter Holmes McGuire VA Medical Center	7 (1)
Francis Cannizzo	Salem VA Medical Center	7 (1)
Cesar Ercole	James A. Haley Veteran's Hospital	6 (1)
Stanley Liauw	University of Chicago	6 (1)
Sanjay Mehta	Advanced Research Network	5 (1)
Eashwer Reddy	Kansas City VA Medical Center	5 (1)
Laurence Belkoff	MidLantic Urology	5 (1)
Barak Perahia	Sierra Nevada Health Care System VA	5 (1)
Sheldon Freedman	Sheldon Freedman MD, Ltd.	5 (1)
James Cochran	Urology Clinics of North Texas	5 (1)
Eric Horwitz	Fox Chase Cancer Center	4 (1)
Louis Krane	Southeast Louisiana Veterans Health Care System	4 (1)
Koushik Shaw	Austin Urology Institute	3 (<1)
David Beyer	Arizona Oncology Services	3 (<1)
Neal Shore	Carolina Urologic Research Center, LLC	3 (<1)
Brian Mazzarella	Urology Austin	3 (<1)
Mitchell Sokoloff	University of Massachusetts Worcester	2 (<1)
Peter Rossi	Valley View Hospital	2 (<1)
Manoj Reddy	VA North Texas Health Care System	2 (<1)
Justin Famoso	Arizona Center for Cancer Care	1 (<1)
Roscoe Nelson	Arizona Urology Specialists	1 (<1)
Jeffrey Gingrich	Durham VA Health Care System	1 (<1)
Robert Franklin	Cincinnati VA Medical Center	1 (<1)

Principal Investigator	Institution	Enrollment (N=745)
Jennifer Slim	MultiCare Health System	1 (<1)
Mohummad Siddiqui	VA Maryland Health Care System	1 (<1)
Michael Liss	The University of Texas Health Science Center at San Antonio	1 (<1)
Robert Given	Urology of Virginia, PLLC	1 (<1)
Glen McWilliams	James J. Peters VA Medical Center	1 (<1)

Abbreviations: VA= Veteran Affairs; LLC= Limited Liability Company; PLLC= Professional Limited Liability Company.

S3. Administration route of Aglatimagene besadenovec/Placebo

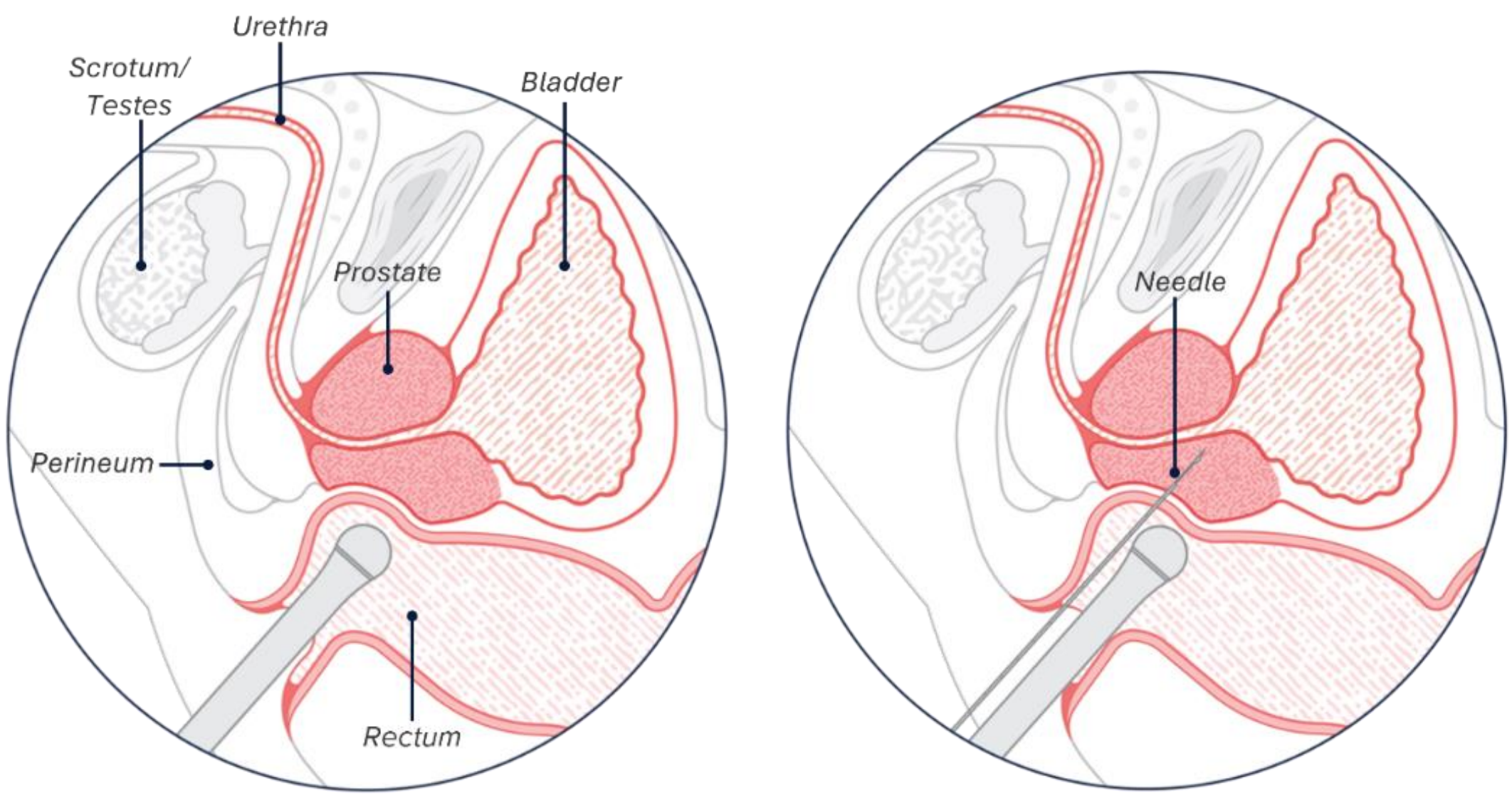

Transrectal ultrasound to visualize prostate and guide procedure

Transrectal (or transperineal) administration of CAN-2409 in four-0.5-mL injections into the four quadrants of the prostate via 22-gauge needle

S3: Patients received 3 treatment courses consisting of aglatimagene besadenovec (also known as CAN-2409) or placebo administered via ultrasound-guided intraprostatic-injections followed by 14 days of oral valacyclovir. All patients also received standard-of-care external-beam radiotherapy (EBRT). The 3 doses of aglatimagene/placebo were at least 14 days apart, with the first dose administered 2-8 weeks prior to EBRT start, the second dose 0-3 days prior to EBRT start, and the third dose 2-3 weeks after the second injection. Each dose of aglatimagene/placebo was administered in four-0.5-mL injections into the four quadrants of the prostate.

S4 (Part 1). Summary of Exposure to Treatments (Safety Population)

	Aglatimagene + Valacyclovir (N=479)	Placebo + Valacyclovir (N=232)	Total (N=711)
Number of patients, n (%) that			
Received 1 injection of aglatimagene/placebo	28 (6)	13 (6)	41 (6)
Received 2 injections of aglatimagene/placebo	10 (3)	6 (3)	16 (3)
Received 3 injections of aglatimagene/placebo	441 (93)	213 (92)	654 (92)
Completed treatment (3 injections of aglatimagene/placebo + ≥7 days of valacyclovir for each injection)	434 (91)	211 (91)	645 (91)
Number of patients, n (%) that received radiation			
Standard fractionated radiation, n (%)	341 (72)	174 (75)	515 (72)
Moderate hypofractionated radiation, n (%)	123 (26)	54 (24)	177 (25)
Unknown, n (%)	15 (4)	4 (2)	19 (3)
Total standard-EBRT dose received (Gy)			
n	341	174	515
Mean (STD)	79·04 (2·878)	79·09 (1·696)	79·06 (2·540)
Median	78·00	79·20	78·00
Min, Max	34·2, 86.4	71·4, 86·4	34·2, 86·4
Total moderate-EBRT dose received (Gy)			
n	123	54	177
Mean (STD)	64·42 (10)	64·91 (6)	64·57 (9)
Median	60·00	60·00	60·00

Min, Max	57·0, 155·6	60·0, 75·0	57·0, 155·6
Duration of ADT therapy, n (%)			
>1-6 months	197 (41)	97 (42)	294 (41)
>6-12 months	19 (4)	7 (3)	26 (4)
>12 months	9 (2)	5 (2)	14 (2)

Abbreviations: ADT=androgen-deprivation therapy; EBRT=extended-beam radiotherapy.

Duration of ADT therapy was based on patients' actual ADT exposure.

S4 (Part 2). Summary of Exposure to Treatments by Risk Group (Safety Population)

	Aglatimagene + Valacyclovir	Placebo + Valacyclovir	Total
Duration of ADT therapy, n (%)			
**High-risk category, N**	**72**	**33**	**105**
>1-6 months	51 (71)	25 (76)	76 (72)
>6-12 months	7 (10)	1 (3)	8 (8)
>12 months	6 (8)	4 (12)	10 (10)
No ADT	6 (8)	3 (9)	9 (9)
Unknown	2 (3)	0 (0)	2(2)
**Intermediate-risk category, N**	407	199	606
>1-6 months	146 (36)	72 (36)	218 (36)
>6-12 months	12 (3)	6 (3)	18 (3)
>12 months	3 (1)	1 (1)	4 (1)
No ADT	233 (57)	116 (58)	349 (58)
Unknown	13 (3)	4 (2)	17 (3)

Abbreviations: ADT=androgen-deprivation therapy.

S4 (Part 3). Summary of Exposure to Treatments by Risk Group (Intent-to-Treat Population)

	Aglatimagene + Valacyclovir	Placebo + Valacyclovir	Total
Duration of ADT therapy, n (%)			
**High-risk category, N**	74	36	110
>1-6 months	51 (71)	25 (70)	76 (69)
>6-12 months	7 (10)	1 (3)	8 (7)
>12 months	6 (9)	4 (12)	10 (10)
No ADT	6 (9)	3 (9)	9 (8)
Unknown	4 (3)	3 (8)	7 (6)
**Intermediate-risk category, N**	422	213	635
>1-6 months	146 (35)	72 (34)	218 (34)
>6-12 months	12 (3)	6 (3)	18 (3)
>12 months	3 (1)	1 (<1)	4 (1)
No ADT	233 (55)	116 (55)	349 (55)
Unknown	28 (7)	18 (9)	46 (7)

Abbreviations: ADT=androgen-deprivation therapy.

S5. Part 1 Overall Summary of Disease-Free Survival and Prostate Cancer-Specific Disease-Free Survival Events (Intent-to-Treat Population)

	Aglatimagene + Valacyclovir (N=496)	Placebo + Valacyclovir (N=249)	Total (N=745)
Number of patients with a DFS event, n (%)	113 (23)	76 (31)	189 (25)
Positive biopsy[1]	67 (14)	51 (21)	118 (16)
Treatment failure[2]	15 (3)	11 (4)	26 (4)
Death due to any cause[3]	31 (7)	14 (6)	45 (6)
Prostate cancer-related death[4]	0	0	0

Abbreviations: DFS=disease-free survival.

DFS event counts were based on the earliest events. Events were counted in the order of positive biopsy > treatment failure > death if multiple events happened on the same date.

1. Based on central blinded review of biopsies.

2. Treatment failure included local failure, regional failure, and distant metastases. In the aglatimagene group, the 15 patients with treatment failure included 2 with local failure, 6 with regional failure, and 7 with distant metastases. In the placebo group, the 11 patients with treatment failure included 4 with local failure, 1 with regional failure, and 6 with distant metastases.

3. Causes of death included cardiovascular, metabolic and chronic disease complications, COVID-19, other malignancy, and unknown causes.

4. Based on adjudicated cause of deaths. During the length of the trial, there were 2 prostate cancer-related deaths; however, patients had a prior event (disease recurrence) and therefore, these deaths are not counted as death event.

S5. Part 2 Summary of New anti-cancer treatments

New Anti-cancer Treatment	Aglatimagene + Prodrug (n)%	Placebo + Prodrug (n)%	Total
ADT/Hormonal	17 (77)	10 (63)	27
Radiation Therapy	1(5)	0 (0)	1
Prostatectomy	1(5)	1 (6)	2
Clinical Trial	1(5)	0 (0)	1
Other/Chemotherapy	2 (9)	5 (31)	7
**Total**	**22**	**16**	**38**

Abbreviations: ADT=androgen-deprivation therapy; Hormone therapies (i.e., leuprolide, enzalutamide, and abiraterone)

S6 PrTK03 Prostate cancer-specific disease-free survival outcomes by ADT use and NCCN Subgroups for ITT population

Use of ADT	NCCN Risk Category	N events / patients	Prostate specific HR with Aglatimagene
**No Androgen deprivation therapy**	Intermediate (overall)	86/349	HR = 0·56 95%, CI 0·37, 0·86
	Intermediate Favourable	44/188	HR = 0·49, 95% CI 0·27, 0·90
	Intermediate Unfavourable	42/161	HR = 0·66, 95% CI 0·36, 1·23
	High risk	5/9	Cannot be estimated
**Androgen deprivation therapy**	Intermediate (overall)	32/240	HR = 0·69, 95% CI 0·34 – 1·39
	Intermediate Favourable	3/31	HR = 0·64, 95% CI 0·06, 7·05
	Intermediate Unfavourable	29/209	HR = 0·68, 95% CI 0·32, 1·42
	High risk	21/94	HR = 0·69, 95% CI 0·29 – 1·67

Abbreviations: ADT=androgen-deprivation therapy; ITT=intent-to-treat; NCCN=National Comprehensive Cancer Network; HR: Hazard Ratio

S7. Kaplan-Meier Curve of Overall Survival (Intent-to-Treat Population)

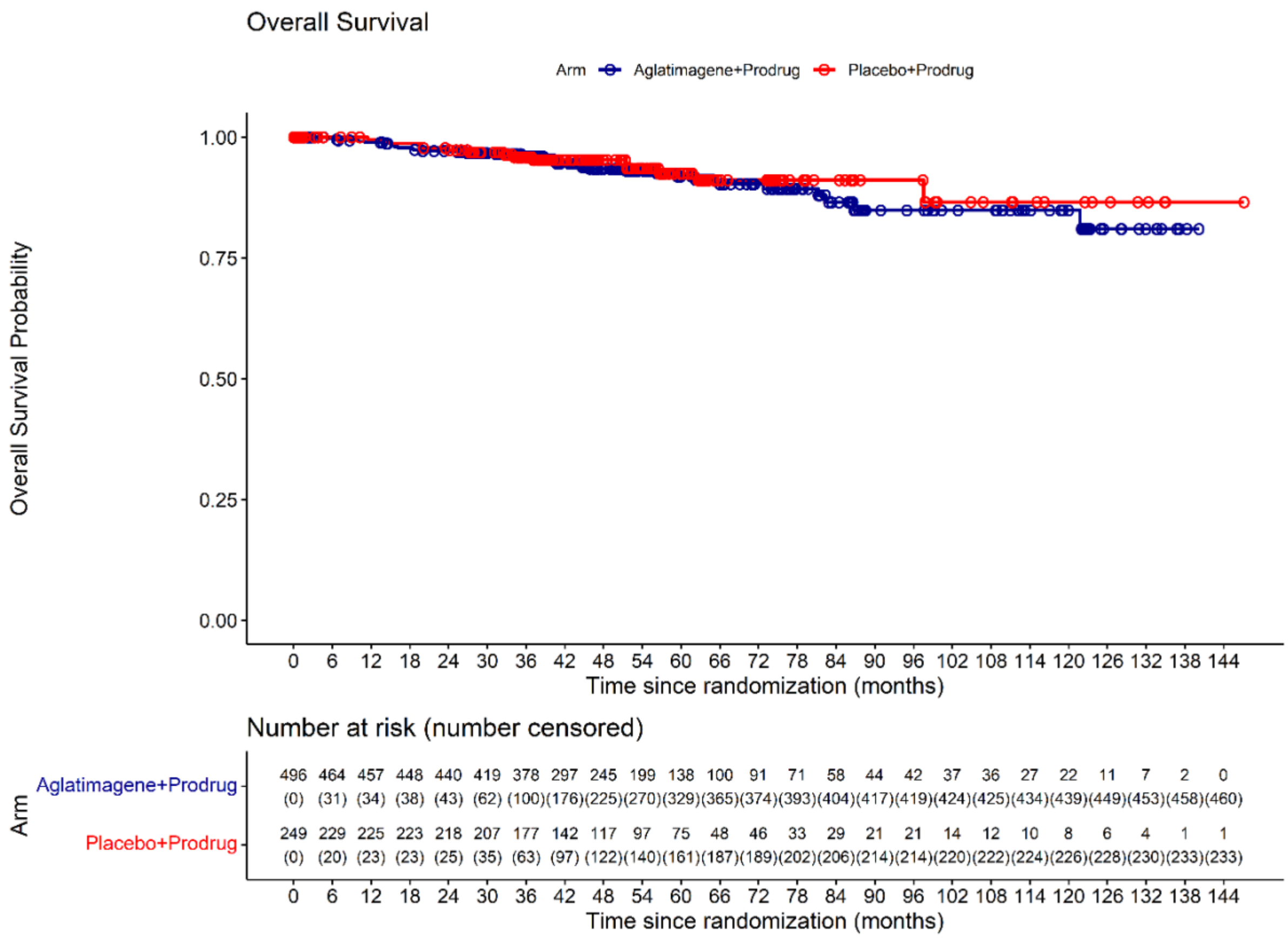

Abbreviations: ITT=intent-to-treat; Prodrug=valacyclovir.

The P-value was computed from log-rank test using stratification factors (risk category: intermediate/high and androgen-deprivation therapy use: yes/no) at randomization.

S8 (Part 1). Summary of Cause of Death by System Organ Class (Intent-to-Treat Population)[1]

	Aglatimagene + Valacyclovir (N=496)	Placebo + Valacyclovir (N=249)	Total (N=745)
**Cause of Death by System Organ Class, n (%)**			
Cardiac disorders	5 (1)	3 (1)	8 (1)
General disorders and administration site conditions[2]	7 (1)	5 (2)	12 (2)
Infections and infestations[3]	8 (2)	1 (<1)	9 (1)
Injury, poisoning and procedural complications	3 (1)	0 (0)	3 (<1)
Neoplasms benign, malignant and unspecified (including cysts and polyps)[4]	7 (1)	4 (2)	11 (2)
Nervous system disorder	4 (1)	1 (<1)	5 (1)
Renal and urinary disorders	0 (0)	1 (<1)	1 (<1)
Respiratory, thoracic and mediastinal disorders	1 (<1)	0 (0)	1 (<1)
Social circumstances	1 (<1)	0 (0)	1 (<1)
**Total deaths, n (%)**	36 (7)	15 (6)	51 (7)

MedDRA v27.1 dictionary was used for coding adverse event system organ class.

1. No deaths were determined to be causally related to study drug (aglatimagene/placebo + valacyclovir). No deaths occurred during the treatment period.

2. Includes 12 deaths of unknown cause.

3. Includes 5 deaths due to COVID-19.

4. Includes 2 deaths due to prostate cancer (1 death on each treatment arm)

S8 (Part 2). Patient-Level Listing of All Deaths by System Organ Class and Preferred Term[1]

System Organ Class<br>Subject ID	Treatment Group	Overall Survival (Months)[2]	Primary Cause of Death (Preferred Term)	Related to PCa
**Cardiac disorders**				
5633CV	aglatimagene + valacyclovir	18·02	Acute coronary syndrome	No
7571FV	placebo + valacyclovir	27·25	Cardiac arrest	No
5201CV	placebo + valacyclovir	51·55	Cardiac arrest	No
3353CF	aglatimagene + valacyclovir	56·25	Cardiac failure	No
1224NM	aglatimagene + valacyclovir	81·24	Cardiac failure	No
1394AU	aglatimagene + valacyclovir	121·78	Cardiac failure	No
7721NJ	aglatimagene + valacyclovir	15·16	Cardio-respiratory arrest	No
3511CV	placebo + valacyclovir	37·02	Myocardial infarction	No
**General disorders and administration site conditions[3]**				
3541CF	aglatimagene + valacyclovir	11·01	Death	No
1303NM	aglatimagene + valacyclovir	15·16	Death	No
6123OI	aglatimagene + valacyclovir	38·76	Death	No
6462HV	aglatimagene + valacyclovir	38·99	Death	No
1133AR	aglatimagene + valacyclovir	44·58	Death	No
3083CF	aglatimagene + valacyclovir	65·06	Death	No
2743NM	aglatimagene + valacyclovir	72·72	Death	No
5111TV	placebo + valacyclovir	14·83	Death	No
3693CF	placebo + valacyclovir	19·59	Death	No
3434CF	placebo + valacyclovir	19·99	Death	No
6014CR	placebo + valacyclovir	24	Death	No
3453CF	placebo + valacyclovir	56·55	Death	No
**Infections and infestations**				
5243CF	aglatimagene + valacyclovir	5·13	COVID-19	No

System Organ Class<br>Subject ID	Treatment Group	Overall Survival (Months)[2]	Primary Cause of Death (Preferred Term)	Related to PCa
7822TV	aglatimagene + valacyclovir	6·08	COVID-19	No
5714JV	aglatimagene + valacyclovir	6·71	COVID-19	No
5624NM	aglatimagene + valacyclovir	9·99	COVID-19	No
1201NM	aglatimagene + valacyclovir	86·73	COVID-19	No
5403VV	aglatimagene + valacyclovir	14·24	Pneumonia	No
3631UC	placebo + valacyclovir	62·53	Sepsis	No
1884NM	aglatimagene + valacyclovir	18·51	Septic shock	No
2254WR	aglatimagene + valacyclovir	82·45	Septic shock	No
**Injury, poisoning and procedural complications**				
1771AU	aglatimagene + valacyclovir	59·11	Craniocerebral injury	No
4671RV	aglatimagene + valacyclovir	24·16	Road traffic accident	No
5231BV	aglatimagene + valacyclovir	19·99	Toxicity to various agents[4]	No
**Neoplasms benign, malignant and unspecified (including cysts and polyps)**				
6533CU	aglatimagene + valacyclovir	16·11	Adenocarcinoma of colon	No
7741CF	placebo + valacyclovir	32·52	Gastrointestinal neoplasm	No
3981UC	aglatimagene + valacyclovir	40·34	Glioblastoma	No
2974SV	aglatimagene + valacyclovir	44·68	Glioblastoma	No
5303TV	placebo + valacyclovir	51·58	Hepatocellular carcinoma	No
2183WR	aglatimagene + valacyclovir	15·58	Lung carcinoma cell type unspecified recurrent	No
1031FC	aglatimagene + valacyclovir	40·8	Non-small cell lung cancer metastatic	No
1261AU	placebo + valacyclovir	97·55	Pancreatic carcinoma metastatic	No
3484AU	aglatimagene + valacyclovir	45·7	Prostate cancer	Yes
3134NM	placebo + valacyclovir	34·06	Prostate cancer	Yes
3563TV	aglatimagene + valacyclovir	40·6	Squamous cell carcinoma of lung	No

System Organ Class<br>Subject ID	Treatment Group	Overall Survival (Months)[2]	Primary Cause of Death (Preferred Term)	Related to PCa
**Nervous system disorder**				
4703AU	aglatimagene + valacyclovir	35·24	Cerebrovascular accident	No
3304CF	aglatimagene + valacyclovir	35·44	Cerebrovascular accident	No
8013ML	placebo + valacyclovir	11·47	Dementia	No
2724AU	aglatimagene + valacyclovir	62·24	Dementia Alzheimer's type	No
1692NM	aglatimagene + valacyclovir	51·42	Vascular dementia	No
**Renal and urinary disorders**				
4324AU	placebo + valacyclovir	13·68	Acute kidney injury	No
**Respiratory, thoracic and mediastinal disorders**				
5351BV	aglatimagene + valacyclovir	26·79	Respiratory failure	No
**Social circumstances**				
6493CU	aglatimagene + valacyclovir	30·05	Homicide	No

Abbreviations: PCa=prostate cancer.

MedDRA v27.1 dictionary was used for coding adverse event system organ class and preferred term.

1. No deaths were determined to be causally related to study drug (aglatimagene/placebo + valacyclovir). No deaths occurred during the treatment period.

2. Months from randomization

3. Includes deaths of unknown cause.

4. Cause of death was fentanyl toxicity.

S9 (Part 1). Summary of Two-Year Biopsies Results post EBRT (Intent-to-Treat Population)

2-year biopsies result	Aglatimagene + Valacyclovir (N=496)	Placebo + Valacyclovir (N=249)	Total (N=745)
**Total biopsies completed, n (%)**	**300 (60)**	**151 (61)**	**451 (61)**
Negative biopsy	225 (75)	97 (64)	322 (71)
Positive biopsy	67 (22)	51 (34)	118 (26)
Indeterminate biopsy	8 (3)	3 (2)	11 (2)

Includes all post EBRT biopsies. All biopsies were reviewed by independent blinded pathologists. Positive and negative biopsies required confirmation by 2 readers using the 2+1 methodology.

Abbreviations: EBRT=extended-beam radiotherapy.

S9 (Part 2). Summary of Two-Year Biopsies completed within 22-26 months post EBRT (Intent-to-Treat Population)

2-year biopsies result	Aglatimagene + Valacyclovir (N=496)	Placebo + Valacyclovir (N=249)	Total (N=745)
**Total biopsies completed at 22-26 months, n (%)**	**214 (43)**	**99 (40)**	**313 (42)**
Negative biopsy	167 (78)	62 (63)	229 (73)
Positive biopsy	42 (20)	36 (36)	78 (25)
Indeterminate biopsy	5 (2)	1 (1)	6 (2)

Biopsies were reviewed by independent blinded pathologists. Positive and negative biopsies required confirmation by 2 readers using the 2+1 methodology. For the calculation of the pathologic complete response, indeterminate biopsies (n=6) were excluded. Pathological complete response was observed in 167 (80%) of 209 patients in the aglatimagene group and 62 (63%) of 98 patients in the placebo arm (chi-square test p=0.0018)

Abbreviations: EBRT=extended-beam radiotherapy.

S10 Part 1 All Treatment-Emergent Adverse Events (Safety Population)

AE term	Aglatimagene + Valacyclovir arm (N=479)					Placebo + Valacyclovir arm (N=232)					Both arms (N=711)
	Grade 1-2 N (%)	Grade 3 N (%)	Grade 4 N (%)	Grade 5 N (%)	Total N (%)	Grade 1-2 N (%)	Grade 3 N (%)	Grade 4 N (%)	Grade 5 N (%)	Total N (%)	Total N (%)
Pollakiuria	178 (37%)	0	0	0	178 (37%)	93 (40%)	0	0	0	93 (40%)	271 (38%)
Fatigue	152 (32%)	1 (<1%)	0	0	153 (32%)	72 (31%)	1 (<1%)	0	0	73 (32%)	226 (32%)
Chills	164 (34%)	0	0	0	164 (34%)	21 (9%)	0	0	0	21 (9%)	185 (26%)
Influenza like illness	146 (30%)	2 (<1%)	0	0	148 (31%)	33 (14%)	0	0	0	33 (14%)	181 (26%)
Diarrhoea	103 (22%)	0	0	0	103 (21%)	51 (22%)	0	0	0	51 (22%)	154 (22%)
Pyrexia	125 (26%)	0	0	0	125 (26%)	9 (4%)	0	0	0	9 (4%)	134 (19%)
Micturition urgency	79 (17%)	0	0	0	79 (17%)	44 (19%)	0	0	0	44 (19%)	123 (17%)
Urinary tract pain	90 (19%)	0	0	0	90 (9%)	31 (13%)	0	0	0	31 (13%)	121 (17%)
Nausea	73 (15%)	0	0	0	73 (15%)	27 (12%)	0	0	0	27 (12%)	100 (14%)
Hot flush	49 (10%)	1 (<1%)	0	0	50 (10%)	20 (9%)	0	0	0	20 (9%)	70 (10%)
Headache	67 (14%)	1 (<1%)	0	0	68 (14%)	20 (9%)	0	0	0	20 (9%)	88 (12%)
Urinary tract obstruction	44 (9%)	1 (<1%)	0	0	45 (9%)	10 (4%)	1 (<1%)	0	0	11 (5%)	56 (8%)
Haematuria	37 (8%)	1 (<1%)	0	0	38 (8%)	15 (7%)	0	0	0	15 (7%)	53 (8%)
Urinary retention	42 (9%)	1 (<1%)	0	0	43 (9%)	10 (4%)	0	0	0	10 (4%)	53 (8%)
Constipation	32 (7%)	1 (<1%)	0	0	33 (7%)	16 (7%)	0	0	0	16 (7%)	49 (7%)
Vomiting	39 (8%)	0	0	0	39 (8%)	8 (4%)	0	0	0	8 (4%)	47 (7%)
Dizziness	26 (5%)	0	0	0	26 (5%)	16 (7%)	0	0	0	16 (7%)	42 (6%)
Malaise	31 (7%)	0	0	0	31 (7%)	5 (2%)	0	0	0	5 (2%)	36 (5%)
Decreased appetite	21 (4%)	0	0	0	21 (4%)	9 (4%)	0	0	0	9 (4%)	30 (4%)
Urinary tract infection	14 (3%)	3 (1%)	0	0	17 (4%)	11 (5%)	1 (<1%)	0	0	12 (5%)	29 (4%)
Abdominal pain	20 (4%)	1 (<1%))	0	0	21 (4%)	5 (2%)	1 (<1%)	0	0	6 (3%)	27 (4%)
Rectal haemorrhage	14 (3%)	0	0	0	14 (3%)	8 (3%)	0	0	0	8 (3%)	22 (3%)
Arthralgia	15 (3%)	0	0	0	15 (3%)	2 (1%)	0	0	0	2 (1%)	17 (2%)
Proctalgia	9 (2%)	0	0	0	9 (2%)	4 (2%)	0	0	0	4 (2%)	13 (2%)
Urinary incontinence	12 (3%)	0	0	0	12 (3%)	3 (1%)	0	0	0	3 (1%)	15 (2%)
Proctitis	9 (2%)	0	0	0	9 (2%)	5 (2%)	0	0	0	5 (2%)	14 (2%)
Back pain	12 (3%)	0	0	0	12 (2%)	2 (1%)	0	0	0	2 (1%)	14 (2%)
Muscular weakness	11 (2%)	1 (<1%)	0	0	12 (3%)	1 (<1%)	1 (<1%)	0	0	2 (1%)	14 (2%)

Confusional state	13 (3%)	0	0	0	13 (3%)	1 (<1%)	0	0	0	1 (<1%)	14 (2%)
Acute kidney injury	0	7 (2%)	2 (<1%)	0	9 (2%)	1 (<1%)	4 (2%)	0	0	5 (2%)	14 (2%)
Upper respiratory tract infection	8 (2%)	0	0	0	8 (2%)	4 (2%)	0	0	0	4 (2%)	12 (2%)
Insomnia	8 (2%)	0	0	0	8 (2%)	4 (2%)	0	0	0	4 (2%)	12 (2%)
Haemorrhoids	10 (2%)	0	0	0	10 (2%)	1 (<1%)	0	0	0	1 (<1%)	11 (2%)
Anal incontinence	9 (2%)	0	0	0	9 (2%)	1 (<1%)	0	0	0	1 (<1%)	10 (1%)
Myalgia	7 (1%)	0	0	0	7 (2%)	3 (1%)	0	0	0	3 (1%)	10 (1%)
Nasal congestion	7 (1%)	0	0	0	7 (2%)	3 (1%)	0	0	0	3 (1.3)	10 (1%)
Hyperhidrosis	8 (2%)	0	0	0	8 (2%)	1 (<1%)	0	0	0	1 (<1%)	9 (1%)
Hypotension	5 (1%)	1 (<1%)	0	0	6 (1%)	2 (1%)	1 (<1%)	0	0	3 (1%)	9 (1%)
Rash maculo-papular	4 (<1%)	0	0	0	4 (1%)	4 (2%)	0	0	0	4 (2%)	8 (1%)
Dehydration	4 (<1%)	2 (<1%)	0	0	6 (1%)	1 (<1%)	0	0	0	1 (<1%)	7 (1%)
Dyspnoea	6 (1%)	1 (<1%)	0	0	7 (2%)	0	0	0	0	0	7 (1%)
Oedema peripheral	4 (<1%)	0	0	0	4 (1%)	2 (1%)	0	0	0	2 (1%)	6 (1%)
Pain	4 (<1%)	0	0	0	4 (1%)	2 (1%)	0	0	0	2 (1%)	6 (1%)
Hyperglycaemia	1 (<1%)	3 (1%)	1 (<1%)	0	5 (1%)	1 (%)	0	0	0	1 (<1%)	6 (1%)
Arthritis	3 (<1%)	1 (<1%)	0	0	4 (1%)	2 (1%)	0	0	0	2 (1%)	6 (1%)
Pain in extremity	6 (1%)	0	0	0	6 (1%)	0	0	0	0	0	6 (1%)
Rhinitis allergic	3 (<1%)	0	0	0	3 (1%)	3 (1%)	0	0	0	3 (1%)	6 (1%)
Abdominal distension	3 (<1%)	0	0	0	3 (1%)	2 (1%)	0	0	0	2 (1%)	5 (1%)
Dysgeusia	5 (1%)	0	0	0	5 (1%)	0	0	0	0	0	5 (1%)
Syncope	0	4 (1%)	0	0	4 (1%)	0	1 (<1%)	0	0	1 (<1%)	5 (1%)
Ejaculation disorder	3 (<1%)	0	0	0	3 (1%)	2 (1%)	0	0	0	2 (1%)	5 (1%)
Erectile dysfunction	3 (<1%)	0	0	0	3 (1%)	2 (1%)	0	0	0	2 (1%)	5 (1%)
Cough	3 (<1%)	0	0	0	3 (1%)	2 (1%)	0	0	0	2 (1%)	5 (1%)
Sinus tachycardia	2 (<1%)	0	0	0	2 (<1%)	2 (1%)	0	0	0	2 (1%)	4 (1%)
Dry mouth	3 (<1%)	0	0	0	3 (1%)	1 (<1%)	0	0	0	1 (<1%)	4 (1%)
Flatulence	2 (<1%)	0	0	0	2 (<1%)	2 (1%)	0	0	0	2 (1%)	4 (1%)
Gait disturbance	3 (<1%)	0	0	0	3 (1%)	0	1 (<1%)	0	0	1 (<1%)	4 (1%)
COVID-19	2 (<1%)	1 (<1%)	0	0	3 (1%)	1 (<1%)	0	0	0	1 (<1%)	4 (1%)
Cystitis	3 (<1%)	0	0	0	3 (1%)	1 (<1%)	0	0	0	1 (<1%)	4 (1%)
Pneumonia	2 (<1%)	1 (<1%)	0	0	3 (1%)	0	1 (<1%)	0	0	1 (<1%)	4 (1%)
Flank pain	4 (<1%)	0	0	0	4 (1%)	0	0	0	0	0	4 (1%)

Depression	3 (<1%)	0	0	0	3 (1%)	1 (<1%)	0	0	0	1 (<1%)	4 (1%)
Hallucination	3 (<1%)	0	0	0	3 (1%)	1 (<1%)	0	0	0	1 (<1%)	4 (1%)
Cystitis noninfective	3 (<1%)	0	0	0	3 (1%)	1 (<1%)	0	0	0	1 (<1%)	4 (1%)
Testicular pain	3 (<1%)	0	0	0	3 (1%)	1 (<1%)	0	0	0	1 (<1%)	4 (1%)
Oropharyngeal pain	2 (<1%)	0	0	0	2 (<1%)	2 (1%)	0	0	0	2 (1%)	4 (1%)
Pruritus	3 (<1%)	0	0	0	3 (1%)	1 (<1%)	0	0	0	1 (<1%)	4 (1%)
Rash	2 (<1%)	0	0	0	2 (<1%)	2 (1%)	0	0	0	2 (1%)	4 (1%)
Hypertension	2 (<1%)	0	0	0	2 (<1%)	1 (<1%)	1 (<1%)	0	0	2 (1%)	4 (1%)
Atrial fibrillation	1 (<1%)	1 (<1%)	0	0	2 (<1%)	1 (<1%)	0	0	0	1 (<1%)	3 (<1%)
Palpitations	1 (<1%)	0	0	0	1 (<1%)	2 (1%)	0	0	0	2 (1%)	3 (<1%)
Vertigo	3 (<1%)	0	0	0	3 (1%)	0	0	0	0	0	3 (<1%)
Dyspepsia	2 (<1%)	0	0	0	2 (<1%)	1 (<1%)	0	0	0	1 (<1%)	3 (<1%)
Frequent bowel movements	2 (<1%)	0	0	0	2 (<1%)	1 (<1%)	0	0	0	1 (<1%)	3 (<1%)
Rectal tenesmus	3 (<1%)	0	0	0	3 (1%)	0	0	0	0	0	3 (<1%)
Paraesthesia	3 (<1%)	0	0	0	3 (1%)	0	0	0	0	0	3 (<1%)
Somnolence	3 (<1%)	0	0	0	3 (1%)	0	0	0	0	0	3 (<1%)
Agitation	2 (<1%)	0	0	0	2 (<1%)	1 (<1%)	0	0	0	1 (<1%)	3 (<1%)
Gynaecomastia	3 (<1%)	0	0	0	3 (1%)	0	0	0	0	0	3 (<1%)
Perineal pain	1 (<1%)	1 (<1%)	0	0	2 (<1%)	1 (<1%)	0	0	0	1 (<1%)	3 (<1%)
Prostatic pain	2 (<1%)	0	0	0	2 (<1%)	1 (<1%)	0	0	0	1 (<1%)	3 (<1%)
Vision blurred	2 (<1%)	0	0	0	2 (<1%)	0	0	0	0	0	2 (<1%)
Defaecation urgency	1 (<1%)	0	0	0	1 (<1%)	1 (<1%)	0	0	0	1 (<1%)	2 (<1%)
Gastrointestinal pain	1 (<1%)	0	0	0	1 (<1%)	1 (<1%)	0	0	0	1 (<1%)	2 (<1%)
Stomatitis	1 (<1%)	0	0	0	1 (<1%)	1 (<1%)	0	0	0	1 (<1%)	2 (<1%)
Thirst	1 (<1%)	0	0	0	1 (<1%)	1 (<1%)	0	0	0	1 (<1%)	2 (<1%)
Bacteraemia	0	1 (<1%)	0	0	1 (<1%)	1 (<1%)	0	0	0	1 (<1%)	2 (<1%)
Sepsis	0	0	2 (<1%))	0	1 (<1%)	0	0	0	0	0	2 (<1%)
Skin infection	1 (<1%)	0	0	0	1 (<1%)	1 (<1%)	0	0	0	1 (<1%)	2 (<1%)
Fall	1 (<1%)	0	0	0	1 (<1%)	0	1 (<1%)	0	0	1 (<1%)	2 (<1%)
Muscle spasms	1 (<1%)	0	0	0	1 (<1%)	1 (<1%)	0	0	0	1 (<1%)	2 (<1%)
Neck pain	1 (<1%)	0	0	0	1 (<1%)	0	1 (<1%)	0	0	1 (<1%)	2 (<1%)
Cerebrovascular accident	0	2 (<1%)	0	0	2 (<1%)	0	0	0	0	0	2 (<1%)
Lethargy	2 (<1%)	0	0	0	2 (<1%)	0	0	0	0	0	2 (<1%)

Presyncope	1 (<1%)	1 (<1%)	0	0	2 (<1%)	0	0	0	0	0	2 (<1%)
Anxiety	2 (<1%)	0	0	0	2 (<1%)	0	0	0	0	0	2 (<1%)
Euphoric mood	2 (<1%)	0	0	0	2 (<1%)	0	0	0	0	0	2 (<1%)
Irritability	2 (<1%)	0	0	0	2 (<1%)	0	0	0	0	0	2 (<1%)
Libido decreased	2 (<1%)	0	0	0	2 (<1%)	0	0	0	0	0	2 (<1%)
Bladder spasm	1 (<1%)	0	0	0	1 (<1%)	1 (<1%)	0	0	0	1 (<1%)	2 (<1%)
Haematospermia	1 (<1%)	0	0	0	1 (<1%)	1 (<1%)	0	0	0	1 (<1%)	2 (<1%)
Prostatitis	2 (<1%)	0	0	0	2 (<1%)	0	0	0	0	0	2 (<1%)
Alopecia	1 (<1%)	0	0	0	1 (<1%)	1 (<1%)	0	0	0	1 (<1%)	2 (<1%)
Dry skin	2 (<1%)	0	0	0	2 (<1%)	0	0	0	0	0	2 (<1%)
Night sweats	2 (<1%)	0	0	0	2 (<1%)	0	0	0	0	0	2 (<1%)
Embolism	0	1 (<1%)	0	0	1 (<1%)	1 (<1%)	0	0	0	1 (<1%)	2 (<1%)
Flushing	2 (<1%)	0	0	0	2 (<1%)	0	0	0	0	0	2 (<1%)
Haematoma	1 (<1%)	0	0	0	1 (<1%)	1 (<1%)	0	0	0	1 (<1%)	2 (<1%)
Acute coronary syndrome	0	0	0	0	0	0	1 (<1%)	0	0	1 (<1%)	1 (<1%)
Angina pectoris	1 (<1%)	0	0	0	1 (<1%)	0	0	0	0	0	1 (<1%)
Myocardial infarction	0	0	0	0	0	0	1 (<1%)	0	0	1 (<1%)	1 (<1%)
Hypoacusis	1 (<1%)	0	0	0	1 (<1%)	0	0	0	0	0	1 (<1%)
Diplopia	1 (<1%)	0	0	0	1 (<1%)	0	0	0	0	0	1 (<1%)
Lacrimation increased	1 (<1%)	0	0	0	1 (<1%)	0	0	0	0	0	1 (<1%)
Uveitis	1 (<1%)	0	0	0	1 (<1%)	0	0	0	0	0	1 (<1%)
Acquired macroglossia	0	0	0	0	0	1 (<1%)	0	0	0	1 (<1%)	1 (<1%)
Anal haemorrhage	0	0	0	0	0	1 (<1%)	0	0	0	1 (<1%)	1 (<1%)
Anorectal discomfort	0	0	0	0	0	1 (<1%)	0	0	0	1 (<1%)	1 (<1%)
Colitis	0	0	0	0	0	0	0	1 (<1%)	0	1 (<1%)	1 (<1%)
Paraesthesia oral	0	0	0	0	0	1 (<1%)	0	0	0	1 (<1%)	1 (<1%)
Periodontal disease	0	0	0	0	0	1 (<1%)	0	0	0	1 (<1%)	1 (<1%)
Tooth loss	1 (<1%)	0	0	0	1 (<1%)	0	0	0	0	0	1 (<1%)
Toothache	1 (<1%)	0	0	0	1 (<1%)	0	0	0	0	0	1 (<1%)
Injection site reaction	1 (<1%)	0	0	0	1 (<1%)	0	0	0	0	0	1 (<1%)
Non-cardiac chest pain	1 (<1%)	0	0	0	1 (<1%)	0	0	0	0	0	1 (<1%)
Hypersensitivity	0	1 (<1%)	0	0	1 (<1%)	0	0	0	0	0	1 (<1%)

Bronchitis	1 (<1%)	0	0	0	1 (<1%)	0	0	0	0	0	1 (<1%)
Enterocolitis infectious	0	0	0	0	0	0	0	1 (<1%)	0	1 (<1%)	1 (<1%)
Epididymitis	1 (<1%)	0	0	0	1 (<1%)	0	0	0	0	0	1 (<1%)
Eye infection	1 (<1%)	0	0	0	1 (<1%)	0	0	0	0	0	1 (<1%)
Gingivitis	0	0	0	0	0	1 (0.4)	0	0	0	1 (<1%)	1 (<1%)
Oral candidiasis	1 (<1%)	0	0	0	1 (<1%)	0	0	0	0	0	1 (<1%)
Orchitis	0	0	0	0	0	1 (<1%)	0	0	0	1 (<1%)	1 (<1%)
Perirectal abscess	1 (<1%)	0	0	0	1 (<1%)	0	0	0	0	0	1 (<1%)
Scrotal infection	0	0	0	0	0	0	1 (<1%)	0	0	1 (<1%)	1 (<1%)
Soft tissue infection	0	1 (<1%)	0	0	1 (<1%)	0	0	0	0	0	1 (<1%)
Tooth abscess	1 (<1%)	0	0	0	1 (<1%)	0	0	0	0	0	1 (<1%)
Urethritis	0	0	0	0	0	1 (<1%)	0	0	0	1 (<1%)	1 (<1%)
Urosepsis	0	1 (<1%)	0	0	1 (<1%)	0	0	0	0	0	1 (<1%)
Contusion	1 (<1%)	0	0	0	1 (<1%)	0	0	0	0	0	1 (<1%)
Fracture	1 (<1%)	0	0	0	1 (<1%)	0	0	0	0	0	1 (<1%)
Radiation skin injury	1 (<1%)	0	0	0	1 (<1%)	0	0	0	0	0	1 (<1%)
Tendon injury	1 (<1%)	0	0	0	1 (<1%)	0	0	0	0	0	1 (<1%)
Blood pressure increased	0	1 (<1%)	0	0	1 (<1%)	0	0	0	0	0	1 (<1%)
Urine output decreased	1 (<1%)	0	0	0	1 (<1%)	0	0	0	0	0	1 (<1%)
Weight decreased	1 (<1%)	0	0	0	1 (<1%)	0	0	0	0	0	1 (<1%)
Weight increased	1 (<1%)	0	0	0	1 (<1%)	0	0	0	0	0	1 (<1%)
Hyponatraemia	0	1 (<1%)	0	0	1 (<1%)	0	0	0	0	0	1 (<1%)
Coccydynia	1 (<1%)	0	0	0	1 (<1%)	0	0	0	0	0	1 (<1%)
Joint lock	0	0	0	0	0	1 (<1%)	0	0	0	1 (<1%)	1 (<1%)
Joint range of motion decreased	1 (<1%)	0	0	0	1 (<1%)	0	0	0	0	0	1 (<1%)
Balance disorder	1 (<1%)	0	0	0	1 (<1%)	0	0	0	0	0	1 (<1%)
Disturbance in attention	1 (<1%)	0	0	0	1 (<1%)	0	0	0	0	0	1 (<1%)
Dysarthria	0	0	0	0	0	1 (<1%)	0	0	0	1 (<1%)	1 (<1%)
Haemorrhage intracranial	0	0	1 (<1%)	0	1 (<1%)	0	0	0	0	0	1 (<1%)
Neuropathy peripheral	0	0	0	0	0	1 (<1%)	0	0	0	1 (<1%)	1 (<1%)
Peripheral motor neuropathy	1 (<1%)	0	0	0	1 (<1%)	0	0	0	0	0	1 (<1%)
Restless legs syndrome	1 (<1%)	0	0	0	1 (<1%)	0	0	0	0	0	1 (<1%)

Tremor	1 (<1%)	0	0	0	1 (<1%)	0	0	0	0	0	1 (<1%)
Device dislocation	0	0	0	0	0	1 (<1%)	0	0	0	1 (<1%)	1 (<1%)
Abnormal dreams	0	0	0	0	0	1 (<1%)	0	0	0	1 (<1%)	1 (<1%)
Mood swings	0	0	0	0	0	1 (<1%)	0	0	0	1 (<1%)	1 (<1%)
Stress	1 (<1%)	0	0	0	1 (<1%)	0	0	0	0	0	1 (<1%)
Suicidal ideation	0	0	0	0	0	0	1 (<1%)	0	0	1 (<1%)	1 (<1%)
Calculus bladder	1 (<1%)	0	0	0	1 (<1%)	0	0	0	0	0	1 (<1%)
Chromaturia	1 (<1%)	0	0	0	1 (<1%)	0	0	0	0	0	1 (<1%)
Dysuria	1 (<1%)	0	0	0	1 (<1%)	0	0	0	0	0	1 (<1%)
Proteinuria	1 (<1%)	0	0	0	1 (<1%)	0	0	0	0	0	1 (<1%)
Renal pain	1 (<1%)	0	0	0	1 (<1%)	0	0	0	0	0	1 (<1%)
Sexual dysfunction	0	0	0	0	0	1 (<1%)	0	0	0	1 (<1%)	1 (<1%)
Dysphonia	1 (<1%)	0	0	0	1 (<1%)	0	0	0	0	0	1 (<1%)
Epistaxis	1 (<1%)	0	0	0	1 (<1%)	0	0	0	0	0	1 (<1%)
Nasal cavity mass	1 (<1%)	0	0	0	1 (<1%)	0	0	0	0	0	1 (<1%)
Upper-airway cough syndrome	1 (<1%)	0	0	0	1 (<1%)	0	0	0	0	0	1 (<1%)
Wheezing	0	0	0	0	0	1 (<1%)	0	0	0	1 (<1%)	1 (<1%)
Dermatitis	0	0	0	0	0	1 (<1%)	0	0	0	1 (<1%)	1 (<1%)
Dermatitis acneiform	0	0	0	0	0	1 (<1%)	0	0	0	1 (<1%)	1 (<1%)
Eczema	1 (<1%)	0	0	0	1 (<1%)	0	0	0	0	0	1 (<1%)
Nail discolouration	0	0	0	0	0	1 (<1%)	0	0	0	1 (<1%)	1 (<1%)
Pain of skin	0	0	0	0	0	1 (<1%)	0	0	0	1 (<1%)	1 (<1%)
Psoriasis	0	0	0	0	0	1 (<1%)	0	0	0	1 (<1%)	1 (<1%)
Skin induration	1 (<1%)	0	0	0	1 (<1%)	0	0	0	0	0	1 (<1%)
Skin ulcer	0	0	0	0	0	1 (<1%)	0	0	0	1 (<1%)	1 (<1%)
Urticaria	0	0	0	0	0	1 (<1%)	0	0	0	1 (<1%)	1 (<1%)
Tooth extraction	0	0	0	0	0	1 (<1%)	0	0	0	1 (<1%)	1 (<1%)
Haematocoele	1 (<1%)	0	0	0	1 (<1%)	0	0	0	0	0	1 (<1%)

Treatment-emergent AE was defined as any AE with onset after the administration of study medication (i.e., the first aglatimagene or placebo injection) until 30 days after the last administration of study treatment, or any event that was present at baseline but worsened in intensity through 30 days after the last administration of study treatment.

No Grade 5 AEs were reported.

MedDRA v27·1 dictionary was used for coding TEAEs. Treatment-emergent AEs were graded using the National Cancer Institute (NCI) Common Terminology Criteria for Adverse Events (CTCAE), version 4·0.

Definitions: AE=adverse event; TEAE=treatment-emergent AE; SAE=serious AE

S10 Part 2. Treatment-Related Treatment-Emergent Adverse Events Grade ≥3 (Safety Population)

AE term	Aglatimagene+ Valacyclovir (N=479)			Placebo+ Valacyclovir (N=232)			Total (N=711)
	Grade 3 N (%)	Grade 4 N (%)	Total G3-G4 N(%)	Grade 3 N (%)	Grade 4 N (%)	Total G3-G4 N (%)	Total N (%)
Acute kidney injury	4 (<1)	1(<1)	**5 (1)**	3(1)	··	**3(1)**	8 (1)
Flu-like symptoms	2 (<1)	··	**2 (<1)**	··	··	**··**	2 (<1)
Fatigue	1(<1)	··	**1(<1)**	1(<1)	··	**1(<1)**	2(<1)
Muscular weakness	1(<1)	··	**1(<1)**	1(<1)	··	**1(<1)**	2 (<1)
Headache	1(<1)	··	**1(<1)**	··	··	**··**	1(<1)
Urinary retention	1(<1)	··	**1(<1)**	··	··	**··**	1(<1)
Perineal pain	1(<1)	··	**1(<1)**	··	··	**··**	1(<1)
Gait disturbance	··	··	**··**	1(<1)	··	**1(<1)**	1(<1)
Syncope	··	··	**··**	1(<1)	··	**1(<1)**	1(<1)
Blood pressure increased	1(<1)	··	**1(<1)**	··	··	··	1(<1)

Treatment-emergent AE was defined as any AE with onset after the administration of study medication (i.e., the first aglatimagene or placebo injection) until 30 days after the last administration of study treatment, or any event that was present at baseline but worsened in intensity through 30 days after the last administration of study treatment.

No Grade 5 AEs were reported.

MedDRA v27·1 dictionary was used for coding TEAEs. Treatment-emergent AEs were graded using the National Cancer Institute (NCI) Common Terminology Criteria for Adverse Events (CTCAE), version 4·0

Definitions: AE=adverse event; TEAE=treatment-emergent AE

S10 Part 3. Treatment Emergent Serious Adverse Events by Preferred Term (Safety Population)

Preferred Term	CAN-2409 + Valacyclovir (N=479) N (%)	Placebo + Valacyclovir (N=232) N (%)	Total (N=711) N (%)
Patients with any SAE	28 (6)	17 (7)	45 (6)
Acute kidney injury	7 (2)	4 (2)	11 (2)
Cerebrovascular accident	2 (<1)	··	2 (<1)
Pneumonia	2 (<1)	1 (<1)	3 (<1)
Pyrexia	2 (<1)	··	2 (<1)
Sepsis	2 (<1)	··	2 (<1)
Urinary retention	2 (<1)	··	2 (<1)
Urinary tract infection	2 (<1)	1 (<1)	3 (<1)
Atrial fibrillation	1 (<1)	··	1 (<1)
Constipation	1 (<1)	··	1 (<1)
Diarrhoea	1 (<1)	··	1 (<1)
Dyspnoea	1 (<1)	··	1 (<1)
Embolism	1 (<1)	··	1 (<1)
Haemorrhage intracranial	1 (<1)	··	1 (<1)
Hyperglycaemia	1 (<1)	··	1 (<1)
Flu-like symptoms	1 (<1)	··	1 (<1)
Soft tissue infection	1 (<1)	··	1 (<1)
Urosepsis	1 (<1)	··	1 (<1)
Blood creatinine increased	··	1 (<1)	1 (<1)
Colitis	··	1 (<1)	1 (<1)
Enterocolitis infectious	··	1 (<1)	1 (<1)
Hypertension	··	1 (<1)	1 (<1)

Hypotension	..	1 (<1)	1 (<1)
Myocardial infarction	..	1 (<1)	1 (<1)
Neck pain	..	1 (<1)	1 (<1)
Rash	..	1 (<1)	1 (<1)
Scrotal infection	..	1 (<1)	1 (<1)
Suicidal ideation	..	1 (<1)	1 (<1)
Tooth extraction	..	1 (<1)	1 (<1)

Treatment-emergent AE was defined as any AE with onset after the administration of study medication (i.e., the first aglatimagene or placebo injection) until 30 days after the last administration of study treatment, or any event that was present at baseline but worsened in intensity through 30 days after the last administration of study treatment.

No Grade 5 AEs were reported.

MedDRA v27·1 dictionary was used for coding TEAEs. Treatment-emergent AEs were graded using the National Cancer Institute (NCI) Common Terminology Criteria for Adverse Events (CTCAE), version 4·0]

Definitions: SAE=serious AE

S10 Part 4. Treatment-related Serious Adverse Events by Preferred Term (Safety Population)

Preferred Term	CAN-2409 + Valacyclovir (N=479) N (%)	Placebo + Valacyclovir (N=232) N (%)	Total (N=711) N (%)
Patients with any treatment-related SAE	8 (2)	5 (2)	13 (2)
Acute kidney injury	4 (1)	4 (2)	8 (1)
Pyrexia	2 (<1)	··	2 (<1)
Flu-like symptoms	1 (<1)	··	1 (<1)
Urinary retention	1 (<1)	··	1 (<1)
Blood creatinine increased	··	1 (<1)	1 (<1)
Rash	··	1 (<1)	1 (<1)

Treatment-emergent AE was defined as any AE with onset after the administration of study medication (i.e., the first aglatimagene or placebo injection) until 30 days after the last administration of study treatment, or any event that was present at baseline but worsened in intensity through 30 days after the last administration of study treatment.

MedDRA v27·1 dictionary was used for coding TEAEs. Treatment-emergent AEs were graded using the National Cancer Institute (NCI) Common Terminology Criteria for Adverse Events (CTCAE), version 4·0[22]

Definitions: SAE=serious AE

S11. Quality-of-Life (QoL) results: Change from baseline in EPIC-26 scores by treatment group.

Figure S8-Part 1: Sexual Score

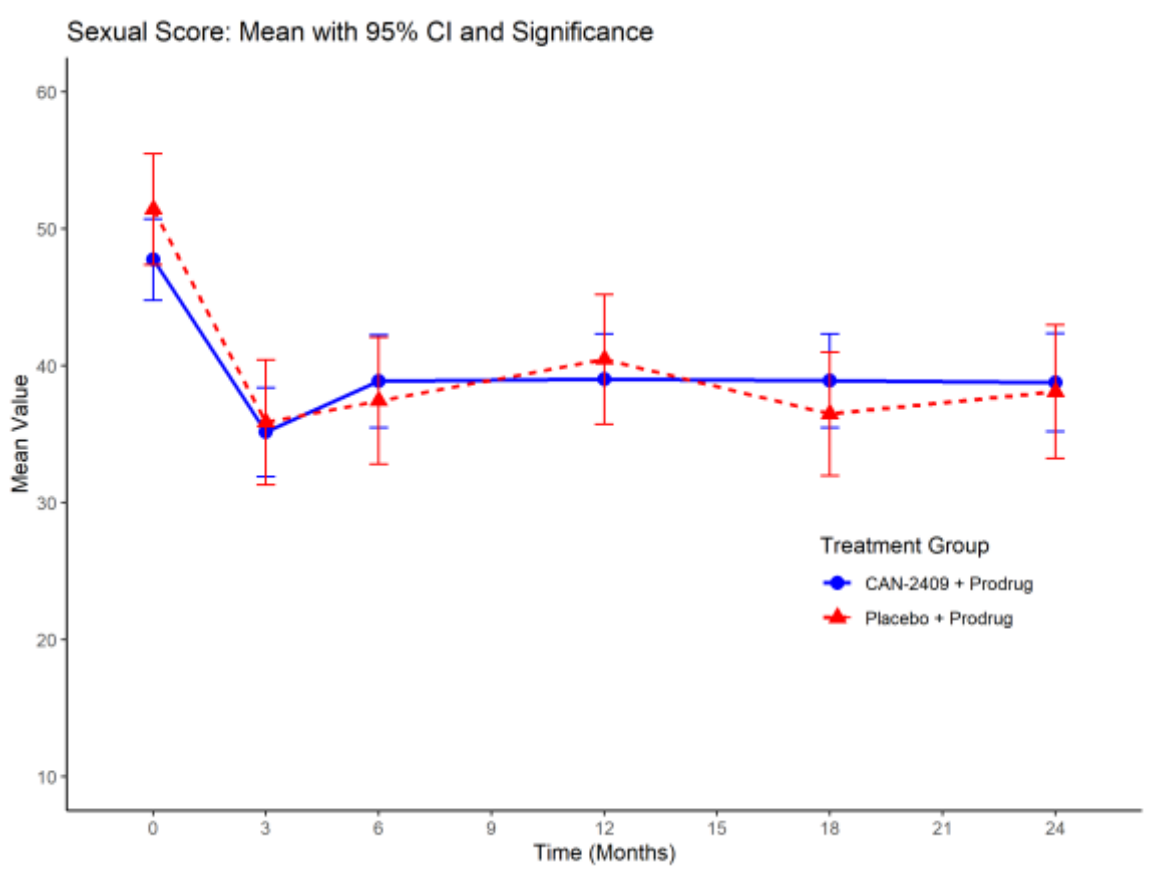

Figure S8-Part 2: Bowel Score

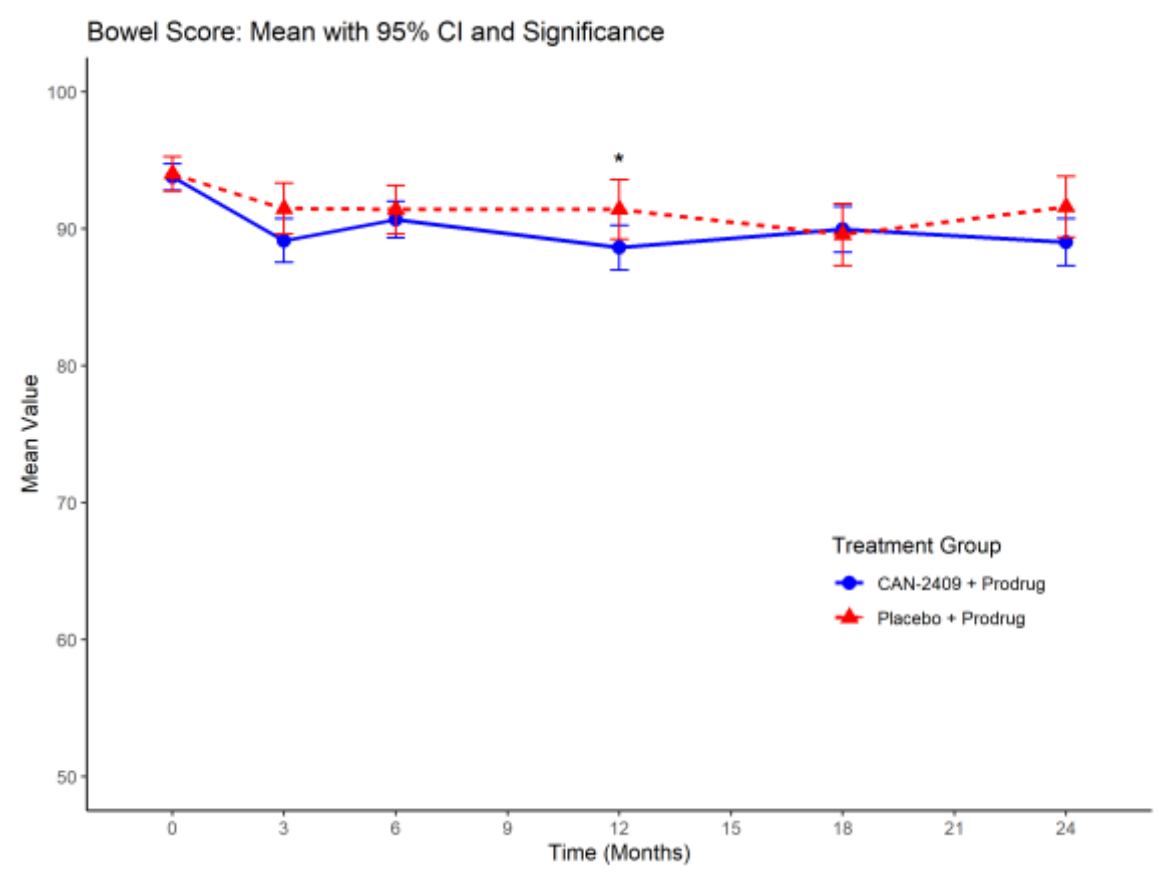

Figure S8-Part 3: Urinary Incontinence Score

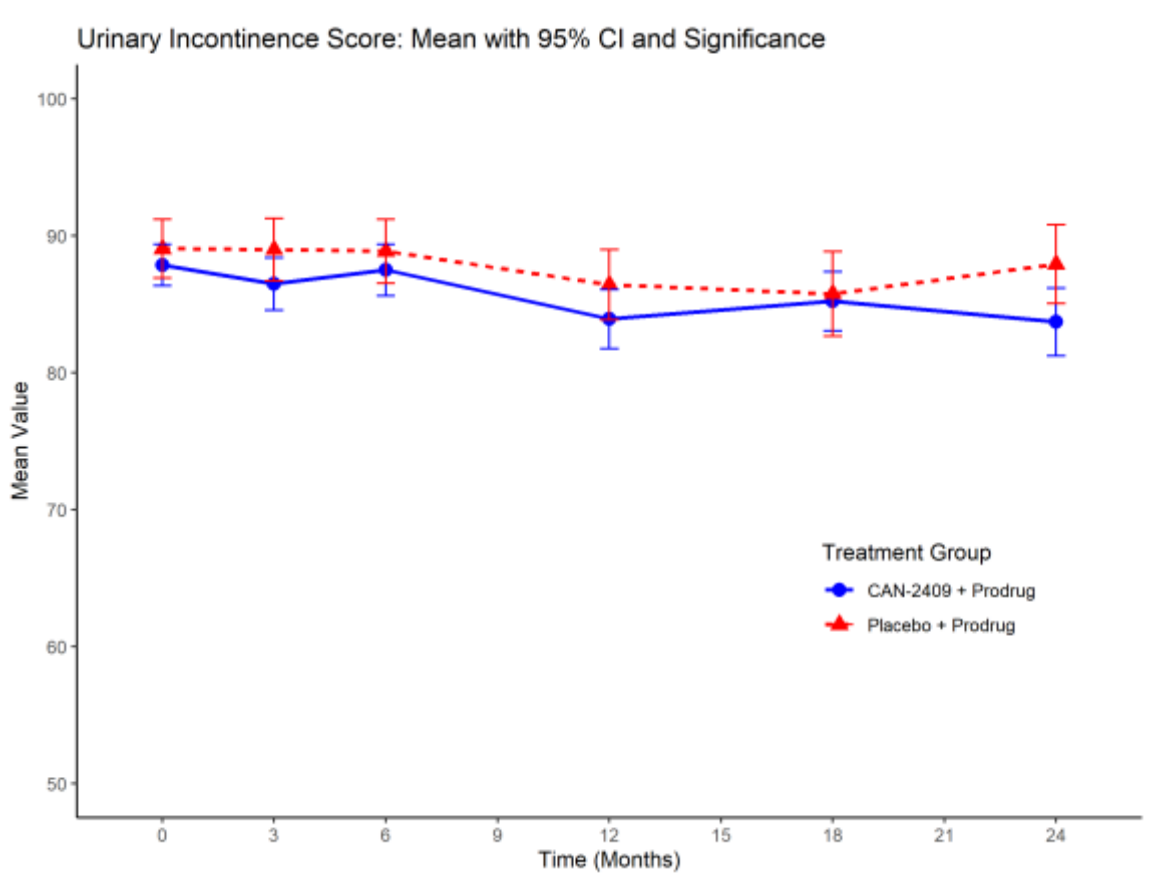

Figure S8-Part 4: Hormonal Score

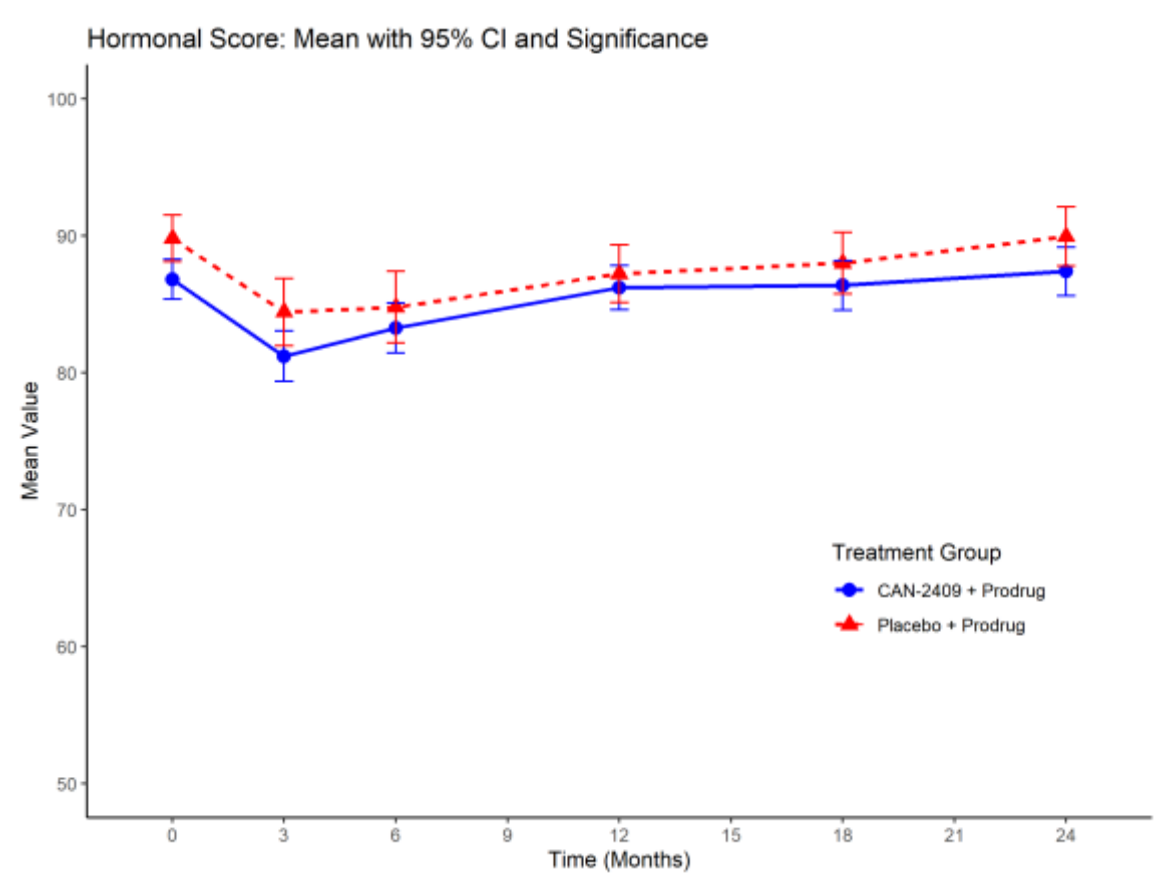

Figure S9-Part 5: Urinary Irritative Score

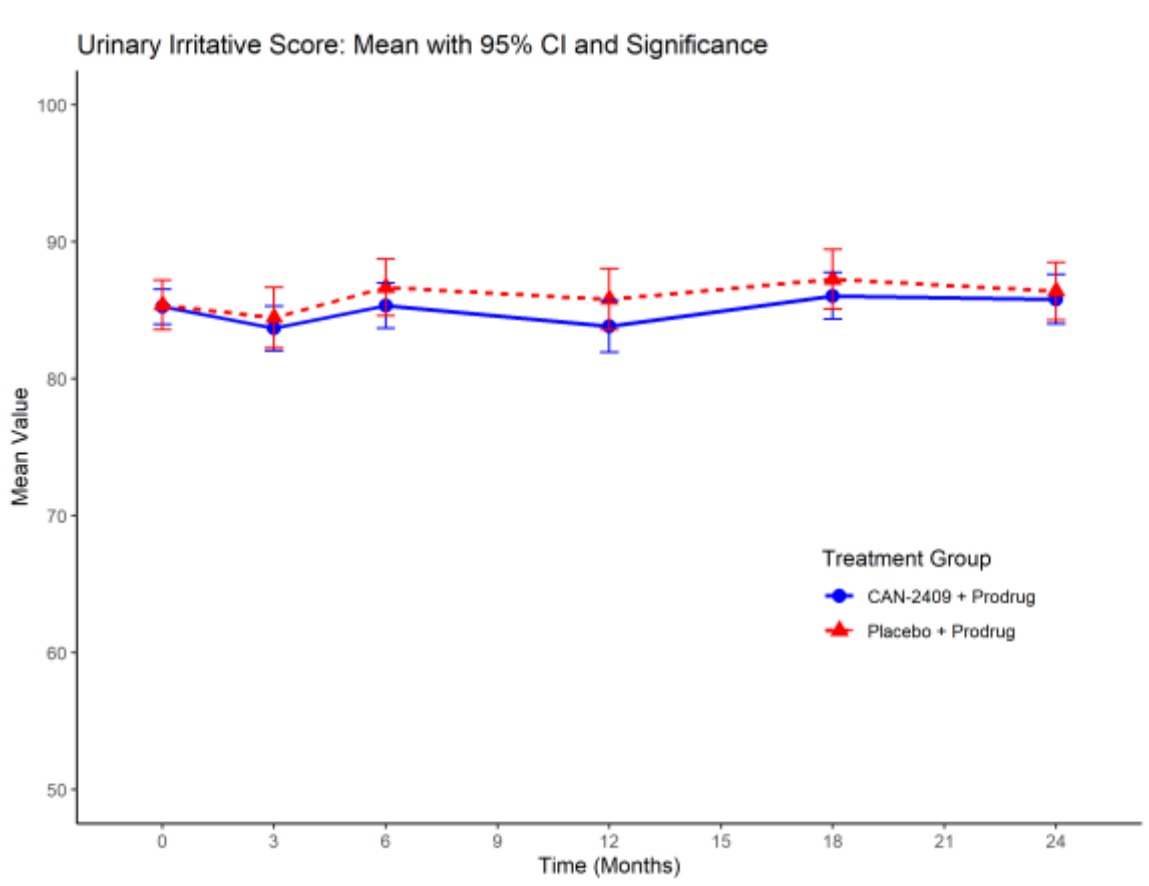

Abbreviations: CI=confidence interval; prodrug=valacyclovir.

The mixed-effects model for repeated measures (MMRM) included treatment, visit (as categorical), treatment by visit, baseline score and stratification factors as fixed effects, and patients being the random effect, was used to estimate changes in QoL score over time. P-values of comparing LS-Mean estimates from two treatment arms was provided from MMRM. Missing scores were not imputed using the MMRM approach assuming an unstructured variance-covariance matrix for the repeated measures.

INVESTIGATIONAL PROTOCOL

A RANDOMIZED CONTROLLED TRIAL OF PROSTATAK® AS ADJUVANT TO UP-FRONT RADIATION THERAPY FOR LOCALIZED PROSTATE CANCER

Protocol No. PrTK03
Protocol Version: February 7, 2019

Sponsor:
Advantagene, Inc.
440 Lexington St.
Auburndale, MA 02466
Off: 617-916-5445
FAX: 617-916-5444

Confidentiality Statement

The information contained herein is confidential and is the proprietary property of Advantagene, Inc. Any unauthorized use or disclosure of such information without the prior authorization of Advantagene, Inc. is expressly prohibited.

Figure 1: Protocol Schema

PROTOCOL SCHEMA

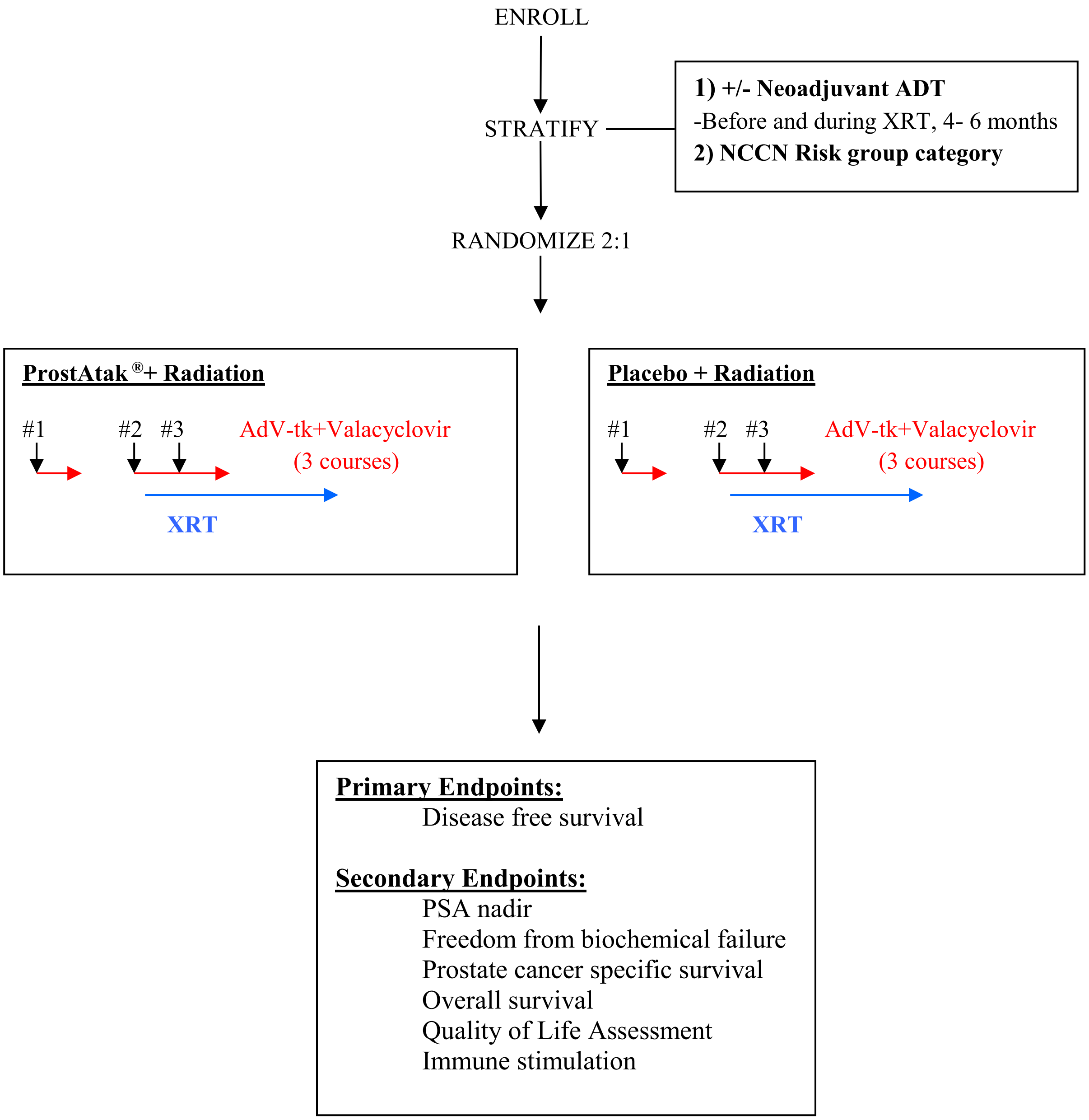

PrTK03 Time and Events Table

Table 1: Time and Events

	Baseline	Course #1	Course #2	Course #3	3 mo[6]	6 mo[6]	12 mo[6]	18 mo[6]	24 mo[6]	q6mo thru year 5 then yearly
Patient Registration	X									
AdV-tk or Placebo Injection		X[1]	X[2]	X[3]						
Valacyclovir pills (x 14 days per course)		X	X	X						
Radiation (XRT)	Begins 0-3 days after injection #2. See protocol section 6.3.									
ADT Therapy	Optional and must be decided at time of enrollment. See protocol section 6.4.									
Clinical Assessment	X[4]	X[5]	X[5]	X[5]	X	X	X	X	X	X
Laboratory Studies (CBC, LFTs, creatinine, calculated creatinine clearance)	X[4]	X[5]	X[5]	X[5]	Repeated if abnormal until return to baseline.					
PSA and DRE	X[4]				X	X	X	X	X	X
Prostate Biopsy	X[4]								**X**	
QOL (patient completes)	X[4]				X	X	X	X	X	
Optional blood collection for research studies	X[4]	X	X	X	X		X			
Case Report Forms/Data Collection	X	X	X	X	X	X	X	X	X	X
1	Injection #1 is at least 2 weeks prior to initiation of XRT (to allow 14-day valacyclovir course completion) and no more than 8 weeks prior to beginning XRT. Valacyclovir starts the day after the injection and continues for 14 days. Subsequent injections can be given on the last day of valacyclovir for the previous course.									
2	Injection #2 is on the day of or up to 3 days prior to beginning XRT and at least 2 weeks after injection #1 (to allow 14-day valacyclovir course completion). Injection #2 may be on the last day of valacyclovir for course 1.									
3	Injection #3 is 2-3 weeks after injection #2 (to allow 14-day valacyclovir course completion). Injection #3 may be on the last day of valacyclovir for course 2.									
4	Baseline evaluations as per protocol section 4 to be done within 4 weeks prior to the 1st injection except for PSA, which is within 6 months and biopsy, which is within 12 months prior to enrollment.									
5	Clinical assessment and lab studies for each course to be done 1-2 weeks after each injection; for course 1 and 2 must be done prior to subsequent injection with recalculation of creatinine clearance if creatinine abnormal.									
6	Follow up time is relative to completion of radiation.									

TABLE OF CONTENTS

1.0 BACKGROUND AND RATIONALE ... 8
2.0 OBJECTIVES ... 23
3.0 PATIENT SELECTION ... 23
4.0 PRETREATMENT EVALUATION ... 24
5.0 REGISTRATION PROCEDURES ... 25
6.0 TREATMENT PLAN ... 25
7.0 PATIENT ASSESMENT ... 29
8.0 STUDY AGENTS- Description and Potential Risks ... 31
9.0 STATISTICS ... 33
10.0 ETHICAL AND REGULATORY CONSIDERATIONS ... 37
11.0 BIBLIOGRAPHY ... 39

TABLE OF TABLES

Table 1: Time and Events ... 3
Table 2: Summary of completed prostate cancer trials with AdV-tk ... 17
Table 3: PSA Nadir Comparison ... 18
Table 4: 2-Year Biopsy Results in Multiple Contemporaneous Radiation Studies ... 19
Table 5: Freedom from Failure Comparison to Contemporary Studies* ... 21
Table 6: Study Details for Table 5 ... 21
Table 7: NCCN Risk Criteria ... 23
Table 8: Valacyclovir Dosage for Calculated Creatinine Clearance ... 26
Table 9: Safety Reporting ... 38

TABLE OF FIGURES

Figure 1: Protocol Schema ... 2
Figure 2: GMCI™ mechanism for induction of systemic anti-tumor immune response from local intratumoral administration of ProstAtak® ... 11
Figure 3: Local delivery of ProstAtak® had a significant effect on decreasing systemic metastatic tumor load ... 14
Figure 4: Objective clinical responses from Phase I trial in recurrent radiation-failure prostate cancer ... 15
Figure 5: Kaplan-Meier Freedom from Failure (FFF) Curves Using Nadir+2 Definition ... 20
Figure 6: Kaplan-Meier Overall Survival for ProstAtak® Phase 2a Study ... 20

Abbreviations

ACV	Acyclovir
ADT	Androgen Deprivation Therapy
AdV	Adenovirus
AdV-βgal	AdV-β Galactocidase
AdV-tk	Aglatimagene besadenovec
AE	Adverse Event
AJCC	American Joint Committee on Cancer
Alk. Phos.	Alkaline Phosphatase
ALT	Alanine transaminase
APC	Antigen presenting cell
AST	Aspartate aminotransferase
ASTRO	American Society for Radiation Oncology
CBC	Complete blood count
CD4	Cluster of differentiation 4
CD8	Cluster of differentiation 8
cGMP	Current Good Manufacturing Practice
CNS	Central nervous system
Concurrent tx	Concurrent Treatment
Cr	Creatinine
CRF	Case Report Form
CT	Computed tomography
CTCAE	Common Toxicity Criteria for Adverse events
CTV	Clinical Target Volume
DFS	Disease free survival
DNA	Deoxyribonucleic acid
DRE	Digital Rectal Exam
DSMB	Data Safety Monitoring Board
DVH	Dose-volume histogram
EBRT	External beam radiation therapy
EC	Eligibility Checklist
ECOG	Eastern Cooperative Oncology Group
EPIC	Expanded Prostate Cancer Index Composite
FDA	Food and Drug Administration
FFF	Freedom from failure
GCP	Good Clinical Practice
GCV	Ganciclovir
GI	Gastrointestinal
GMCI™	Gene Mediated Cytotoxic Immunotherapy
GS	Gleason Score
GTV	Gross Tumor Volume
GU	Genitourinary

Gy	Gray. The unit of absorbed radiation dose
HIV	Human immunodeficiency virus
HSV-tk	Herpes simplex virus thymidine kinase gene
HUS	Hemolytic uremic syndrome
IBC	Institutional Biosafety Committee
IL	Interleukin
IMRT	Intensity modulated radiation therapy
IRB	Institutional Review Board
ITT	Intent to treat
Kg	Kilogram
kV-CT	Deformable registration of the planning image
LFT	Liver function tests
LHRH	Luteinizing hormone-releasing hormone
MDACC	MD Anderson Cancer Center
mg	Milligram
mg/dL	Milligrams per deciliter
MHC	Major histocompatibility complex
ml	Milliliter
mm3	Cubic millimetre
MRI	Magnetic resonance imaging
MSKCC	Memorial Sloan Kettering Cancer Center
MV	Megavoltage
MV-CT	Megavoltage computed tomography
NCCN	National Comprehensive Cancer Network
NCI	National Cancer Institute
ng	Nanogram
NIH	National Institutes of Health
OBA	Office of Biotechnology Activities
pCR	Pathological complete response
Post-tx	Post-treatment
PR	Patient Registration
Pre-tx	Pre-treatment
PS	Prodrug Storage
PSA	Prostate-specific antigen
PSA-DT	Prostate specific antigen doubling time
PSAR	Prostate specific antigen reduction
Pts	Patients
PTV	Planning Target Volume
QOL	Quality of Life
RP	Radical prostatectomy
RT	Radiation therapy
RTOG	Radiation Therapy Oncology Group

SAS	Statistical Analysis System
SEER	Surveillance, Epidemiology, and End Results
SGOT (AST)	Serum glutamic-oxaloacetic transaminase
SOC	Standard of care
SOP	Standard Operating Procedure
T Stage	Tumor stage
TAA	Tumor associated antigens
TK	Herpes simplex virus thymidine kinase gene
TNM staging	Tumor (T), nodes (N), and metastases (M)
TRUS	Transrectal ultrasound
TTP	Thrombotic thrombocytopenic purpura
TURP	Transurethral resection of the prostate
vp	Vector particles
VS	Vector Storage
WBC	White blood cell
XRT	Radiotherapy

1.0 BACKGROUND AND RATIONALE

1.1 Hypothesis

Adjuvant therapy with ProstAtak® for newly diagnosed prostate cancer will improve the clinical outcome from standard radiation therapy.

1.2 Introduction

In the United States there are approximately 200,000 new annual diagnoses of prostate cancer. Current therapies provide an excellent 5-year survival prognosis, yet prostate cancer is still the second leading cause of cancer death in men in the U.S. (American Cancer Society Facts and Figures 2008). Recurrence within 10 years occurs in 30-50% of patients, most occurring within 5 years of treatment (Shipley *et al.,* 1999, D'Amico *et al.,* 2003, Han *et al.,* 2003). In the U.S., this translates into an annual incidence of prostate cancer recurrence of 60,000-100,000 and a prevalence of around 2.1 million in 2005 (SEER Cancer Statistics Review, 1975-2005). Prostate cancer recurrence is a growing problem in the US. It is normally treated by surgical or pharmacologic castration (androgen deprivation therapy or ADT), which has a significant negative impact on quality of life (QOL) (Penson *et al.,* 2003). As a consequence of better diagnostic tools and surveillance, the population of men affected by prostate cancer is shifting to younger, healthier men who may be most greatly impacted by recurrence and its treatment effects (Moul 2000). The costs to society from treatment costs, lost productivity and compromised quality of life due to recurrence are in the billions of dollars. Drugs that could decrease prostate cancer recurrence without diminishing current success or increasing the toxicity from standard therapies would be of great significance.

ProstAtak® is a biologic therapeutic designed to prevent prostate cancer recurrence from tumor cells not eliminated by standard therapy. It is not designed as a de-bulking agent to treat large primary or metastatic tumor masses. Thus, it is intended to work in conjunction with the standard of care used for de-bulking, to improve local control, stimulate systemic anti-cancer immune surveillance, decrease recurrence and improve long-term disease-free survival.

1.3 Treatment of Prostate Cancer

The majority of newly diagnosed prostate cancers are considered localized disease. Treatment options for local disease include radiation therapy (RT) and radical prostatectomy (RP). Radiation therapy may be delivered as external beam radiation therapy (EBRT), brachytherapy (radioactive seed implantation) or a combination of both. For patients with higher risk prostate cancer, the addition of androgen deprivation therapy (ADT) to primary radiation has been demonstrated to improve disease free survival and overall survival (Pilepich *et al.,* 2001, Bolla *et al.,* 2002, Hanks *et al.,* 2003, D'Amico *et al.,* 2004). However, even with ADT, 20-50% of these patients suffered recurrence within 5 years and this rate is expected to rise with longer term follow up, as recurrences are known to continue 5-15 years after treatment (Roach *et al.,* 2000, Han *et al.* 2003).

Patients with localized prostate cancer are divided into prognostic groups based on pretreatment factors, which predict future risk of suffering morbidity or mortality as a result of recurrence. The three main factors are pretreatment PSA, combined Gleason score (GS) and clinical stage (T stage). Various commonly used systems divide patients into low, intermediate and high risk categories using these three factors, with slight variations in the definitions used (D'Amico *et al.,*

1998, Roach *et al.,* 2000, Zelefsky *et al.,* 2002, Scherr *et al.,* 2003). An example from the National Comprehensive Cancer Network (NCCN) guidelines (Scherr *et al.* 2003) (www.nccn.org) is as follows:

Low risk:	PSA<10ng/ml, GS ≤6 and T1-T2a*
Intermediate risk:	At least 1 of following: PSA 10-20, GS=7 or T2b-2c*
High risk:	At least 1 of following: PSA>20, GS>7 or T3a
Locally advanced/very high:	T3b-T4

*2002 AJCC TNM classification: T2a: half of 1 lobe, T2b: 1 lobe, T2c: both lobes of prostate

Efforts to improve the outcome of primary therapy in prostate cancer have mostly focused on manipulating the current standard therapies, by increasing dose or approach, while minimizing the side effects. For radiation therapy, advances have been made through better delivery methods that allow increasing the radiation dose without a significant increase in side effects and by the use of adjuvant androgen deprivation therapy. Multiple groups have evaluated radiation dose escalation leading to the employment of higher doses in many treatment centers (Shipley *et al.,* 1995, Zelefsky *et al.,* 2001, Zietman 2002, Kuban *et al.,* 2003). For example, a study of 1325 patients at 9 institutions, with a median follow up of 5.7 years, revealed an improvement in freedom from failure (FFF), from 68.4% at <72Gy to 75.6% for ≥72 Gy (Kupelian *et al.,* 2005). Separated by risk groups, for the patients treated with ≥72Gy, the FFF rate was 79% for low risk, 72% for intermediate risk and 46% for high risk.

Similarly, a randomized trial conducted at M.D. Anderson compared 70Gy to 78Gy in 301 patients with stage T1-T3 disease (Pollack *et al.,* 2002, Kuban *et al.,* 2008). The 78Gy arm had a significant improvement in freedom from biochemical or clinical failure after median follow ups of 5 and 8.7 years. Although overall survival was not considered statistically different, twice as many patients died from prostate cancer or had recurrent disease in the 70Gy arm compared to the 78Gy arm. Subset analysis revealed that 78Gy had the greatest benefit to patients with a pre-PSA >10 ng/ml however, intermediate-risk patients, as a whole, had no statistically significant difference with 78Gy versus 70Gy. Other studies have found a lack of benefit from dose escalation in the extremes, very low risk patients and in highest risk patients (PSA≥20 ng/ml and T3-T4) (Hurwitz *et al.,* 2002, Pollack *et al.,* 2004).

Regardless of the patient risk stratification, the potential benefits of higher radiation doses are tempered by the risk of increased toxicity. For example, in the M.D. Anderson study, gastrointestinal (GI) toxicity occurred twice as often (26% vs. 13%) in the high dose patients (Kuban *et al.* 2008). This is particularly pertinent as patients with longer life expectancies are treated. A large Dutch multicenter randomized study with 664 evaluable patients that compared 68 Gy to 78 Gy found an 11% improvement in the 7-year freedom from failure (FFF) rate, from 45% to 56% (Peeters *et al.,* 2006, Al-Mamgani *et al.,* 2008). However, at the same time, there was also a significant increase in late grade-2 or greater GI toxicity, from 25% in the low dose arm to 35% in the high dose arm (Al-Mamgani *et al.,* 2008). Similarly, in a large British study, which randomized 843 patients to 64 Gy vs 74 Gy, with both arms receiving 3-6 months of

ADT, the high dose arm had improved biochemical progression free survival (71% vs 60%), but also a concomitant increased late GI toxicity (33% vs 24%) (Dearnaley *et al.,* 2007).

Newer radiation therapy techniques, such as intensity modulated radiation therapy (IMRT), have made it possible to deliver higher doses while limiting the risk of increased toxicity. In the Dutch study, the incidence of acute GI toxicity was significantly lower in patients who received IMRT compared to conventional larger field delivery. Similarly the incidences of acute genitourinary (GU) and late GI and GU toxicities were also lower, though these differences were not statistically significant (Al-Mamgani *et al.* 2008). An alternative radiation therapy approach using proton beams has the potential to reduce exposure of structures outside the tumor target even further. However, for prostate cancer, this approach has not yet proven effective in improving the outcome. In a radiation dose comparison study, patients received 50.4Gy via conventional photon therapy with a randomization to a boost of 19.8GyE or 28.8GyE via proton therapy to a total dose of 70.2 or 79.2GyE (Zietman *et al.,* 2005). The higher dose arm had improved FFF but at the price of increased late grade-2 rectal morbidity (Zietman *et al.* 2005, Nguyen *et al.,* 2008). Brachytherapy, another method of delivering radiation, has demonstrated similar efficacy to IMRT but with increased acute and late GU toxicity and increased late GI toxicity (Eade *et al.,* 2008).

Even at higher radiation doses, the recurrence rate is still substantial. Results from a Memorial Sloan-Kettering Cancer Center study using an IMRT dose of 81Gy, with a median follow up of 7 years for 561 patients, revealed PSA relapse-free survival rates of 89% for favorable, 78% for intermediate and 67% for unfavorable risk groups (Zelefsky *et al.,* 2006). Even with high dose radiation, more than 20% of intermediate risk and more than 30% of high-risk patients still experienced recurrence.

In addition to dose increases, the addition of androgen deprivation therapy to radiation therapy has also been evaluated. In higher risk prostate cancer, adjuvant ADT has yielded improved outcomes in multiple studies (Bolla *et al.* 2002, D'Amico *et al.* 2004). Three randomized RTOG studies (RTOG 8531, 8610 and 9202), using different ADT regimens, have also demonstrated improvements in DFS. In RTOG 9202, longer term ADT yielded better DFS but with increased toxicity (Lawton *et al.,* 2001, Pilepich *et al.* 2001, Hanks *et al.* 2003). More recently, several studies have raised concern about toxicity of ADT outweighing the benefit especially in men with lower risk of death from prostate cancer and in men with moderate to severe co-morbidities of which most common was cardiovascular disease (D'Amico *et al.,* 2008). Another analysis demonstrated increased recurrence in patients who received less than the prescribed 6 months of combined ADT (LHRH agonist + anti-androgen), which was due to discontinuation of anti-androgen secondary to toxicity (D'Amico *et al.,* 2008).

Based on these studies, currently most high-risk patients are treated with ADT in combination with radiation therapy, although the appropriate duration of ADT is still debated. The benefit of adjuvant ADT for intermediate risk disease is still unclear. Nonetheless, most clinicians are treating these patients with short term ADT for 4-6 months starting 2 months before and then concomitant with EBRT (NCCN guidelines for Prostate Cancer 2009 at www.nccn.org: (Nichol *et al.,* 2005).

Despite the use of optimized combined modality therapy for prostate cancer, the rate of recurrence and the incidence of toxicity is still substantial, highlighting the need for continued exploration of additional therapies.

1.4 ProstAtak®

ProstAtak® is an adenoviral vector (AdV-tk), which expresses the herpes simplex virus thymidine kinase gene (HSV-tk or TK), that is delivered intraprostatically, followed by anti-herpetic oral prodrug administration and combined with standard prostate cancer therapies. ProstAtak® (AdV-tk + prodrug) provides characteristics to stimulate a potent tumor vaccine effect through Gene Mediated Cytotoxic Immunotherapy (GMCI™). The proposed mechanism of GMCI™ involves the following TK-specific cytotoxic and immuno-stimulatory properties (Figure 2): (1) TK-mediated necrotic and apoptotic tumor cell killing at the site of injection, which releases tumor associated antigens (TAA). (2) The virion and radiation or surgery induce acute inflammation, which attracts MHC-class II+ professional antigen presenting cells, leading to uptake and presentation of TK-released tumor associated antigens. Simultanously, the virion and TK-mediated cell death, through necrosis and apoptosis, generate "danger signals" that stimulate APC maturation and consequently efficient TAA presentation (3) Effector T cell stimulation is potentiated by a superantigen-like effect of the TK protein, which results in production of cytokines, such as IL-2 and IL-12, with consequent T-cell expansion for a systemic effect.

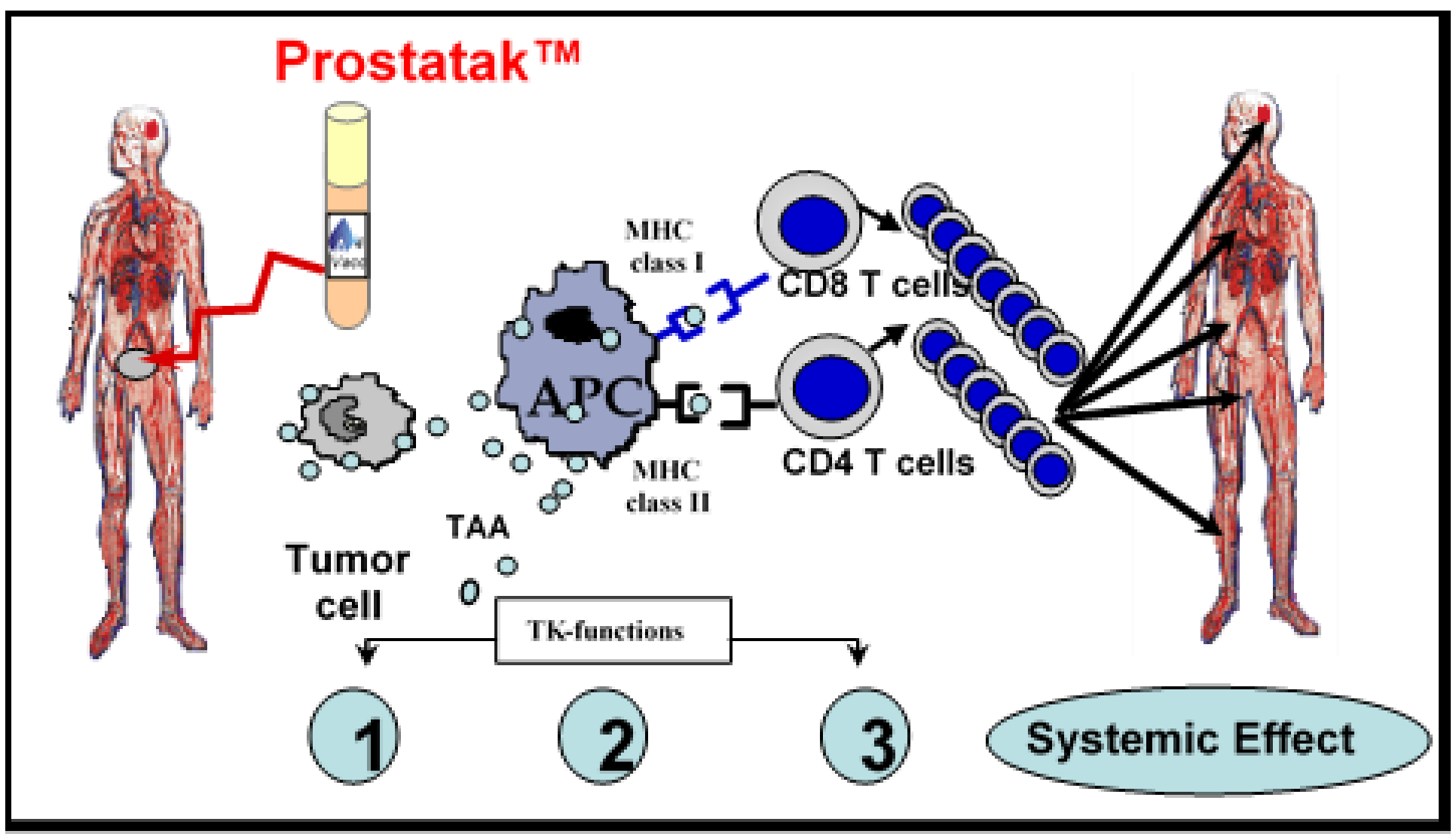

Figure 2: GMCI™ mechanism for induction of systemic anti-tumor immune response from local intratumoral administration of ProstAtak®

The cytotoxic effect of TK is mediated by phosphorylation of the anti-herpetic prodrug such as ganciclovir (GCV), acyclovir (ACV) or valacyclovir, which generates nucleotide analogs that lead to termination of DNA replication and cell death (Fyfe *et al.,* 1978, Eastham *et al.,* 1996). The prodrug is administered systemically but cytotoxicity is localized to the tumor due to the local administration of AdV-tk. Systemic anti-tumor effects are mediated by the anti-tumor immune response which is elicited by the immunostimulatory mileau created in the tumor (Cavallo *et al.,* 1997, Agard *et al.,* 2001, Chhikara *et al.,* 2001, Vlachaki *et al.,* 2001, Teh *et al.,* 2002, Satoh *et al.,* 2004). Thus, the approach generates a patient-specific tumor vaccine with a non-patient-specific drug.
As described below (and in the Investigator's Brochure), extensive data support the safety and potential efficacy of AdV-tk + prodrug in prostate cancer and other tumor types, including multiple Phase I and Phase II clinical studies (Ayala *et al.,* 2000, Trask *et al.,* 2000, Miles *et al.,*

2001, Immonen *et al.,* 2004, Teh *et al.,* 2004) . As a monotherapy, AdV-tk + prodrug demonstrated objective clinical responses in Phase I studies in recurrent prostate cancer (Herman *et al.,* 1999, Ayala *et al.* 2000, Miles *et al.* 2001). Combining AdV-tk + prodrug with standard therapies including radiation and surgery has shown improved anti-tumor effects, including systemic anti-tumor immunity, in preclinical tumor models and Phase I and II clinical trials (Chhikara *et al.* 2001, Sukin *et al.,* 2001). Immune stimulation by AdV-tk + prodrug treatment has been observed in human clinical trials.

1.4.1 Prodrug Selection

Valacyclovir has been evaluated and found to be effective as a prodrug for HSV-tk based anti-cancer studies (Hasenburg *et al.,* 1999, Hasenburg *et al.,* 2000, Teh *et al.,* 2001). Valacyclovir is an orally administered, licensed anti-herpetic medication which is almost completely metabolized in the liver to acyclovir (ACV), achieving similar serum concentrations as intravenous ACV (Weller *et al.,* 1993). In comparison to early trials, in which slow-drip ganciclovir (GCV) was administered intravenously, over one hour twice per day for 14 days (Herman *et al.* 1999, Trask *et al.* 2000), valacyclovir, as an oral prodrug, has significantly decreased the morbidity to patients. At clinically relevant concentrations, ACV and GCV have been shown to be equally effective at killing HSV-tk expressing cells (Tong *et al.,* 1998). As a prodrug for HSV-tk based anti-cancer studies, at the same concentrations ACV has been shown to be less potent than GCV. However, since ACV is less toxic (Shewach *et al.,* 1994) it allows relatively higher doses to be used with equivalent efficacy (Weller *et al.* 1993). Valacyclovir has been used as the prodrug for AdV-tk + prodrug therapy in more than 100 patients with 200 courses of valacyclovir delivered to date.

1.4.2 Vector Selection

Adenoviral vectors have been used most frequently for intratumoral delivery of HSV-tk and other therapeutic genes because of high tumor transduction efficiency, transient expression, non-integration and ease of production. For GMCI™, short duration of expression is sufficient and preferable. The immune response and inflammation induced by adenoviral vectors not only limits expression duration but also serves as an adjuvant for inducing anti-tumor immunity. However, very high doses of adenoviruses may cause significant toxicity if the vector is delivered systemically or to a location where inflammation interferes with organ function. Such conditions likely contributed to the death of a patient on a gene therapy trial for a genetic liver enzyme deficiency who received $3x10^{13}$ vector particles (vp) of an adenoviral vector injected directly into the hepatic artery (Schnell *et al.,* 2001, Raper *et al.,* 2002). In contrast to systemic delivery for a genetic disease, for application with intra-tumor delivery, the localized inflammatory and immune response augment the potential for an anti-tumor response without adding significant toxicity. Therefore, an adenoviral vector was chosen for the GMCI™ technology. As described below (and in the Investigator's Brochure), this approach has been safely used in multiple clinical trials with over 300 patient doses delivered to more than 150 patients.

1.4.3 Preclinical Studies of AdV-tk Alone and In Combination With Radiation

1.4.3.1 Local and Systemic Anti-tumor Effects

Numerous studies in animal models indicate that treatment with AdV-tk + prodrug leads to local tumor response, increased survival and protection against metastases and tumor rechallenge. For example, in a murine prostate tumor model, significant tumor growth delay and increased

survival was observed with AdV-tk and prodrug compared to groups that did not receive prodrug or were treated with an AdV-βgal control vector (Eastham *et al.* 1996). In an orthotopic prostate model, local tumor treatment with AdV-tk + systemic prodrug lead to a significant reduction in spontaneous metastases, increased survival and protection against tumor rechallenge (Hall *et al.*, 1998). Treated tumors had a marked infiltration of leukocytes not seen in control tumors. Other animal studies demonstrated infiltration of treated tumors with macrophages and T cells as well as Th1 cytokine expression (Barba *et al.*, 1994, Vile *et al.*, 1997). The approach has also been shown to induce cytotoxic T lymphocyte responses and is more effective in immunocompetent mice than in immunodeficient mice (Vile *et al.*, 1994, Gagandeep *et al.*, 1996, Hall *et al.* 1998). The data strongly suggest the systemic anti-tumor effect of AdV-tk is immune mediated.

1.4.3.2 Cooperative Effects with Radiation Therapy

The rationale for combining AdV-tk + prodrug with radiation is based on the mechanism and effects of both approaches. AdV-tk + prodrug generates nucleotide analogs that interfere with DNA synthesis. Radiation induces DNA damage. The nucleotide analogs generated by AdV-tk can prevent successful repair of radiation-induced DNA damage. In addition, since radiation induces membrane damage, it may facilitate the bystander effect by which nucleotide analogs diffuse from HSV-tk expressing cells to neighboring cells. Finally, radiation therapy promotes the infiltration of antigen presenting cells, such as dendritic cells, which present tumor associated antigens to T cells and then TK promotes the proliferation of those T cells.

Studies in murine prostate and mammary tumor models support these mechanisms and demonstrate synergy of AdV-tk + prodrug with radiation therapy (Chhikara *et al.* 2001, Vlachaki *et al.* 2001). Subcutaneous prostate tumors (generated with the RM-1 cell line) were treated by either intratumoral injection of AdV-tk followed by systemic prodrug, or local radiation therapy or the combination of AdV-tk + prodrug and radiation. Both single therapy modalities showed a 38% decrease in tumor growth compared to controls. The combined treatment resulted in a decrease of 61%. In addition, the combined therapy group had increased survival compared to the single therapy groups, which were increased compared to non-treated controls. This showed an improved local control when AdV-tk + prodrug was combined with radiation therapy.

A metastatic tumor model was used to analyze systemic antitumor activity after local AdV-tk and radiation delivery. Lung metastases were generated by tail vein injection of RM-1 prostate cancer cells on the same day that they were injected subcutaneously in the hind leg flank. The subcutaneous tumors were treated as before with intra-tumoral AdV-tk injection followed by prodrug, with local radiation or the combination of the two. The number of lung nodules was reduced by 37% following treatment of the flank tumor with AdV-tk, whereas radiotherapy alone had no effect on metastatic growth **(**Figure 3**)**. The combination of the two local deliveries, AdV-tk and radiation, lead to an additional 50% reduction in lung colonization. Primary tumors that received the combination therapy had a marked increase in CD4 T-cell infiltrate (Chhikara *et al.* 2001). This showed a systemic anti-tumor effect from local AdV-tk + prodrug treatment and demonstrated a synergistic effect when combined with radiation therapy.

Repeat delivery of AdV-tk + prodrug may be advantageous for some tumor types. However, in studies of systemic (intravenous) adenoviral vector delivery, the immune system thwarted repeat injections. In mammary and prostate tumor models, local delivery of the combination of AdV-tk + prodrug and radiation showed systemic antitumor effects on metastatic tumor growth

correlating with increased immune stimulation (Chhikara *et al.* 2001, Vlachaki *et al.* 2001). To address the question of whether multiple intratumoral vector injections would be feasible and advantageous, the effects of one to three courses of therapy were evaluated. In that model three courses of AdV-tk + prodrug plus radiation were found to be more effective than one or two courses (Vlachaki *et al.* 2001). This demonstrated that multiple vector injections were feasible and advantageous when the vector is delivered intratumorally.

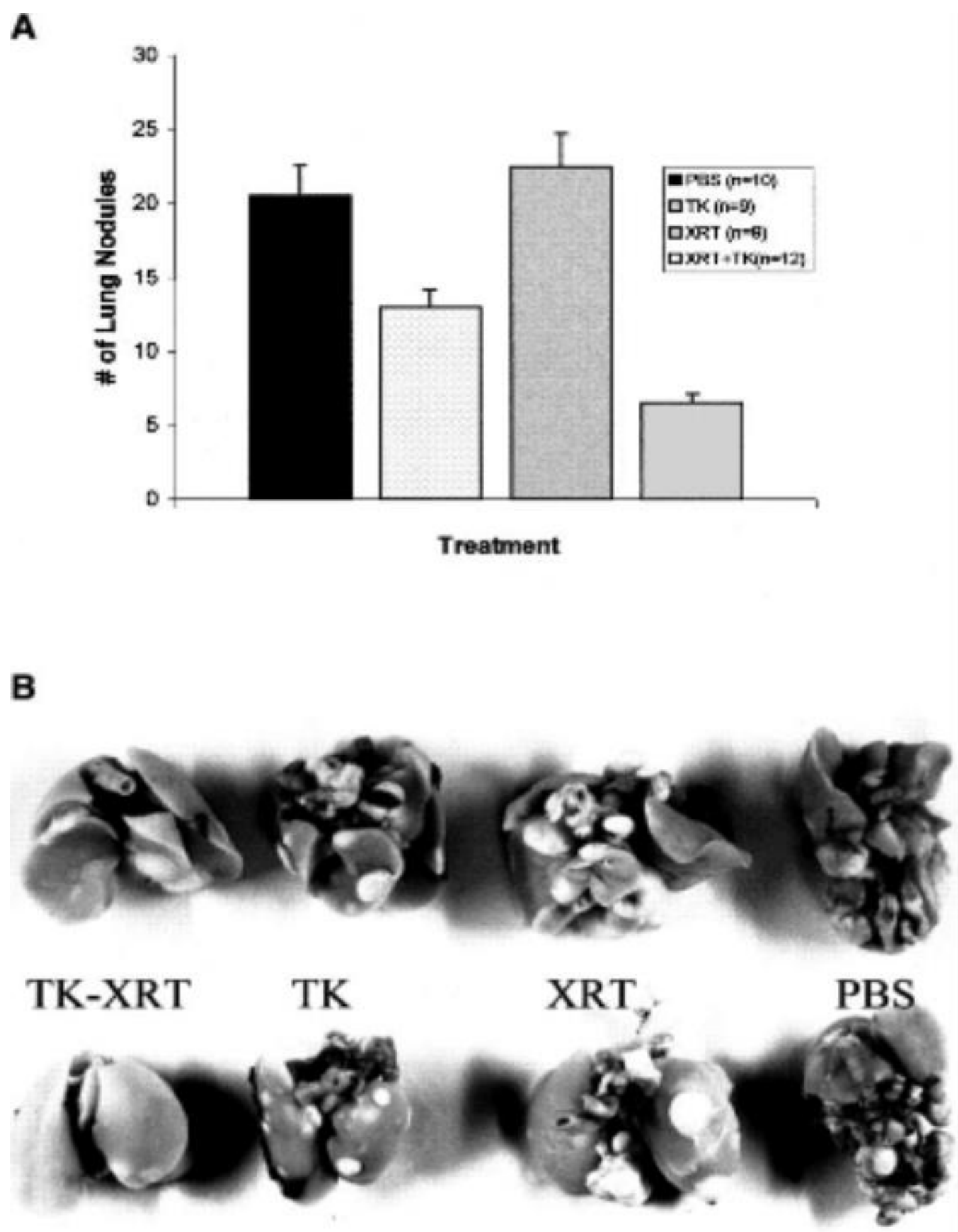

Figure 3: Local delivery of ProstAtak® had a significant effect on decreasing systemic metastatic tumor load

The combination of AdV-tk + prodrug (TK) and radiation therapy (XRT) delivered locally to the primary subcutaneous tumor (RM-1 prostate cancer cells in syngeneic C57bl mice) decreased the number of metastatic lung nodules. (A) Number of visible lung nodules 12 days after primary tumors were treated with single or combination therapy. (B) Two representative examples of lungs from each treated and control group. (From (Chhikara *et al.* 2001))

1.5 Clinical Trials of AdV-tk Therapy For Cancer

Multiple clinical trials using AdV-tk + prodrug have been completed or are ongoing in different tumor types, including prostate cancer, malignant gliomas, ovarian cancer, mesothelioma and pancreatic cancer (Herman *et al.* 1999, Shalev *et al.,* 2000, Trask *et al.* 2000, Hasenburg *et al.,* 2001, Miles *et al.* 2001, Teh *et al.* 2002, Sterman *et al.,* 2005, Van-der-Linden *et al.,* 2005, Aguilar *et al.,* 2006, Ayala *et al.,* 2006, Fujita *et al.,* 2006, Tetzlaff *et al.,* 2006). Overall this

approach has been safe and encouraging responses have been observed with more than 300 doses delivered to more than 150 patients. These trials are described in detail in the Investigator's Brochure and a summary of the results from multiple prostate cancer trials are described below.

1.5.1 Early Trials in Prostate Cancer- Safety, Biologic and Clinical Effects

The first Phase I study for prostate cancer used ultrasound guided intratumoral injection of AdV-tk followed by ganciclovir in men with recurrent prostate cancer after definitive radiation therapy. In that study four dose levels, from $2x10^9$ to $2x10^{12}$ vector particles, were evaluated ((Herman *et al.* 1999); Table 2). Three out of 18 patients (17%), one at each of the three higher dose levels, had objective responses defined as at least 50% decrease in PSA sustained for 6 weeks to 1 yr; a fourth patient had a short duration response despite early termination of GCV due to toxicity (Figure 4). Seventy percent (70%) of the participants had at least a temporary stabilization of their PSA. Two follow-on studies analyzed injections at multiple intraprostatic sites and multiple courses of AdV-tk + prodrug (Shalev *et al.* 2000, Miles *et al.* 2001). No significant toxicity was observed, and PSA responses were again observed in patients retreated after recurrent PSA rises. Additional evidence of clinical activity was a significant increase in mean PSA doubling time (PSA-DT), from 15.9 months before treatment to 42.5 months after treatment (Miles *et al.,* 2001). PSA reduction (PSAR) was observed immediately after treatment in 27 of 36 patients, with a PSAR greater than 25% in 12 patients and greater than 50% in 5 patients (Miles *et al.* 2001). Evidence for AdV-tk transduction, tumor cell cytotoxicity and immune cell infiltration were demonstrated in biopsy specimens after AdV-tk + GCV treatment (Miles *et al.* 2001). Immune effects included the observation that the level of $CD8^+$ T cells correlated with the number of apoptotic tumor cells in biopsy samples 2 weeks after AdV-tk injection (Miles *et al.* 2001). In addition, at 2 weeks, the percentage of activated (DR+) $CD8^+$ T cells in the blood was increased significantly. These clinical study results show the immune consequences of AdV-tk in patients and reflect, in humans, the immune effects observed from AdV-tk in preclinical models.

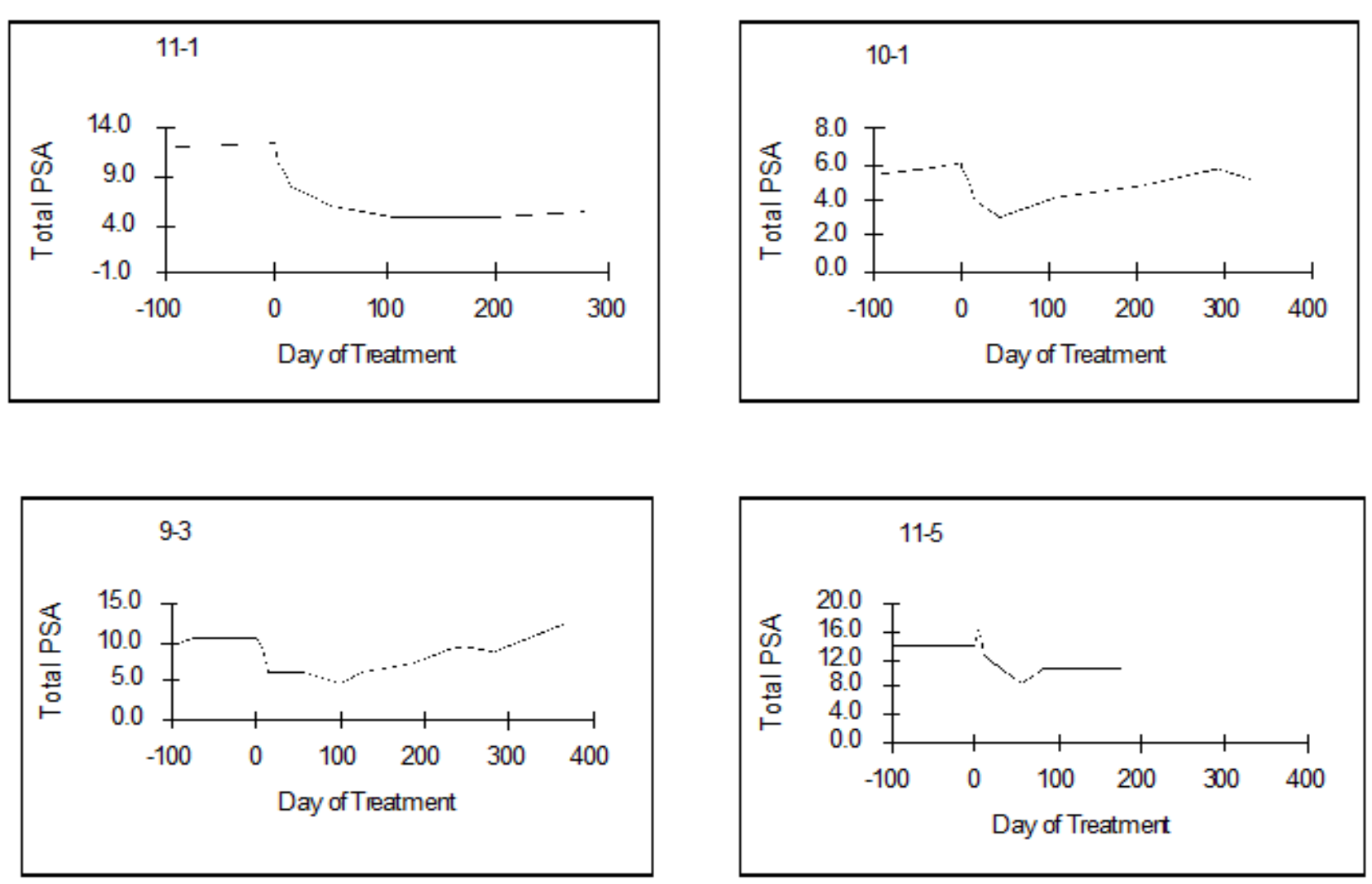

Figure 4: Objective clinical responses from Phase I trial in recurrent radiation-failure prostate cancer

Patients with recurrent organ-confined prostate cancer and rising PSA levels after definitive radiation therapy were treated with a single course of AdV-tk plus prodrug and their PSA followed through time. One patient from each of the three highest dose cohorts had objective partial responses (>50% reduction of PSA) and a second patient from the highest dose group (11-5) showed a significant PSA decline despite a shortened prodrug course.

Three neoadjuvant pre-prostatectomy studies involving a total of 41 patients with dose levels ranging from $1x10^{11}$ to $8x10^{11}$ vp (shown in Table 2) were conducted in patients with high risk of recurrence (Van-der-Linden *et al.* 2005, Ayala *et al.* 2006, Rojas-Martínez *et al.*, 2013). AdV-tk transduction, distribution and expression were systematically analyzed in one of these studies (Rojas-Martínez *et al.* 2013). Vector DNA with wide distribution throughout the prostate was demonstrated in all prostatectomy samples analyzed 7 to 15 weeks post-injection (Rojas-Martínez *et al.* 2013). Pathologic responses in prostatectomy specimens were seen in all cases analyzed and included loss of glandular architecture, widespread necrosis and apoptosis and significant immune cell infiltration (Ayala *et al.* 2000, Rojas-Martínez *et al.* 2013). $CD8^+$ T cells and $CD68^+$ macrophages were increased 3-4 fold in tumors after AdV-tk + prodrug treatment as compared to untreated controls. The effects were mostly confined to tumor tissue. Recently, additional biologic response determinants were described in 23 patients in one of the pre-prostatectomy studies (Ayala *et al.* 2006). Increased CD8 T cell levels were demonstrated in tumor samples and in blood samples of treated patients as well as increased IL-12 levels. In addition, an anti-angiogenic effect was demonstrated by decreased microvessel density in tumor samples (Ayala *et al.* 2006).

1.5.2 Phase 2a Trial of ProstAtak® with Radiotherapy

Based on the encouraging results in early clinical trials and the animal studies showing cooperative effects with radiation therapy, a Phase 2a clinical trial was conducted evaluating the combination of ProstAtak® with radiation therapy in patients with newly diagnosed, non-metastatic prostate cancer (Teh *et al.* 2001, Teh *et al.* 2004). The study was comprised of three arms based on the disease prognostic risk characteristics and differences in standard of care:

1. Arm A: Low risk subjects (PSA<10, Gleason score≤6 and clinical stage T1-T2a)
2. Arm B: Intermediate-High-risk subjects (PSA≥10 or Gleason>6 or clinical stage T2b-T3)
3. Arm C: Subjects with positive pelvic lymph nodes (stage D1)

Arm A subjects received two intraprostatic injections of AdV-tk two weeks apart, each followed by two weeks of valacyclovir, with radiation starting 24 to 72 hours after the first injection. Arm B and C subjects received three AdV-tk injections: #1-at the initiation of ADT, two months before initiation of radiation, #2-at the start of radiation and #3-2 weeks after start of radiation. All AdV-tk injections were at $5x10^{11}$ vector particles (vp) each and the valacyclovir dose was fixed at 2g orally three times per day for 14 days. Radiotherapy was delivered by IMRT to a prescribed dose of 70Gy (mean dose 76Gy) with an additional 45Gy to pelvic lymphatics for arm C.

Table 2: Summary of completed prostate cancer trials with AdV-tk

Patient Characteristics	Treatment Specifics	Toxicity	Efficacy
**Recurrent prostate cancer**<br><br>18 patients<br>(Herman *et al.* 1999)	**Dose escalation:**<br>$2x10^9$ vp, (4 pts)<br>$2x10^{10}$vp, (5 pts)<br>$2x10^{11}$vp, (4 pts)<br>$2x10^{12}$vp, (5 pts)<br><br>**Delivery:** Intraprostatic<br>**Prodrug:** GCV<br>**Concurrent tx:** none	1 patient on 4th dose level had transient grade 4 thrombocytopenia and grade 3 liver transaminase elevation	3 patients, one at each of highest dose levels, had **objective response** defined as at least 50% decrease in PSA sustained for 6 weeks to 1 yr
**Recurrent prostate cancer, multiple injections**<br><br>18 new patients plus 9 additional injections in patients from first study (Shalev *et al.* 2000, Miles *et al.* 2001)	**Dose:** 2-6 $x10^{11}$vp per injection<br><br>**Delivery:** Intraprostatic<br><br>**Prodrug:** GCV<br><br>**Concurrent tx:** none	Grade 3 hypertension requiring anti-hypertensive therapy in 1 patient	1. Increased mean PSA-DT from 15.9 to 42.5 months<br>2. Mean PSA reduction of 28%.<br>3. Time to return to initial PSA 8.5 months.
**Pre-prostatectomy**<br><br>41 patients in 3 studies<br>**10 pts:** (Rojas-Martínez *et al.* 2013)<br>**8 pts:** (Van-der-Linden *et al.* 2005)<br>**23 pts:** (Ayala *et al.* 2006)	10 pts: $1x10^{11}$ vp<br><br>8 pts: $2x10^{10}$ vp (4pts)<br>$2x10^{11}$ vp (4pts)<br><br>23 pts :2-$8x10^{11}$ vp	No significant toxicity attributable to study drug.	Pathologic effects in prostatectomy specimens.
**Newly diagnosed prostate cancer, combined with radiation therapy**<br><br>71 patients<br>(Teh *et al.* 2001, Teh *et al.* 2004)	**Dose:** $5x10^{11}$vp per injection<br><br>**Delivery:** Intraprostatic<br><br>**Prodrug:** valacyclovir<br><br>**Concurrent tx:** radiation +/- hormone	1. Grade 3 ALT elevation in 1 patient, resolving after 1 week, no interruption in therapy<br>2. Grade 3 GU toxicity in 1 patient attributed to radiation therapy	Lower PSA nadir compared to matched control group. Increased pathologic responses and FFF compared to historical controls.

1.6 AdV-tk and Radiation Therapy for Newly Diagnosed Prostate Cancer - Phase 2a Results

The Phase 2a trial enrolled 71 subjects, 33 in Arm A, 33 in Arm B and 5 in Arm C. A total of four subjects were withdrawn from the study before completion due to non-compliance or intolerance to valacyclovir. All other subjects completed the experimental intervention without significant toxicity and received radiation and ADT as per the standard of care. The incidence and severity of gastrointestinal and genitourinary toxicity was not significantly different than typically observed with radiation alone. Due to the paucity of patients presenting with stage D1 in the US, the number of patients on Arm C was small and therefore this population was not pursued further. At last follow up, three subjects on Arm C had developed metastatic disease despite having no evidence of local progression and one subject developed local recurrence. Treatment failures are not unexpected for this very high-risk category. **Positive results were observed through evaluation of three different endpoints in Arm A and B.**

1.6.1 Phase 2a PSA Nadir Results

PSA nadir level has been shown to be predictive of disease free survival and distant metastasis-free survival in patients treated with radiation therapy for prostate cancer, (Crook *et al.,* 2000, Ray *et al.,* 2006). In the ProstAtak® Phase 2a study, PSA data from subjects in Arm A were compared to data from a radiation only group matching the Arm A criteria (T1-T2a, Gleason<7, PSA<10) which received the same IMRT dose during the same treatment period at the same institution (Table 3). The frequency of patients in Arm A with PSA nadir ≤0.2 ng/ml was 77% compared to 58% in the control group. There was a positive trend but the numbers were not sufficient to reach statistical significance.

Table 3: PSA Nadir Comparison

Therapy	Post-tx PSA Nadir ≤ 0.2 ng/ml	N	Median Follow Up (months)	Pre-tx Median PSA ng/ml
Arm A<br>ProstAtak® + RT	**77%**	31	53	5.1
Control group<br>RT only	**58%**	31	48	5.4

1.6.2 Improvement in Pathologic Complete Response

Multiple studies of radiation therapy for prostate cancer have demonstrated the value of pathological complete response (pCR), two years after radiation, as an indicator of local control which correlates with long-term freedom from failure, distant metastasis-free survival and cause-specific survival (Crook *et al.* 2000, Zelefsky *et al.* 2001, Pollack *et al.,* 2002, Crook *et al.,* 2004, Vance *et al.,* 2007, Zelefsky *et al.,* 2008). In the ProstAtak® Phase 2a study, sextant biopsies were performed 2-years post radiation. The data obtained were compared to published data from multiple radiation therapy studies (Table 4**)**. Of 40 available sextant biopsies, 22 from Arm A and 18 from Arm B, 37 were negative (93%) with the distribution in Arm A and B shown in Table 4. This pCR rate is significantly higher than comparable, contemporaneous studies. The patient populations and treatment regimens between the studies were varied but comparable. The studies included low, intermediate and high risk patients based on T stage, Gleason and pre-treatment PSA. The dose of radiation to the prostate in both Crook et al., studies was 66Gy (Crook *et al.* 2000, Crook *et al.* 2004). In Crook et al, 2000, approximately one third of subjects received ADT prior to radiation whereas in crook et al, 2004, all patients were randomized to receive 3 or 8 months of ADT. In the Pollack et al study, from MDACC, 87 subjects received 70Gy and 81 received 78Gy without a significant difference in clinical or biopsy outcome between the groups (Vance *et al.* 2007). In Zelefsky et al, from MSKCC, radiation doses ranged from 65-81Gy and 39% received ADT. In the ProstAtak® Phase 2a study, the radiation dose was 76Gy and only the Arm B patients received ADT.

Table 4: 2-Year Biopsy Results in Multiple Contemporaneous Radiation Studies

Therapy	# Tumor Free (%) in sextant biopsy	N	Compared to ProstAtak®
Rad 66 Gy (Crook *et al.* 2000)	39 (37%)	105	P<0.0001
Rad Alone 70-78 Gy (Pollack *et al.* 2002)	118 (70%)	168	P=0.002
Rad + ADT (Crook *et al.* 2004)	148 (72%)	205	P=0.005
Rad 65-81 Gy (Zelefsky *et al.* 2001)<br>Subset analysis (Zelefsky *et al.* 2008)<br>Intermediate Risk </=70.2 Gy<br>Intermediate Risk >/=75.6 Gy	185 (73%)<br><br>58%<br>76%	252	P=0.009
**ProstAtak® (Arm A/B)**	**37 (93%)**	**40**	
Arm A (ProstAtak® + Rad 76Gy)	20 (91%)	22	
Arm B (ProstAtak® + Rad 76Gy + ADT)	17 (94%)	18	

1.6.3 Freedom From Failure Outcomes

Freedom from failure (FFF) and disease free survival (DFS) are the endpoints used by most studies that have advanced the standard of care for up-front prostate cancer (Pilepich *et al.* 2001, Pollack *et al.* 2002, DeWeese *et al.*, 2005, Zietman *et al.* 2005, Roach *et al.*, 2006, Zelefsky *et al.* 2006, Kuban *et al.* 2008). Preventing or delaying failure has significant clinical benefit because it prevents or delays the need for non-curative, toxic ADT and subsequent development of painful metastatic disease. These endpoints are also important because overall survival evaluation is confounded by competing comorbidities due to the long natural history of prostate cancer and older age group of affected individuals. FFF, with failure defined as biochemical or clinical failure whichever occurs first, has been the most commonly used endpoint in the PSA era for prostate cancer studies.

Most prostate cancer patients (70-100%) develop biochemical failure first and begin treatment with palliative salvage ADT, with or without chemotherapy, prior to the development of verified local, regional or distant treatment failure or death from prostate cancer. The currently preferred definition for biochemical failure following radiation therapy is the "nadir + 2" definition, also referred to as the 2006 ASTRO consensus definition, which was recommended by a consensus panel of clinical experts (Roach *et al.* 2006). This defines failure as the time point when the PSA rises by 2ng/ml or more above the nadir PSA. According to D'Amcio et al, PSA failure defined with the "nadir + 2" definition "has been shown to be significantly associated with a greater risk of metastases and cancer death" (D'Amico *et al.* 2008). This is the definition used in the ProstAtak® Phase 2a study (Figure 5) and most of the contemporaneous studies used for comparison in Table 5.

For the ProstAtak® Phase 2a study, FFF was 100% for Arm A and 90% for Arm B (95% for intermediate, 75% for high risk) after a median follow up of 68 months. This appears to represent a significant improvement over current standard of care, for which the best reported results in comparable patients are 66-88% in low risk, 55-79% in intermediate risk and 33-67% in high risk (Crook *et al.* 2004, Peeters *et al.* 2006, Zelefsky *et al.* 2006, Dearnaley *et al.* 2007, D'Amico *et al.* 2008, Crook *et al.*, 2009). Because the year of treatment has been shown to be an independent predictor of FFF (Kupelian *et al.* 2005), studies in which treated patients during the same years and with similar follow up were selected for comparison (Table 5).

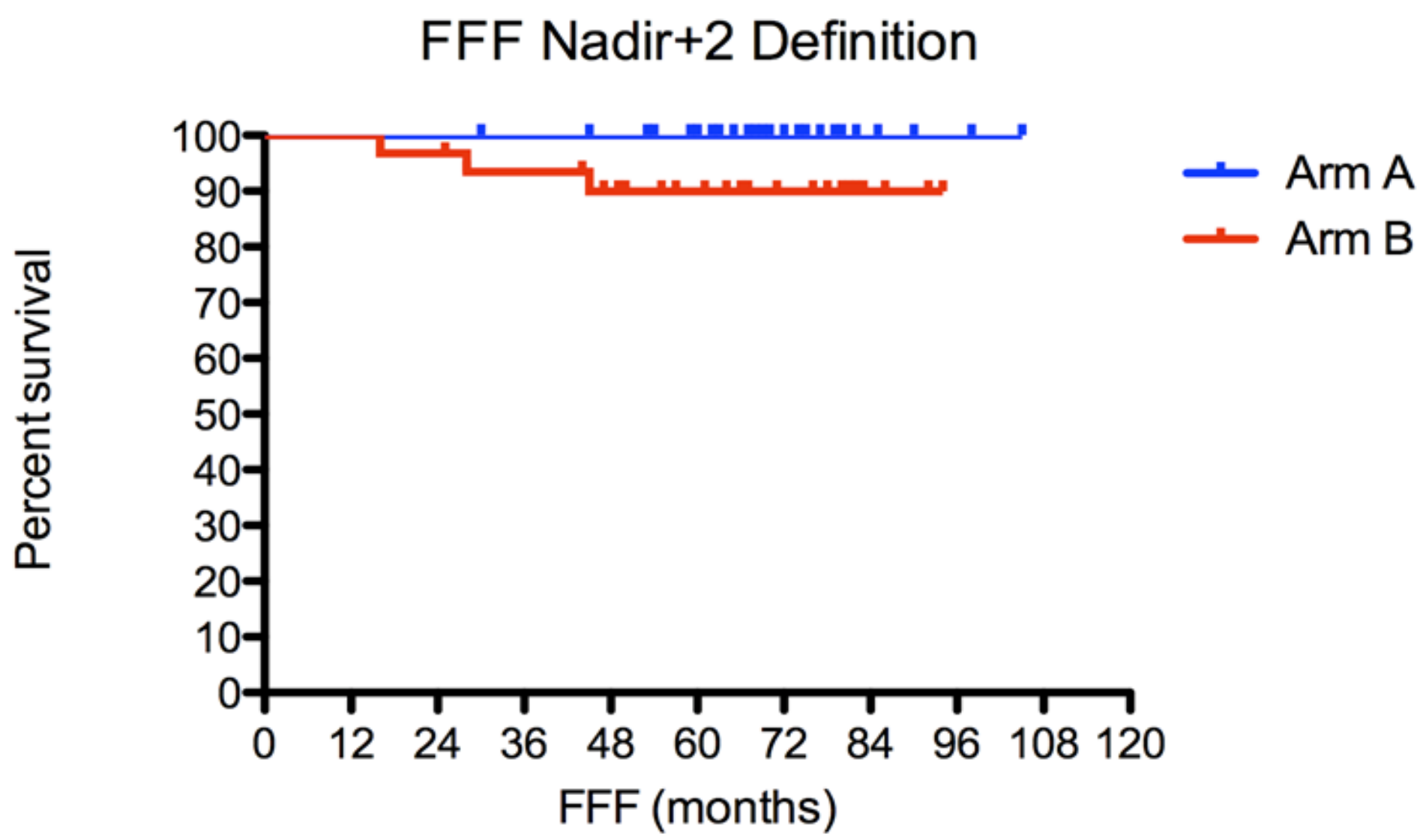

Figure 5: Kaplan-Meier Freedom from Failure (FFF) Curves Using Nadir+2 Definition

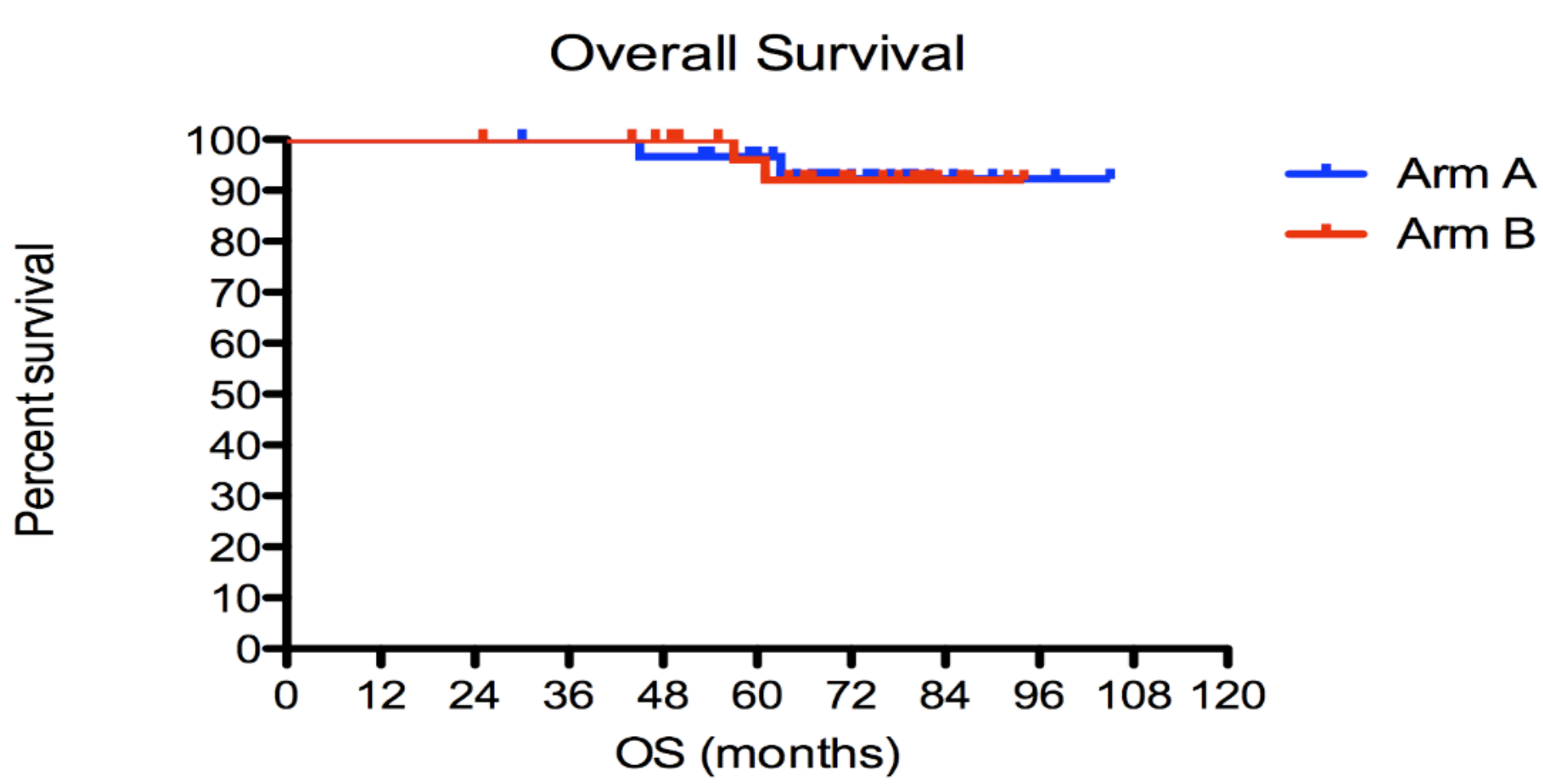

Figure 6: Kaplan-Meier Overall Survival for ProstAtak® Phase 2a Study

Table 5: Freedom from Failure Comparison to Contemporary Studies*

Therapy	N	Median Follow Up	Therapy Arms	FFF Total	FFF by Risk Group		
					Low	Intermed	High
Rad 66Gy + 3 vs 8 mos ADT (Crook *et al.* 2004, Crook *et al.* 2009)	361	6.6 yrs	3 mos ADT	58%	66%	66%	33%
			8 mos ADT	65%	88%	55%	59%
Rad 68 vs 78 Gy +ADT in 21% (Peeters *et al.* 2006)	664	4.3 yrs	68 Gy	54%	87%	64%	48%
			78 Gy	64%	84%	79%	56%
Rad 64 Gy vs 74 Gy 3-6mos ADT both arms (Dearnaley *et al.* 2007)	843	5 yrs	64 Gy	60%	79%	70%	43%
			74 Gy	71%	85%	79%	57%
Rad 81 Gy +ADT in 53% (Zelefsky *et al.* 2006)	561	7 yrs	N/A		89%	78%	67%
Rad 70 Gy +/- 6 mos combined ADT (D'Amico *et al.* 2008)	206	8.2 yrs	No ADT			66%	
			6 mos			75%	
**Rad 76Gy + ProstAtak®** +ADT intermed-high risk (Teh *et al.* 2004, Aguilar *et al.* 2006)	**62**	**5.7 yrs**	**N/A**	**95%**	**100% (31/31)**	**90% (28/31)**	
						**95% (20/21)**	**80% (8/10)**

Table 6: Study Details for Table 5

Study	Years of Treatment	FFF Definition	Biochemical Failure Definition
(Crook *et al.* 2004, Crook *et al.* 2009)	1995-2001	Survival free of biochemical or clinical failure	Nadir +2
(Peeters *et al.* 2006)	1997-2003	Freedom from biochemical or clinical failure, whichever was first	1996 ASTRO with backdating
(Dearnaley *et al.* 2007)	1998-2002	bPFS defined as biochemical or clinical failure or death from prostate cancer whichever was first	Nadir +2
(Zelefsky *et al.* 2006)	1996-2000	PSA failure, no clinical failures before biochemical	Nadir +2
(D'Amico *et al.* 2008)	1995-2001	PSA failure, no clinical failures before biochemical	Nadir +2
ProstAtak® Phase 2a	1999-2003	Freedom from biochemical or clinical failure, whichever was first	Nadir + 2

1.6.4 Survival Endpoints

Overall survival for the Phase 2a study was very good (Figure 6) with only one of four reported deaths resulting from prostate cancer. This is consistent with published studies of newly diagnosed prostate cancer in which most deaths are from causes unrelated to prostate cancer making prostate cancer specific deaths, as an endpoint, untenable for evaluation of prostate cancer treatments. Increased radiation dose, for example, has been adopted as the standard of care after demonstrating improved FFF, despite the inability to demonstrate an improvement in overall survival (Pollack *et al.* 2002, Zietman *et al.* 2005, Peeters *et al.* 2006, Zelefsky *et al.* 2006, Dearnaley *et al.* 2007, Al-Mamgani *et al.* 2008, Kuban *et al.* 2008). Several studies in the more advanced or unfavorable risk patients have demonstrated some overall survival improvement with the addition of adjuvant ADT (Lawton *et al.* 2001, Bolla *et al.* 2002, D'Amico *et al.* 2004). Two of these studies (Lawton *et al.* 2001, Bolla *et al.* 2002) used long term ADT for 2-3 years and thus incurred a significant compromise to quality of life, diminishing the clinical benefit. Recent evidence demonstrates that even the use of ADT for short periods (6 months) may be associated with increased risk of non-prostate cancer related death in patients with cardiovascular co-morbidities(D'Amico *et al.* 2008).

Despite higher doses of radiation and adjuvant ADT, recurrent prostate cancer still develops in more than 60,000 patients per year in the US who face an incurable, costly problem treated with palliative ADT with or without chemotherapy. Furthermore, more than 28,000 men still die from prostate cancer each year in the US (American Cancer Society Facts and Figures 2008). Delaying or avoiding clinical failure would prevent a significant portion of the patient and societal prostate cancer costs.

1.6.5 Immunological Effects

In addition to the clinical evidence for ProstAtak® activity, immunologic activity was also evaluated in the ProstAtak® Phase 2a trial (Satoh *et al.* 2004, Fujita *et al.* 2006). The percentage of activated $CD8^+$ T cells in the peripheral blood increased significantly after treatment and this increase was greater in the ProstAtak® with radiation combination than in the previous trials of AdV-tk + prodrug as monotherapy. In addition, significant increases in activated $CD4^+$ T cells were also observed after treatment in this trial. The increases in CD4 and CD8 T cells were maintained for 8-12 months (Fujita *et al.* 2006).

1.6.6 Rationale for Randomized Trial Design

The Phase 2a results suggest that ProstAtak® combined with radiation therapy ± ADT may improve the outcome of all patients with prostate cancer. However, this was an open label, non-randomized study. A randomized controlled trial is necessary to confirm these preliminary results.

2.0 OBJECTIVES

The overall objective for this study is to determine if adjuvant therapy with ProstAtak® improves the clinical outcome of radiation therapy for newly diagnosed prostate cancer.

2.1. Primary endpoints
- **2.1.1.** Disease free survival

2.2. Secondary Endpoints
- **2.2.1.** PSA nadir
- **2.2.2.** Freedom from biochemical failure
- **2.2.3.** Prostate cancer specific survival
- **2.2.4.** Overall survival
- **2.2.5.** Quality of Life Assessment
- **2.2.6.** Immune stimulation

3.0 PATIENT SELECTION

3.1 Inclusion Criteria

- **3.1.1.** Histologically confirmed adenocarcinoma of the prostate
- **3.1.2.** Patients meeting any of the NCCN intermediate risk criteria **OR** subset of NCCN high risk group that has a single high-risk feature defined as follows:

Table 7: NCCN Risk Criteria

**NCCN Intermediate Risk**	Having at least one of the following:<br>☐ PSA 10-20 ng/ml<br>☐ Gleason score =7<br>☐ T2b-T2c*
**OR:**	
**NCCN High Risk with single high-risk factor**	Having **ONLY ONE** of the following:<br>☐ PSA>20 ng/ml<br>☐ Gleason score 8-10<br>☐ T3a*
Excluded are those in the following risk groups:<br>• Low risk<br>• High risk with more than 1 high risk factor<br>• Locally advanced/very high risk=T3b-T4<br>• Metastatic: N1 or M1	
***Stage is based on the 2010 AJCC TNM clinical staging**	

- **3.1.3.** Patients must be planning and medically able to undergo standard prostate-only external beam radiation therapy and able to tolerate multiple transrectal ultrasound guided injections
- **3.1.4.** 18 years of age or older
- **3.1.5.** Performance status must be ECOG 0-2
- **3.1.6.** The following laboratory criteria must be met:
  - 3.1.6.1. SGOT (AST) <3x upper limit of normal
  - 3.1.6.2. Serum creatinine < 2mg/dl

3.1.6.3. Calculated creatinine clearance >30ml/min

3.1.6.4. WBC > 3000/mm^3

3.1.6.5. Platelets>100,000/mm^3

Cr clearance formula=
[(140 – age in yr) x (weight in kg)] ÷ [72 x serum Cr in mg/dL]

3.1.7. Patients must agree to have prostate biopsy two years after end of radiation

3.1.8. Patients must give study specific informed consent prior to enrollment

3.2 Exclusion Criteria

3.2.1 Active liver disease, including known cirrhosis or active hepatitis

3.2.2 Patients on systemic corticosteroids (>10 mg prednisone per day) or other immunosuppressive drugs

3.2.3 Known HIV+ patients

3.2.4 Regional lymph node involvement or distant metastases

3.2.5 Patients planning to receive whole pelvic irradiation

3.2.6 Other current malignancy (except squamous or basal cell skin cancers)

3.2.7 Other serious co-morbid illness or compromised organ function that, in the opinion of the investigator, would interfere with treatment or follow up. For example, patients with diseases that preclude radiation therapy to the prostate such as severe prostatitis and inflammatory bowel disease.

3.2.8 Prior treatment for prostate cancer except TURP or ADT, if ADT given for a maximum of 6 months. If prior TURP, patients must be deemed able to receive multiple intra-prostatic injections by the investigator.

3.2.9 Patients who had or plan to have orchiectomy as the form of hormonal ablation

3.2.10 Known sensitivity or allergic reactions to acyclovir or valacyclovir

4.0 PRETREATMENT EVALUATION

4.1 The following evaluations performed within the specified time prior to study enrollment:

4.1.1 Within 12 months, prostate biopsy with histologic evaluation and assignment of Gleason score- when feasible, slides from the diagnostic biopsy should be provided to Advantagene for central archiving.

4.1.2 Within 6 months and prior to ADT start, PSA (the specific assay used will be recorded on the CRF)

4.2 The following evaluations performed ≤ 4 weeks prior to first AdV-tk or placebo injection:

4.2.1 Clinical assessment to include history and physical exam, DRE and ECOG performance status assessment

4.2.2 Quality of Life assessment by questionnaire

4.2.3 CBC, differential, platelets

4.2.4 Creatinine, AST, ALT, Alk. Phos. and bilirubin

4.2.5 Optional blood collection for immunology research studies

4.3 All patients will also undergo all standard of care and clinically indicated evaluations and the data will be submitted to Advantagene. This will include a bone scan if PSA >20ng/ml or Gleason >7 or stage T3a or if patient is symptomatic. Pelvic CT or MRI will be performed prior to enrollment for high-risk patients.

5.0 REGISTRATION PROCEDURES

This is a double-blind placebo-controlled trial. Clinical site personnel will determine eligibility of prospective subjects and complete an Eligibility Checklist form (CRF EC) according to the Patient Registration SOP (SOP PR). The completed form EC will be sent to Advantagene where a case number will be assigned, randomization will be performed, and blinded treatment assignment will be made. This information will be communicated back to the clinical site via the Patient Registration form (CRF PR).

6.0 TREATMENT PLAN

6.1 Overview

6.1.1 Patients will be randomized to test or control at a 2:1 ratio; two identical test arms and one control arm

Test arms: ProstAtak® + radiation therapy +/- ADT

Control Arm: Placebo + valacyclovir + radiation therapy +/- ADT

Patients on the Test arms will receive three courses of ProstAtak® each consisting of AdV-tk injection + oral valacyclovir. Patients in the Control Arm will receive corresponding courses of placebo injection + oral valacyclovir. AdV-tk or placebo injection will be delivered to the prostate via TRUS-guided injection as follows:

1. 1st injection is at least 2 weeks prior to initiation of radiation (to allow 14-day valacyclovir course completion) and not more than 8 weeks before starting radiation.
2. 2nd injection is on the day of or up to 3 days before initiation of radiation therapy and at least 2 weeks after injection #1 (to allow 14-day valacyclovir course completion). Injection may be on the last day of valacyclovir for the previous course.
3. 3rd injection is 2-3 weeks after injection #2 (to allow 14-day valacyclovir course completion). Injection may be on the last day of valacyclovir for the previous course.

The prodrug, valacyclovir, will be administered at a fixed dose for 14 days after each AdV-tk or placebo injection starting the day after the injection.

6.2 ProstAtak® Administration

6.2.1 Delivery of AdV-tk or Placebo Injection
(see Vector Storage and Administration SOP, SOP VS, for details)

6.2.1.1 Transrectal ultrasound (TRUS) will be used to visualize the prostate and guide delivery to four sites, one apical and one basal in each lobe.

6.2.1.2 For the Test arms, the dose of AdV-tk will be $5x10^{11}$ vector particles total for each course. The dose will be in 2 ml total volume and approximately 0.5 ml will be injected into each of the four sites.

6.2.1.3 For the Control Arm, the placebo solution will appear the same as the vector solution and will also consist of 2ml total volume and approximately 0.5 ml will be injected into each of the four sites.

6.2.1.4 For prostate injections, the same prophylactic antibiotic and other preparations as appropriate for prostate biopsy should be used.

6.2.2 Prophylactic medications

6.2.2.1 Prophylactic treatment with acetaminophen alternating with ibuprofen is to be given for the first 24-48 hours following injection to prevent pain, fever and flu-like symptoms possibly associated with injections.

6.2.2.2 In addition, standard prophylactic antibiotic treatment such as ciprofloxacin is recommended with the biopsy and injection procedures.

6.2.3 Valacyclovir Administration

(see Prodrug Storage and Administration SOP, SOP PS, for details)

6.2.3.1 On the day of each AdV-tk or placebo injection, the subject will be given a bottle containing the appropriate number of caplets and a diary to record each dose taken.

6.2.3.2 The subject will be instructed to begin taking the caplets on the day following the AdV-tk or placebo injection and continue for 14 days.

6.2.3.3 The dose of valacyclovir will be 2 grams orally three times per day for 14 days if the calculated creatinine clearance is greater than 50 ml/min. The dose should be adjusted for renal impairment based on the calculated creatinine clearance as follows:

Table 8: Valacyclovir Dosage for Calculated Creatinine Clearance

**Creatinine clearance (ml/min)**	**Valacyclovir dosage**
50 or greater	2 grams three times a day
25 to less than 50	1.5 grams three times a day
10 to less than 25	1.5 grams twice a day
less than 10 or dialysis	Hold valacyclovir

6.2.3.4 Creatinine clearance will be calculated at the time of trial enrollment, and recalculated if creatinine becomes abnormal, using the following formula:

Cr clearance (ml/min)= [(140 – age in yr) x (weight in kg)] ÷ [72 x serum Cr in mg/dL]

6.2.4 Treatment Escape Rules

6.2.4.1 Fever may occur due to the local inflammatory and immune response to AdV-tk, which will be treated with anti-pyretics as necessary. If local inflammation is extensive or infection develops, valacyclovir may be stopped and antibiotics and/or anti-inflammatory therapy given as appropriate.

6.2.4.2 If a patient experiences side effects related to valacyclovir (described in section 8.3), valacyclovir may be stopped at the discretion of the investigator. The sponsor must be notified.

6.2.4.3 If a patient experiences toxicity or side effects with the first or second course of treatment, subsequent courses may be withheld at the discretion of the investigator. The sponsor must be notified.

6.3 Radiation Therapy

6.3.1 Technical Factors

6.3.1.1 Megavoltage equipment is required with effective photon energies of at least 6 MV.

6.3.2 Localization, Simulation and Immobilization

6.3.2.1 A urethrogram is recommended, but not required, to establish the most inferior portion of the prostate. A treatment planning CT scan will be required to define tumor, clinical, and planning target volumes as well as the critical normal structures. The treatment planning CT will be acquired with the patient set up in the same position as for daily treatments. The CT scan of the pelvis should start at or above the iliac crest down to the perineum. All tissues to be irradiated must be included in CT scan. CT scan thickness should be not more than 0.5 cm through the region that contains the target volumes (i.e., from the bottom of the sacroiliac joints down to the penile urethra). The regions above and below the target volume region may be scanned with slice thickness not more than 1.0 cm.
Patients who do not have pre-enrollment CT/MRI and are found to have locally advanced disease on the radiation therapy treatment planning CT/MRI, will continue on study as planned and be evaluated as per intent to treat.

6.3.2.2 The GTV, CTV, and PTV and relevant normal tissues must be outlined on all CT slices in which the structures exist.

6.3.2.3 Daily target localization (fiducial markers, transabdominal ultrasound or kV- or MV-CT) is required.

6.3.2.4 Prostate gland immobilization with rectal balloon will be allowed for those sites routinely employing such techniques.

6.3.2.5 Image Guided Radiation Therapy is required if using a hypofractionation regimen

6.3.3 Treatment Planning/Target Volumes *(the specific doses listed in this section are for a total of 78Gy to the PTV in 2Gy fractions but may be modified as per local standard of care to accommodate the range of prescription doses allowed in section 6.3.5 including moderate hypofractionation with 60 Gy to the PTV in 3Gy fractions)*

6.3.3.1 The Gross Tumor Volume **(GTV)** is defined by the physician as all known disease as defined by the planning CT, urethrogram, and clinical information. The GTV for the purposes of this protocol is the prostate only. If a urethrogram is used, the GTV will encompass a volume inferiorly 10 mm superior to the tip of the dye and no less than the entire prostate. Prostate dimensions should be defined as visualized on CT scan.

6.3.3.2 The Clinical Target Volume-1 **(CTV-1)** is the GTV (the prostate) plus the seminal vesicles.

6.3.3.3 The Planning Target Volume-1 **(PTV-1)** is defined as the PTV for the initial 46 Gy. It will provide a margin around the CTV-1 to compensate for the variability of treatment set up and internal organ motion. A minimum of 5 mm around the CTV-1 is required to define the PTV-1. Up to 10 mm may be used in all directions.

6.3.3.4 The Clinical Target Volume-2 **(CTV-2)** is the GTV (the prostate) only.

6.3.3.5 The Planning Target Volume-2 **(PTV-2)** is defined as the second or "cone-down" PTV and will be treated after 46 Gy for an additional 32 Gy. It will provide a margin around the CTV-2 to compensate for the variability of treatment set up and internal organ motion. A minimum of 5 mm around the CTV-2 is required to define the PTV-2. The posterior margin should be 5-7 mm.

6.3.3.6 Treatment will be given only to PTV-1 and PTV-2 using IMRT to exclude as much of the bladder and rectum as possible. Field arrangements will be determined by inverse-based planning to produce the optimal plan in accordance with volume definitions.

6.3.4 Critical Normal Structures

6.3.4.1 Dose-volume histograms (DVHs) must be generated for all critical normal structures.

6.3.4.2 Portions of the bladder and rectum will, by necessity, receive the full dose to the PTV; however, careful planning must be performed to ensure that the volume of the bladder and rectum receiving the full dose is kept to a minimum.

6.3.4.3 Based upon a review of patient dosimetry, the following normal tissue guidelines should be followed:

Rectum (fractionation up to 2 Gy):
No more than 15% volume of the rectum will receive more than 75 Gy.
No more than 25% volume of the rectum will receive more than 70 Gy.
No more than 35% volume of the rectum will receive more than 65 Gy.
No more than 50% volume of the rectum will receive more than 60 Gy.

Rectum (moderate hypofractionation, >2.4 Gy):
No more than 3% volume of the rectum will receive more than 60 Gy.
No more than 15% volume of the rectum will receive more than 57 Gy.
No more than 30% volume of the rectum will receive more than 53 Gy.
No more than 50% volume of the rectum will receive more than 49 Gy.
No more than 60% volume of the rectum will receive more than 41 Gy.

Bladder (fractionation up to 2 Gy):
No more than 15% volume of the bladder will receive more than 80 Gy.
No more than 25% volume of the bladder will receive more than 75 Gy.
No more than 35% volume of the bladder will receive more than 70 Gy.
No more than 50% volume of the bladder will receive more than 65 Gy

Bladder (moderate hypofractionation, >2.4 Gy):
No more than 5% volume of the rectum will receive more than 60 Gy.
No more than 25% volume of the rectum will receive more than 49 Gy.
No more than 50% volume of the rectum will receive more than 41 Gy.

6.3.4.4 Care should be taken not to shield the penile bulb at the expense of adequate coverage of the PTV in this study.

6.3.4.5 The normal tissue volume to be contoured will include bladder, rectum, bilateral femora (to the level of ischial tuberosity), penile bulb, and skin. The normal tissues will be contoured and considered as solid organs. The bladder should be contoured from its base to the dome, and the rectum from the anus (at the level of the ischial tuberosities) for a length of 15 cm or to the rectosigmoid flexure.

6.3.5 Prescription Dose

6.3.5.1 At least 95% of the PTV should receive the prescribed dose. The maximum dose to the PTV should not exceed the prescription dose by more than 7%.

6.3.5.2 Prescription dose to the PTV shall be 78Gy in 2 Gy dose fractions, 60 Gy in 3 Gy dose fractions or similar local standard (e.g. 79.2 Gy in 1.8 Gy dose fractions, 81 Gy in 1.8 Gy fractions or 70 Gy in 2.5 Gy fractions). All fields treated once daily, 5 fractions per week.

6.3.6 Dose Heterogeneity

6.3.6.1 Maximum dose to the PTV volume should not exceed the prescription dose by more than 7%.

6.3.6.2 The maximum point dose to critical normal structures outside the PTV should not exceed the prescription dose.

6.3.6.3 The treating physician must carefully consider the tolerance dose/volume to each critical normal structure and unspecified tissue.

6.4 Androgen Deprivation Therapy (ADT)

6.4.1 ADT is optional but needs to be decided before enrollment to allow for stratification at the time of randomization.

6.4.2 Patients who choose to receive ADT will receive standard of care short-term ADT not to exceed 6 months. The duration of anti-androgen use will be recorded on the CRF.

7.0 PATIENT ASSESMENT

7.1 Evaluation during and after treatment

7.1.1 All patients will undergo all standard of care and clinically indicated evaluations in addition to any study specific evaluations. Data will be collected for these non-specified evaluations.

7.1.2 Clinical assessment with history and physical including toxicity assessment will be performed prior to each AdV-tk or placebo injection, at follow up visits 1- 2 weeks after each injection (which may correspond to the pre-injection visit at

some time points) and at 3, 6, 12, 18 and 24 months after completion of radiation therapy. After this, clinical assessment of late toxicity and disease status will be conducted every 6 months through year 5 after which long-term follow up of general health status will continue yearly. Long-term survival and prostate cancer specific survSTUDival follow-up will continue for at least 10 years after completion of radiation. If disease progression/recurrence occurs or new serious health problem develops, this should be reported immediately to Advantagene.

7.1.3 CBC, differential, platelets, creatinine, AST, ALT, alkaline phosphatase and bilirubin will be monitored 1-2 weeks after each AdV-tk or placebo injection and repeated at subsequent visits if abnormal until return to baseline

7.1.4 PSA and DRE will be monitored as per standard of care at follow up visits after completion of radiation. For PSA measurements the specific assay used will be recorded on the CRF. As per current NCCN practice guidelines, the standard of care for evaluation of a positive DRE or rising PSA, defined as 2ng/ml above nadir, requires confirmation by prostate biopsy, bone-scan or abdomen/pelvic CT/MRI. Similar confirmation will be required for patients that are symptomatic. Patients with a positive bone scan do not need further evaluation but patients with local recurrence should be restaged with a bone scan as well as abdomen/pelvic CT/MRI.

7.1.5 Quality of life (QOL) will be assessed at 3, 6, 12, 18 and 24 months after completion of radiation therapy

7.1.6 Prostate biopsy (specifics described below in section 7.3) will be performed 22-26 months after completion of radiation therapy

7.1.7 Optional blood for immunology research studies (40-60 cc) will be collected before treatment initiation and 1-2 weeks after each AdV-tk or placebo injection, and at 3 and 12 months after completion of radiation

7.1.8 Long-term follow-up of general health status and any side effects may continue for life. This may be done by telephone contact or letter with the patient or their medical provider. Medical records may also be reviewed to assess for any potential side effects and for general health status.

7.2 Toxicity

7.2.1 Toxicity reporting will be done according to the Adverse Event SOP (SOP AE) and section 9.3 below.

7.2.2 Acute toxicity will be defined as side effects occurring until the completion of radiation and will be graded using the NCI Common Toxicity Criteria CTCAE ver4.0.

7.2.3 The Late Toxicity Monitoring period will begin after the Acute Toxicity Monitoring period and will continue for 5 years from the end of radiation and after that, long-term follow up will continue as described in 7.1.8 above. Data will be collected on new medical conditions according to categories including Allergy/Rheumatology, Cardiovascular, Endocrine, Hematology/Oncology and Neurologic. Genito-urinary and gastrointestinal toxicities, which are expected after prostate radiation therapy, will be graded using CTCAEver4.0.

7.3 Pathology

7.3.1 Central pathologic review: Inclusion and randomization will be done using the local pathology report. Central pathology review will be performed retrospectively from archived samples.

7.3.2 Archival tissue collection: The Sponsor will coordinate and implement collection and archival of pretreatment diagnostic material and post-treatment 2-year biopsy material. This will consist of the slides and/or paraffin blocks from the two biopsy encounters.

7.3.3 Post-treatment biopsies: All patients who have not already had declared failure will have post-treatment prostate biopsy done at 24 months (+/- 2 months) after completion of radiation. The preferred protocol for the 2-year biopsy is to sample at least 6 areas of the prostate (base, mid and apex on both sides). If the organ is deemed too small to physically allow for 6 cores, fewer cores may be taken as long as the rationale is provided, and size of prostate specified. All biopsies should be fixed in 10% neutral buffered formalin as soon as possible after the biopsy procedure. Regardless of the sampling technique employed, the location of the cancer in the original biopsy should be included. At least one representative core or H&E slide of each core will be submitted for central archive. Biopsy results will be classified as Negative (benign, high-grade PIN or suspicious for adenocarcinoma) or Positive (adenocarcinoma present, with or without treatment effect).

7.3.4 Central pathology review will be performed for post-treatment biopsies and retrospective review of baseline diagnostic pathologies. Sensitivity analyses will be performed to determine if imbalances of baseline characteristic deviations may have biased the results.

7.4 Quality of Life (QOL)

Patient-reported Health Related Quality of Life outcomes will be collected using the Expanded Prostate Cancer Index Composite (EPIC-26) questionnaire (Befort *et al.,* 2005, Sanda *et al.,* 2008). Data will be collected at baseline and 3, 6, 12, 18 and 24 months after completion of radiation therapy.

8.0 STUDY AGENTS- Description and Potential Risks

8.1 AdV-tk injectable

8.1.1 Description

The study agent is AdV-tk, a replication defective adenoviral vector based on the dl309 serotype 5 virus. The vector has a deletion in the E1 region of the adenoviral genome substituted by an Herpes simplex virus thymidine kinase (HSV-tk) expression cassette. The dl309 backbone also has a deletion in the E3 region substituted by non-coding DNA. This vector is identical to the one used in previous clinical studies. Wild-type adenoviruses are a common human virus often associated with cold-like symptoms. Replication-defective means that the virus has been engineered, by deletion of some of its genes, so that it can replicate only in specialized laboratory cells that can complement the deleted genes. The vector is not infectious (it cannot make more viruses) in cells not engineered to be complementing, such as the target tumor cells. The HSV tk gene codes for the Herpes virus TK protein, which is the target of many of the FDA approved

anti-herpetic drugs, such as ganciclovir, acyclovir and valacyclovir. AdV-tk transfers this sensitivity to the target tumor cells.
The vector for this study was prepared for clinical use under current Good Manufacturing Practice (cGMP) conditions for biologics. GMP refers to the Good Manufacturing Practice Regulations promulgated by the US Food and Drug Administration to ensure that products are safe, pure, and effective in what they are purported to do. The product was released with a Certificate of Analysis.

8.1.2 Potential Risks

In previous trials of intratumoral AdV-tk injection followed by prodrug, as described above in the Background section and in the Investigator's Brochure, toxicity was rare and overall the approach has been well tolerated. A summary of grade 3 and 4 toxicities seen in the previous prostate trials is provided in table 2 above. One patient at the highest dose level (2x1012 vp) in the prostate Phase I dose escalation study experienced grade 4 transient thrombocytopenia and also transient grade 3 elevation in liver transaminases (Herman *et al.* 1999). In the ProstAtak® Phase 2a trial, two grade 3 toxicities likely related to standard therapy (transient ALT elevation and GU toxicity), occurred. Overall, the incidence and severity of gastrointestinal and genitourinary toxicity was not significantly different than typically observed with radiation therapy alone. No grade 4 toxicities were observed. Three subjects experienced grade 2 fevers that may have been related to the AdV-tk injection. Fever was less common and lower grade once prophylactic anti-pyretic use was instituted for the injection procedures.
The risks of the prostate injection procedure, whether receiving the AdV-tk or the placebo, are is expected to be lower than the risk of a biopsy procedure, which has a historical risk of significant complication of <1%. The most common minor side effects of prostate biopsy, which are usually self limiting and do not require medical intervention, include hematuria, urinary retention, dysuria, hematospermia, rectal bleeding, fever, infection and pain (Raaijmakers *et al.,* 2002, Sieber *et al.,* 2007, Ecke *et al.,* 2008). Major complications of biopsy range in occurrence from 0.4-4% with most of these being hospitalization and/or treatment for infection, urinary retention or bleeding. Studies evaluating the risk of repeated biopsy did not show increased risk with second biopsies (Djavan *et al.,* 2001).

8.1.3 Potential Biohazard Exposure to Health-Care Personnel and Close Contacts

Personnel Involved in Vector Injection: There is a low potential for biohazard exposure to the personnel involved in vector injection. The viral vector is suspended in a small volume (~2 ml) of buffer and is contained in a glass vial with a rubber stopper. The syringe is loaded from the vial with no vector loss. Intratumoral injection is made and the amount of leakage along the needle track is likely to be minute. Procedures for handling the vector, vector disposal and accidental spills of the vector are described in the Vector Storage and Administration SOP (SOP VS).

Post-Procedural: There is a potential that AdV-tk virus may escape from the patient via urine, feces, saliva, mucus, tears or other excretions. Studies in non-human primates and in cotton rats indicate that the potential is low. In a Phase I dose escalation study in prostate cancer, vector was detected by PCR in urine samples briefly after treatment. However, all cultures of blood and urine were negative for growth of adenovirus. There is also a theoretical possibility that the vector could recombine with wild-type adenovirus, although this has not been detected in

previous studies. If this did occur, infection would be expected to cause a mild, self-limited infection, such as cold-like symptoms, similar to a common adenovirus infection.

8.2 Placebo injectable

The placebo for this study was prepared at the same facility as the AdV-tk vector under current Good Manufacturing Practice (cGMP) conditions for biologics. It consists of the same vector formulation buffer without vector. The product was released with a Certificate of Analysis.

8.3 Valacyclovir

8.3.1 Description

Valacyclovir caplets are an FDA-approved antiherpetic formulation for oral administration. Valacyclovir is a valine-ester of acyclovir, which is quickly converted to acyclovir by first pass intestinal and/or hepatic metabolism. Peak plasma valacyclovir concentrations are generally less than 0.5 mcg/ml at all doses.

8.3.2 Potential Risks

The most common reported adverse events with valacyclovir reported were nausea in 6-15% of patients, headache in 14-35%, vomiting in 1-6%, dizziness or abdominal pain in 2-11%, arthralgia in 1-6% and depression in 1-7%. Laboratory abnormalities such as abnormal liver function tests were observed in 1-4% of patients while anemia, leukopenia, thrombocytopenia and elevated serum creatinine occurred in 1% of patients or less. In addition, the following adverse events have been identified in clinical practice: allergic reactions (anaphylaxis, angioedema, dyspnea, pruritus, rash and urticaria), CNS symptoms (confusion, hallucinations, agitation, aggressive behavior, mania), gastrointestinal (diarrhea), renal failure, erythema multiforme, facial edema, hypertension and tachycardia. A rare but serious adverse event of thrombotic thrombocytopenic purpura/ hemolytic uremic syndrome (TTP/HUS), in some cases resulting in death, has been observed in clinical trials of high dose valacyclovir (8 grams per day) in patients with advanced HIV infection, allogeneic bone marrow transplant and renal transplant recipients.

9.0 STATISTICS

9.1 Study Design

This is a multicenter, randomized, placebo-controlled, double-blind study in newly diagnosed prostate cancer. The aim of the study is to assess the efficacy of AdV-tk + valacyclovir in combination with the standard of care (Test arm) compared to placebo + valacyclovir + standard of care (Control Arm), where the standard of care (SOC) is external beam radiation therapy with or without short-term androgen deprivation therapy (ADT). Eligible patients (per Inclusion/Exclusion criteria) will be randomly assigned onto the test arm or the control arm with a 2:1 randomization ratio. A stratification procedure will be used in the randomization on the variables of NCCN risk category and short-term ADT.

9.2 Primary Endpoints and Sample Size Calculations

The primary endpoint for the study is disease-free survival (DFS), which will be evaluated on the intent to treat (ITT) population. Enrolled at a rate of 10 subjects a month for the first 6 months and 30 patients a month during the next 22 months, 711 patients (474 subjects on the test arm and 237 on the control arm) are expected to provide 98 events within 58 months of the first

patient enrollment to the study. A total sample size of 711 subjects will yield approximately 90% power to detect a 15 percent relative improvement at an alpha level of 0.05 to be statistically significant. The sample size estimation takes into account randomizing in favor of the test arm by a 2:1 ratio and up to 20% dropout rate. This calculation further assumes that, over 58 months of follow-up, there will be a DFS rate of 75% in the control arm and 86.5% in the treatment arm, or equivalently a hazard ratio of 0.5, along with a 4% dropout rate per year on each arm due to loss to follow-up. Patients will be stratified by the variable of ADT (present or absent) and NCCN risk category (intermediate or high risk with only one high risk feature) prior to study arm allocation.

An interim analysis is planned after the first 49 events, giving an information fraction of 0.5. The Lan-DeMets Family (Gordon Lan *et al.,* 1983) was chosen to formalize the error spending function for stopping the trial early to reject the null hypothesis at an alpha level of 0.003. There is 25% estimated probability of rejecting the null at such early stage. The study will not be stopped until all patients have been accrued and treatments received.

The sample size calculations were done using East 5.2 (Cytel, Cambridge, MA).

9.2.1 Disease free survival

The disease-free survival (DFS) is defined as time from randomization to local failure, regional failure, distant metastases or death from any cause. The time to disease-free survival is measured from the date of randomization to the earliest event or to the date of the most recent follow-up if no event occurred. The earliest event date will be defined as the date when the event is confirmed. For example, for a positive DRE detected recurrence, the date of the event will be the date of the confirmatory biopsy if medically possible, otherwise will be the date recurrence considered definitive by the investigator. For a symptomatic patient, the date of confirmatory image or biopsy will be considered the date of the event, even if the patient had missed follow-up visits. Analyses of DFS will be based on the intent to treat population. If patient is lost to follow-up, the data will be censored at the time of last follow-up when patient is event-free. If patient refuses to continue with the protocol every attempt will be made to continue follow-up and analyze as per intent to treat. If they refuse follow-up they will be censored at the last follow-up.

Patients who do not have pre-enrollment CT/MRI and are found to have locally advanced disease on the radiation therapy treatment planning CT/MRI, will continue on study as planned and be evaluated as per intent to treat.
Local failure is defined as:

1. Increase in tumor size by 50% or more as measured by palpation (product of length and width)
2. Reappearance of palpable tumor after clearing
3. Biopsy revealing adenocarcinoma of the prostate at least 2 years after randomization. Prostate biopsy will be performed at 2 years in all subjects except those who already experienced a DFS event.
4. Urinary obstruction secondary to tumor confirmed by biopsy.

Evaluation of a positive DRE or urinary obstruction as the basis for declaration of local failure requires confirmation by prostate biopsy or clear medical justification of why this is not medically possible.

Regional failure is defined as clinical with radiographic evidence of tumor in the pelvis.

Distant metastases are defined as clinical with radiographic evidence of disease beyond the pelvis.

The first site of suspected metastatic disease to the pelvis or distantly will be biopsied, if medically indicated, for confirmation in addition to radiographic and PSA documentation.

The use of adjuvant androgen deprivation therapy (ADT) with radiation therapy is not universally accepted as SOC for the target study population. Thus, the trial has ADT as optional, based on the clinical judgment of the treating physicians. ADT will be used as a stratification criterion and for subset analysis.

The DFS result for the Test Arm is conservatively projected at 86.25% based on the results from the ProstAtak® Phase 2a trial, in which the results were 92% (57/62) for all patients; 94% (29/31) for Arm A (low risk) and 90% (28/31) for Arm B (intermediate-high risk). In that trial, DFS for the intermediate risk group was 95% (20/21) and 80% (8/10) for the high-risk group. The DFS for the control group is estimated at 75% based on data from multiple recent studies described in the Background section.

9.2.2 Statistical and Analytical Plans

A test will be deemed significant if the corresponding p-value is less than or equal to 0.05, unless otherwise specified. All p-values will be 2-sided.

DFS curves will be estimated using the Kaplan-Meier technique (Kaplan *et al.,* 1958). The primary analysis to compare the differences between the treatment arms for this endpoint (duration of DFS) will use the Cox multivariate regression model (Cox 1972) to estimate the hazard of disease free survival between the two treatment arms adjusting for the two stratification factors (ADT use and NCCN risk category) at a level of significance of 5%. Supplementary analyses will be done using a stratified log rank test adjusting for ADT use and disease risk category (Mantel 1966).

9.3 Safety Monitoring Plan

The study will be monitored by a Data and Safety Monitoring Board (DSMB). It will follow established procedures for monitoring toxicity and progress of the trial.

The Acute Toxicity Monitoring period will be from the start of treatment until the end of radiation, which will be 6 weeks after the last AdV-tk injection and 4 weeks after completing the last course of valacyclovir. During this period, investigators will be asked to grade symptoms using the CTCAE ver4.0. Unexpected serious adverse events and symptoms >grade 2 will be reported with expedited reporting to the Sponsor, reviewed by the DSMB and reported to regulatory agencies according to Standard Operating Procedures.

The Late Toxicity Monitoring period will begin after the Acute Toxicity Monitoring period and will continue for 5 years from the end of radiation and after that, long-term follow up will continue as described in 7.1.8 above. Data will be collected on new medical conditions according to categories including Allergy/Rheumatology, Cardiovascular, Endocrine, Hematology/Oncology and Neurologic. Genitourinary and gastrointestinal toxicities, which are expected after prostate radiation therapy, will be graded using the CTCAE ver4.0.

The DSMB will evaluate the safety data at every meeting at least twice per year for potential imbalance in toxicity between the treatment and control groups, based on the number of observed adverse events vs. the number of patients injected. In case of significant safety concern, the Sponsor will be alerted, and the data discussed with the FDA prior to further enrollment.

Overall toxicity profile analyses will be based on all randomized patients who started protocol treatment according to the treatment actually started. Toxicity summary analysis and report will be done at the time of DFS analysis and after long term follow-up.

The Kruskal and Wallis test will be used for toxicity profile comparisons between the two arms while separated into three blinded groups (Kruskal *et al.,* 1952). The proportion of toxicity events between the two arms once unblinded will be compared using the Chi-square test.

9.4 Secondary Endpoints

9.4.1 PSA nadir

A secondary endpoint is PSA nadir. This endpoint will be followed but is not a primary endpoint for the study and thus was not used for sample size determination. Results from the ProstAtak® Phase 2a study were described above in the Background section (section 1.6.1 and Table 3). The proportion of patients with PSA nadir ≤0.2 ng/ml in the ProstAtak® arm will be compared to the control arm using Chi-square test. Logistic regression model will be used to estimate the odds of PSA nadir ≤0.2 ng/ml between the two arms to adjust for other covariates, if needed.

9.4.2 Freedom from biochemical failure, Prostate cancer specific survival and Overall Survival

Biochemical failure will be defined using the PSA nadir plus 2ng/ml definition, as recently recommended by a consensus panel of experts (Thames *et al.,* 2003, Roach *et al.* 2006). Freedom from biochemical failure is defined as the date from randomization to date of the earliest confirmed biochemical failure. Prostate cancer specific survival is defined as the date from randomization to date of death due to prostate cancer. Overall survival is defined as date from randomization to date of death due to all causes. The data will be censored at the time of last follow-up when patient is event-free. If patient refuses to continue with the protocol every attempt will be made to continue follow-up and analyze as per intent to treat. If they refuse follow-up they will be censored at the last follow-up. These endpoints will be followed but are not the primary endpoints for the study and thus were not used for sample size determination. The data analyses will be performed on all randomized patients. The probability distribution of all these endpoints will be estimated using Kaplan-Meier method (Mantel 1966). The

comparison between treatment groups will use a Cox multivariate regression model (Cox 1972) adjusting for stratification factors.

9.4.3 Quality of Life Assessment

Patient-reported Health Related Quality of Life outcomes will be collected using the Expanded Prostate Cancer Index Composite (EPIC-26) (Befort *et al.* 2005, Sanda *et al.* 2008). Data will be collected at baseline and 3, 6, 12, 18 and 24 months after completion of radiation. This is not a primary endpoint for the study and was not used for sample size determination.

Quality of life will be evaluated including all patients entered in the study. All components and single items are scored on categorical scales and will be summarized as a global health/quality of life scale at each time point. All data will be analyzed using descriptive statistics for the subscales and single items for each study group at each of the assessment points. The global health status/QoL scale will be used as an overall measure for QoL. Quality of Life between the two treatment groups will be evaluated in a longitudinal fashion using the longitudinal mixed model (PROC Mixed in SAS). The model will allow the change of QoL in the two groups over time to be investigated.

9.4.4 Exploratory and Subset Analyses

There is some evidence that radiation therapy plus ProstAtak® without ADT may be comparable to radiation therapy plus ADT in patients with defined intermediate risk (unpublished data). There is also evidence that the overall impact of ProstAtak® may vary depending on the patients' specific risk categorization. The study will explore such associations using regression models and Chi-square statistics to generate scientific hypothesis for further investigation. Exploratory subset analysis by geographical region of treatment (country) in addition to the stratification factors will be evaluated using stratified log rank test. Blood and tissue will be collected from patients agreeing to have their samples archived for future immune studies. The specific studies and assays have not yet been determined.

9.5 Randomization Scheme

The study will use a block stratified randomization method for treatment assignment. The randomization and treatment assignment will be done centrally. The treating physician and clinical site personnel, the central pathologist and the patients as well as the Sponsor will all be blinded to treatment assignment.

10.0 ETHICAL AND REGULATORY CONSIDERATIONS

10.1 Consent Procedure

All patients must sign an approved informed consent form in order to be eligible for this study.

The subjects for this study will be identified by the clin site personnel. An investigator at the site will discuss the purpose of the research, the scientific background, the study procedures, the potential side effects and alternative therapies with the patient. The investigator will answer questions and also be available to answer further questions in the future. Patients who indicate interest in the study will review, in detail, the content of the consent form, including procedures, duration of the study, potential side effects, voluntary participation, agreement to a two-year biopsy, etc. Questions will be answered by the research personnel at the site when necessary or

requested by the patient. When a patient decides to participate, they will need to sign and date the informed consent form and meet with the study personnel to begin scheduling the procedures. Study participants will receive a copy of the signed consent form.

10.2 Confidentiality

Confidentiality of patients' records will be maintained within legal limits. The data from this study may be published, however no patients will be named or identified.

10.3 Risk-Benefit Ratio

The present study may have no benefit to the study subjects. However, pre-clinical studies show significant cell killing in cancer tumor models. Phase I and 2 studies in prostate cancer have shown encouraging but not definitive results. The standard therapy for the patients in this proposed study will not be significantly altered by the experimental therapy. Important information may be gained for future patients regarding the efficacy of ProstAtak® with minimal risk expected for the individual patients participating in this protocol.

10.4 Adverse Event Reporting

Adverse events should be reported to the Sponsor, Advantagene, according to the Adverse Event SOP (SOP AE) Adverse events which are unexpected and associated with the use of the investigational product will be reported to the FDA according to the applicable regulations with the timing shown in the schedule below. Reporting to the IRBs and IBCs will be done according to the requirements of the relevant IRBs and IBCs. The principal investigator or his designee will be responsible for reporting to the IRB, IBC, NIH OBA and to the Sponsor. The Sponsor will be responsible for reporting to the FDA. Principal investigators may transfer NIH OBA reporting requirements to the Sponsor by providing the Sponsor with a letter addressed to NIH OBA stating this prior to initiation of the study. The appropriate forms for adverse event reporting will be used for each agency.

Table 9: Safety Reporting

**Type of event**	**Timing of Report***
Fatal or life-threatening	As soon as possible,<br>no later than 7 calendar days
Serious adverse event resulting in one of the following:<br>1. Inpatient hospitalization or prolongation of hospitalization<br>2. A persistent or significant disability or incapacity<br>3. A congenital anomaly or birth defect<br>4. Important medical event that, based on the medical judgment of the investigator, may require medical or surgical intervention to prevent one of the outcomes listed above	As soon as possible,<br>no later than 15 calendar days
Any other unexpected adverse experiences associated with the use of the product	In the annual report

***** Timing is relative to when the person responsible for making the report becomes aware of the event and it is determined that it qualifies for reporting.

11.0 BIBLIOGRAPHY

Agard, C.,C. Ligeza,B. Dupas,A. Izembart,C. El Kouri,P. Moullier, N. Ferry (2001). "Immune-dependent distant bystander effect after adenovirus-mediated suicide gene transfer in a rat model of liver colorectal metastasis." Cancer gene therapy 8(2): 128-136.

Aguilar, L. K.,B. Teh,W. Mai,J. Caillouet,G. Ayala,E. Aguilar-Cordova, E. Butler (2006). "Five year follow up of a phase II study of cytotoxic immunotherapy combined with radiation in newly diagnosed prostate cancer." Journal of Clinical Oncology 24(18_suppl): 4635-4635.

Al-Mamgani, A.,W. L. van Putten,W. D. Heemsbergen,G. J. van Leenders,A. Slot,M. F. Dielwart,L. Incrocci, J. V. Lebesque (2008). "Update of Dutch multicenter dose-escalation trial of radiotherapy for localized prostate cancer." Int J Radiat Oncol Biol Phys 72(4): 980-988.

Al-Mamgani, A.,W. L. J. van Putten,W. D. Heemsbergen,G. J. L. H. van Leenders,A. Slot,M. F. H. Dielwart,L. Incrocci, J. V. Lebesque (2008). "Update of Dutch multicenter dose-escalation trial of radiotherapy for localized prostate cancer." International journal of radiation oncology, biology, physics 72(4): 980-988.

Ayala, G.,T. Satoh,R. Li,M. Shalev,Y. Gdor,E. Aguilar-Cordova,A. Frolov,T. M. Wheeler,B. J. Miles,K. Rauen,B. S. Teh,E. B. Butler,T. C. Thompson, D. Kadmon (2006). "Biological response determinants in HSV-tk + ganciclovir gene therapy for prostate cancer." Molecular therapy : the journal of the American Society of Gene Therapy 13(4): 716-728.

Ayala, G.,T. M. Wheeler,M. Shalev,T. C. Thompson,B. Miles,E. Aguilar-Cordova,S. Chakraborty, D. Kadmon (2000). "Cytopathic effect of in situ gene therapy in prostate cancer." Human pathology 31(7): 866-870.

Barba, D.,J. Hardin,M. Sadelain, F. H. Gage (1994). "Development of anti-tumor immunity following thymidine kinase-mediated killing of experimental brain tumors." Proceedings of the National Academy of Sciences of the United States of America 91(10): 4348-4352.

Befort, C. A.,M. J. Zelefsky,P. T. Scardino,E. Borrayo,R. B. Giesler, M. W. Kattan (2005). "A measure of health-related quality of life among patients with localized prostate cancer: results from ongoing scale development." Clinical prostate cancer 4(2): 100-108.

Bolla, M.,L. Collette,L. Blank,P. Warde,J. B. Dubois,R.-O. Mirimanoff,G. Storme,J. Bernier,A. Kuten,C. Sternberg,J. Mattelaer,J. Lopez Torecilla,J. R. Pfeffer,C. Lino Cutajar,A. Zurlo, M. Pierart (2002). "Long-term results with immediate androgen suppression and external irradiation in patients with locally advanced prostate cancer (an EORTC study): a phase III randomised trial." Lancet 360(9327): 103-106.

Cavallo, F.,P. Signorelli,M. Giovarelli,P. Musiani,A. Modesti,M. J. Brunda,M. P. Colombo, G. Forni (1997). "Antitumor efficacy of adenocarcinoma cells engineered to produce interleukin 12 (IL-12) or other cytokines compared with exogenous IL-12." Journal of the National Cancer Institute 89(14): 1049-1058.

Chhikara, M.,H. Huang,M. T. Vlachaki,X. Zhu,B. Teh,K. J. Chiu,S. Woo,B. Berner,E. O. Smith,K. C. Oberg,L. K. Aguilar,T. C. Thompson,E. B. Butler, E. Aguilar-Cordova (2001). "Enhanced therapeutic effect of HSV-tk+GCV gene therapy and ionizing radiation for prostate cancer." Molecular therapy : the journal of the American Society of Gene Therapy 3(4): 536-542.

Cox, D. R. (1972). "Regression Models and Life-Tables." Journal of the Royal Statistical Society. Series B (Methodological) 34(2): 187-220.

Crook, J.,C. Ludgate,S. Malone,J. Lim,G. Perry,L. Eapen,J. Bowen,S. Robertson, G. Lockwood (2004). "Report of a multicenter Canadian phase III randomized trial of 3 months vs. 8 months neoadjuvant androgen deprivation before standard-dose radiotherapy for clinically localized prostate cancer." International journal of radiation oncology, biology, physics 60(1): 15-23.

Crook, J.,C. Ludgate,S. Malone,G. Perry,L. Eapen,J. Bowen,S. Robertson, G. Lockwood (2009). "Final report of multicenter Canadian Phase III randomized trial of 3 versus 8 months of neoadjuvant androgen deprivation therapy before conventional-dose radiotherapy for clinically localized prostate cancer." International journal of radiation oncology, biology, physics 73(2): 327-333.

Crook, J.,S. Malone,G. Perry,Y. Bahadur,S. Robertson, M. Abdolell (2000). "Postradiotherapy prostate biopsies: what do they really mean? Results for 498 patients." International journal of radiation oncology, biology, physics 48(2): 355-367.

D'Amico, A. V.,M.-H. Chen,A. A. Renshaw,B. Loffredo, P. W. Kantoff (2008). "Risk of prostate cancer recurrence in men treated with radiation alone or in conjunction with combined or less than combined androgen suppression therapy." Journal of clinical oncology : official journal of the American Society of Clinical Oncology 26(18): 2979-2983.

D'Amico, A. V.,M.-H. Chen,A. A. Renshaw,M. Loffredo, P. W. Kantoff (2008). "Androgen suppression and radiation vs radiation alone for prostate cancer: a randomized trial." JAMA : the journal of the American Medical Association 299(3): 289-295.

D'Amico, A. V.,K. Cote,M. Loffredo,A. A. Renshaw, D. Schultz (2003). "Determinants of prostate cancer specific survival following radiation therapy during the prostate specific antigen era." The Journal of urology 170(6 Pt 2): S42-46; discussion S46-47.

D'Amico, A. V.,J. Manola,M. Loffredo,A. A. Renshaw,A. DellaCroce, P. W. Kantoff (2004). "6-month androgen suppression plus radiation therapy vs radiation therapy alone for patients with clinically localized prostate cancer: a randomized controlled trial." JAMA : the journal of the American Medical Association 292(7): 821-827.

D'Amico, A. V.,R. Whittington,S. B. Malkowicz,D. Schultz,K. Blank,G. A. Broderick,J. E. Tomaszewski,A. A. Renshaw,I. Kaplan,C. J. Beard, A. Wein (1998). "Biochemical outcome after radical prostatectomy, external beam radiation therapy, or interstitial

radiation therapy for clinically localized prostate cancer." JAMA : the journal of the American Medical Association 280(11): 969-974.

Dearnaley, D. P.,M. R. Sydes,J. D. Graham,E. G. Aird,D. Bottomley,R. A. Cowan,R. A. Huddart,C. C. Jose,J. H. Matthews,J. Millar,A. R. Moore,R. C. Morgan,J. M. Russell,C. D. Scrase,R. J. Stephens,I. Syndikus, M. K. B. Parmar (2007). "Escalated-dose versus standard-dose conformal radiotherapy in prostate cancer: first results from the MRC RT01 randomised controlled trial." The lancet oncology 8(6): 475-487.

DeWeese, T. L., D. Y. Song (2005). "Radiation dose escalation as treatment for clinically localized prostate cancer: Is more really better?" JAMA 294(10): 1274-1276.

Djavan, B.,M. Waldert,A. Zlotta,P. Dobronski,C. Seitz,M. Remzi,A. Borkowski,C. Schulman, M. Marberger (2001). "Safety and morbidity of first and repeat transrectal ultrasound guided prostate needle biopsies: results of a prospective European prostate cancer detection study." The Journal of urology 166(3): 856-860.

Eade, T. N.,E. M. Horwitz,K. Ruth,M. K. Buyyounouski,D. J. D'Ambrosio,S. J. Feigenberg,D. Y. T. Chen, A. Pollack (2008). "A comparison of acute and chronic toxicity for men with low-risk prostate cancer treated with intensity-modulated radiation therapy or (125)I permanent implant." International journal of radiation oncology, biology, physics 71(2): 338-345.

Eastham, J. a.,S. H. Chen,I. Sehgal,G. Yang,T. L. Timme,S. J. Hall,S. L. Woo, T. C. Thompson (1996). "Prostate cancer gene therapy: herpes simplex virus thymidine kinase gene transduction followed by ganciclovir in mouse and human prostate cancer models." Human gene therapy 7(4): 515-523.

Ecke, T. H.,S. Gunia,P. Bartel,S. Hallmann,S. Koch, J. Ruttloff (2008). "Complications and risk factors of transrectal ultrasound guided needle biopsies of the prostate evaluated by questionnaire." Urologic oncology 26(5): 474-478.

Fujita, T.,B. S. Teh,T. L. Timme,W.-Y. Mai,T. Satoh,N. Kusaka,K. Naruishi,E. A. Fattah,E. Aguilar-Cordova,E. B. Butler, T. C. Thompson (2006). "Sustained long-term immune responses after in situ gene therapy combined with radiotherapy and hormonal therapy in prostate cancer patients." International journal of radiation oncology, biology, physics 65(1): 84-90.

Fyfe, J. A.,P. M. Keller,P. A. Furman,R. L. Miller, G. B. Elion (1978). "Thymidine kinase from herpes simplex virus phosphorylates the new antiviral compound, 9-(2-hydroxyethoxymethyl)guanine." The Journal of biological chemistry 253(24): 8721-8727.

Gagandeep, S.,R. Brew,B. Green,S. E. Christmas,D. Klatzmann,G. J. Poston, A. R. Kinsella (1996). "Prodrug-activated gene therapy: involvement of an immunological component in the "bystander effect"." Cancer gene therapy 3(2): 83-88.

Gordon Lan, K. K., D. L. Demets (1983). "Discrete sequential boundaries for clinical trials." Biometrika 70(3): 659-663.

Hall, S. J.,M. A. Sanford,G. Atkinson, S. H. Chen (1998). "Induction of potent antitumor natural killer cell activity by herpes simplex virus-thymidine kinase and ganciclovir therapy in an orthotopic mouse model of prostate cancer." Cancer research 58(15): 3221-3225.

Han, M.,A. W. Partin,M. Zahurak,S. Piantadosi,J. I. Epstein, P. C. Walsh (2003). "Biochemical (prostate specific antigen) recurrence probability following radical prostatectomy for clinically localized prostate cancer." The Journal of urology 169(2): 517-523.

Hanks, G. E.,T. F. Pajak,A. Porter,D. Grignon,H. Brereton,V. Venkatesan,E. M. Horwitz,C. Lawton,S. A. Rosenthal,H. M. Sandler, W. U. Shipley (2003). "Phase III trial of long-term adjuvant androgen deprivation after neoadjuvant hormonal cytoreduction and radiotherapy in locally advanced carcinoma of the prostate: the Radiation Therapy Oncology Group Protocol 92-02." Journal of clinical oncology : official journal of the American Society of Clinical Oncology 21(21): 3972-3978.

Hasenburg, A.,X. W. Tong,D. C. Fischer,A. Rojas-Martinez,C. Nyberg-Hoffman,A. L. Kaplan,R. H. Kaufman,I. Ramzy,E. Aguilar-Cordova, D. G. Kieback (2001). "Adenovirus-mediated thymidine kinase gene therapy in combination with topotecan for patients with recurrent ovarian cancer: 2.5-year follow-up." Gynecologic oncology 83(3): 549-554.

Hasenburg, A.,X. W. Tong,A. Rojas-Martinez,C. Nyberg-Hoffman,C. C. Kieback,A. Kaplan,R. H. Kaufman,I. Ramzy,E. Aguilar-Cordova, D. G. Kieback (2000). "Thymidine kinase gene therapy with concomitant topotecan chemotherapy for recurrent ovarian cancer." Cancer gene therapy 7(6): 839-844.

Hasenburg, A.,X. W. Tong,A. Rojas-Martinez,C. Nyberg-Hoffman,C. C. Kieback,A. L. Kaplan,R. H. Kaufman,I. Ramzy,E. Aguilar-Cordova, D. G. Kieback (1999). "Thymidine kinase (TK) gene therapy of solid tumors: valacyclovir facilitates outpatient treatment." Anticancer research 19(3B): 2163-2165.

Herman, J. R.,H. L. Adler,E. Aguilar-Cordova,A. Rojas-Martinez,S. Woo,T. L. Timme,T. M. Wheeler,T. C. Thompson, P. T. Scardino (1999). "In situ gene therapy for adenocarcinoma of the prostate: a phase I clinical trial." Human gene therapy 10(7): 1239-1249.

Hurwitz, M. D.,L. Schnieder,J. Manola,C. J. Beard,I. D. Kaplan, A. V. D'Amico (2002). "Lack of radiation dose response for patients with low-risk clinically localized prostate cancer: a retrospective analysis." International journal of radiation oncology, biology, physics 53(5): 1106-1110.

Immonen, A.,M. Vapalahti,K. Tyynelä,H. Hurskainen,A. Sandmair,R. Vanninen,G. Langford,N. Murray, S. Ylä-Herttuala (2004). "AdvHSV-tk gene therapy with intravenous ganciclovir

improves survival in human malignant glioma: a randomised, controlled study." Molecular therapy : the journal of the American Society of Gene Therapy 10(5): 967-972.

Kaplan, E. L., P. Meier (1958). "Nonparametric estimation from incomplete observations." Journal of the American Statistical Association 53: 447-457.

Kruskal, W. H., W. A. Wallis (1952). "Use of Ranks in One-Criterion Variance Analysis." Journal of the American Statistical Association 47(260): 583-621.

Kuban, D. A.,H. D. Thames,L. B. Levy,E. M. Horwitz,P. A. Kupelian,A. A. Martinez,J. M. Michalski,T. M. Pisansky,H. M. Sandler,W. U. Shipley,M. J. Zelefsky, A. L. Zietman (2003). "Long-term multi-institutional analysis of stage T1-T2 prostate cancer treated with radiotherapy in the PSA era." International journal of radiation oncology, biology, physics 57(4): 915-928.

Kuban, D. A.,S. L. Tucker,L. Dong,G. Starkschall,E. H. Huang,M. R. Cheung,A. K. Lee, A. Pollack (2008). "Long-term results of the M. D. Anderson randomized dose-escalation trial for prostate cancer." International journal of radiation oncology, biology, physics 70(1): 67-74.

Kupelian, P.,D. Kuban,H. Thames,L. Levy,E. Horwitz,A. Martinez,J. Michalski,T. Pisansky,H. Sandler,W. Shipley,M. Zelefsky, A. Zietman (2005). "Improved biochemical relapse-free survival with increased external radiation doses in patients with localized prostate cancer: the combined experience of nine institutions in patients treated in 1994 and 1995." International journal of radiation oncology, biology, physics 61(2): 415-419.

Lawton, C. A.,K. Winter,K. Murray,M. Machtay,J. B. Mesic,G. E. Hanks,C. T. Coughlin, M. V. Pilepich (2001). "Updated results of the phase III Radiation Therapy Oncology Group (RTOG) trial 85-31 evaluating the potential benefit of androgen suppression following standard radiation therapy for unfavorable prognosis carcinoma of the prostate." International journal of radiation oncology, biology, physics 49(4): 937-946.

Mantel, N. (1966). "Evaluation of survival data and two new rank order statistics arising in its consideration." Cancer chemotherapy reports. Part 1 50(3): 163-170.

Miles, B. J.,M. Shalev,E. Aguilar-Cordova,T. L. Timme,H. M. Lee,G. Yang,H. L. Adler,K. Kernen,C. K. Pramudji,T. Satoh,Y. Gdor,C. Ren,G. Ayala,T. M. Wheeler,E. B. Butler,D. Kadmon, T. C. Thompson (2001). "Prostate-specific antigen response and systemic T cell activation after in situ gene therapy in prostate cancer patients failing radiotherapy." Human gene therapy 12(16): 1955-1967.

Miles, B. J.,M. Shalev,E. Aguilar-Cordova,T. L. Timme,H. M. Lee,G. Yang,H. L. Adler,K. Kernen,C. K. Pramudji,T. Satoh,Y. Gdor,C. Ren,G. Ayala,T. M. Wheeler,E. B. Butler,D. Kadmon, T. C. Thompson (2001). "Prostate-specific antigen response and systemic T cell activation after in situ gene therapy in prostate cancer patients failing radiotherapy." Hum Gene Ther 12(16): 1955-1967.

Moul, J. W. (2000). "Prostate specific antigen only progression of prostate cancer." The Journal of urology 163(6): 1632-1642.

Nguyen, P. L.,A. Trofimov, A. L. Zietman (2008). "Proton-beam vs intensity-modulated radiation therapy. Which is best for treating prostate cancer?" Oncology (Williston Park, N.Y.) 22(7): 748-754; discussion 754, 757.

Nichol, A.,P. Chung,G. Lockwood,T. Rosewall,L. Divanbiegi,J. Sweet,A. Toi,A. Bayley,R. Bristow,J. Crook,M. Gospodarowicz,M. McLean,M. Milosevic,P. Warde, C. Catton (2005). "A phase II study of localized prostate cancer treated to 75.6 Gy with 3D conformal radiotherapy." Radiotherapy and Oncology 76(1): 11-17.

Peeters, S. T. H.,W. D. Heemsbergen,P. C. M. Koper,W. L. J. van Putten,A. Slot,M. F. H. Dielwart,J. M. G. Bonfrer,L. Incrocci, J. V. Lebesque (2006). "Dose-response in radiotherapy for localized prostate cancer: results of the Dutch multicenter randomized phase III trial comparing 68 Gy of radiotherapy with 78 Gy." Journal of clinical oncology : official journal of the American Society of Clinical Oncology 24(13): 1990-1996.

Penson, D. F., M. S. Litwin (2003). "The physical burden of prostate cancer." The Urologic clinics of North America 30(2): 305-313.

Pilepich, M. V.,K. Winter,M. J. John,J. B. Mesic,W. Sause,P. Rubin,C. Lawton,M. Machtay, D. Grignon (2001). "Phase III radiation therapy oncology group (RTOG) trial 86-10 of androgen deprivation adjuvant to definitive radiotherapy in locally advanced carcinoma of the prostate." International journal of radiation oncology, biology, physics 50(5): 1243-1252.

Pollack, A.,A. L. Hanlon,E. M. Horwitz,S. J. Feigenberg,R. G. Uzzo, G. E. Hanks (2004). "Prostate cancer radiotherapy dose response: an update of the fox chase experience." The Journal of urology 171(3): 1132-1136.

Pollack, A.,G. K. Zagars,J. A. Antolak,D. A. Kuban, I. I. Rosen (2002). "Prostate biopsy status and PSA nadir level as early surrogates for treatment failure: analysis of a prostate cancer randomized radiation dose escalation trial." International journal of radiation oncology, biology, physics 54(3): 677-685.

Pollack, A.,G. K. Zagars,G. Starkschall,J. A. Antolak,J. J. Lee,E. Huang,A. C. von Eschenbach,D. A. Kuban, I. Rosen (2002). "Prostate cancer radiation dose response: results of the M. D. Anderson phase III randomized trial." International journal of radiation oncology, biology, physics 53(5): 1097-1105.

Raaijmakers, R.,W. J. Kirkels,M. J. Roobol,M. F. Wildhagen, F. H. Schrder (2002). "Complication rates and risk factors of 5802 transrectal ultrasound-guided sextant biopsies of the prostate within a population-based screening program." Urology 60(5): 826-830.

Raper, S. E.,M. Yudkoff,N. Chirmule,G.-P. Gao,F. Nunes,Z. J. Haskal,E. E. Furth,K. J. Propert,M. B. Robinson,S. Magosin,H. Simoes,L. Speicher,J. Hughes,J. Tazelaar,N. A. Wivel,J. M. Wilson, M. L. Batshaw (2002). "A pilot study of in vivo liver-directed gene transfer with an adenoviral vector in partial ornithine transcarbamylase deficiency." Human gene therapy 13(1): 163-175.

Ray, M. E.,H. D. Thames,L. B. Levy,E. M. Horwitz,P. A. Kupelian,A. A. Martinez,J. M. Michalski,T. M. Pisansky,W. U. Shipley,M. J. Zelefsky,A. L. Zietman, D. A. Kuban (2006). "PSA nadir predicts biochemical and distant failures after external beam radiotherapy for prostate cancer: a multi-institutional analysis." International journal of radiation oncology, biology, physics 64(4): 1140-1150.

Roach, M., 3rd,J. Lu,M. V. Pilepich,S. O. Asbell,M. Mohiuddin,R. Terry,D. Grignon,C. Lawton,W. Shipley,J. Cox, M. Mohuidden (2000). "Predicting long-term survival, and the need for hormonal therapy: a meta-analysis of RTOG prostate cancer trials." International journal of radiation oncology, biology, physics 47(3): 617-627.

Roach, M.,G. Hanks,H. Thames,P. Schellhammer,W. U. Shipley,G. H. Sokol, H. Sandler (2006). "Defining biochemical failure following radiotherapy with or without hormonal therapy in men with clinically localized prostate cancer: recommendations of the RTOG-ASTRO Phoenix Consensus Conference." International journal of radiation oncology, biology, physics 65(4): 965-974.

Roach, M.,J. Lu,M. V. Pilepich,S. O. Asbell,M. Mohiuddin,R. Terry,D. Grignon, M. Mohuidden (2000). "Four prognostic groups predict long-term survival from prostate cancer following radiotherapy alone on Radiation Therapy Oncology Group clinical trials." International journal of radiation oncology, biology, physics 47(3): 609-615.

Rojas-Martínez, A.,A. G. Manzanera,S. W. Sukin,J. Esteban-María,J. F. González-Guerrero,L. Gomez-Guerra,R. Garza-Guajardo,J. P. Flores-Gutiérrez,G. Elizondo Riojas,I. Delgado-Enciso,R. Ortiz-López,L. K. Aguilar,E. B. Butler,H. A. Barrera-Saldaña, E. Aguilar-Cordova (2013). "Intraprostatic distribution and long-term follow-up after AdV-tk immunotherapy as neoadjuvant to surgery in patients with prostate cancer." Cancer gene therapy 20(11): 642-649.

Sanda, M. G.,R. L. Dunn,J. Michalski,H. M. Sandler,L. Northouse,L. Hembroff,X. Lin,T. K. Greenfield,M. S. Litwin,C. S. Saigal,A. Mahadevan,E. Klein,A. Kibel,L. L. Pisters,D. Kuban,I. Kaplan,D. Wood,J. Ciezki,N. Shah, J. T. Wei (2008). "Quality of Life and Satisfaction with Outcome among Prostate-Cancer Survivors." New England Journal of Medicine 358(12): 1250-1261.

Satoh, T.,B. S. Teh,T. L. Timme,W.-Y. Mai,Y. Gdor,N. Kusaka,T. Fujita,C. K. Pramudji,M. T. Vlachaki,G. Ayala,T. Wheeler,R. Amato,B. J. Miles,D. Kadmon,E. B. Butler, T. C. Thompson (2004). "Enhanced systemic T-cell activation after in situ gene therapy with radiotherapy in prostate cancer patients." International journal of radiation oncology, biology, physics 59(2): 562-571.

Scherr, D.,P. W. Swindle, P. T. Scardino (2003). "National Comprehensive Cancer Network guidelines for the management of prostate cancer." Urology 61(2 Suppl 1): 14-24.

Schnell, M. A.,Y. Zhang,J. Tazelaar,G. P. Gao,Q. C. Yu,R. Qian,S. J. Chen,A. N. Varnavski,C. LeClair,S. E. Raper, J. M. Wilson (2001). "Activation of innate immunity in nonhuman primates following intraportal administration of adenoviral vectors." Molecular therapy : the journal of the American Society of Gene Therapy 3(5 Pt 1): 708-722.

Shalev, M.,D. Kadmon,B. S. Teh,E. B. Butler,E. Aguilar-Cordova,T. C. Thompson,J. R. Herman,H. L. Adler,P. T. Scardino, B. J. Miles (2000). "Suicide gene therapy toxicity after multiple and repeat injections in patients with localized prostate cancer." The Journal of urology 163(6): 1747-1750.

Shewach, D. S.,L. K. Zerbe,T. L. Hughes,B. J. Roessler,X. O. Breakefield, B. L. Davidson (1994). "Enhanced cytotoxicity of antiviral drugs mediated by adenovirus directed transfer of the herpes simplex virus thymidine kinase gene in rat glioma cells." Cancer gene therapy 1(2): 107-112.

Shipley, W. U.,H. D. Thames,H. M. Sandler,G. E. Hanks,A. L. Zietman,C. A. Perez,D. A. Kuban,S. L. Hancock, C. D. Smith (1999). "Radiation therapy for clinically localized prostate cancer: a multi-institutional pooled analysis." JAMA : the journal of the American Medical Association 281(17): 1598-1604.

Shipley, W. U.,L. J. Verhey,J. E. Munzenrider,H. D. Suit,M. M. Urie,P. L. McManus,R. H. Young,J. W. Shipley,A. L. Zietman, P. J. Biggs (1995). "Advanced prostate cancer: the results of a randomized comparative trial of high dose irradiation boosting with conformal protons compared with conventional dose irradiation using photons alone." International journal of radiation oncology, biology, physics 32(1): 3-12.

Sieber, P. R.,F. M. Rommel,C. G. Theodoran,R. D. Hong, M. a. Del Terzo (2007). "Contemporary prostate biopsy complication rates in community-based urology practice." Urology 70(3): 498-500.

Sterman, D. H.,A. Recio,A. Vachani,J. Sun,L. Cheung,P. DeLong,K. M. Amin,L. A. Litzky,J. M. Wilson,L. R. Kaiser, S. M. Albelda (2005). "Long-term follow-up of patients with malignant pleural mesothelioma receiving high-dose adenovirus herpes simplex thymidine kinase/ganciclovir suicide gene therapy." Clinical cancer research : an official journal of the American Association for Cancer Research 11(20): 7444-7453.

Sukin, S. W.,M. Chhikara,X. Zhu,G. Ayala,L. K. Aguilar,E. O'Brian Smith,B. J. Miles,T. C. Thompson,D. Kadmon, E. Aguilar-Cordova (2001). "In vivo surgical resection plus adjuvant gene therapy in the treatment of mammary and prostate cancer." Molecular therapy : the journal of the American Society of Gene Therapy 3(4): 500-506.

Teh, B. S.,E. Aguilar-Cordova,K. Kernen,C. C. Chou,M. Shalev,M. T. Vlachaki,B. Miles,D. Kadmon,W. Y. Mai,J. Caillouet,M. Davis,G. Ayala,T. Wheeler,J. Brady,L. S. Carpenter,H. H. Lu,J. K. Chiu,S. Y. Woo,T. Thompson, E. B. Butler (2001). "Phase I/II

trial evaluating combined radiotherapy and in situ gene therapy with or without hormonal therapy in the treatment of prostate cancer--a preliminary report." International journal of radiation oncology, biology, physics 51(3): 605-613.

Teh, B. S.,E. Aguilar-Cordova,M. T. Vlachaki,L. Aguilar,W.-Y. Mai,J. Caillouet,M. Davis,B. Miles,D. Kadmon,G. Ayala,H. H. Lu,J. K. Chiu,L. S. Carpenter,S. Y. Woo,W. H. Grant,T. Wheeler,T. C. Thompson, E. B. Butler (2002). "Combining radiotherapy with gene therapy (from the bench to the bedside): a novel treatment strategy for prostate cancer." The oncologist 7(5): 458-466.

Teh, B. S.,G. Ayala,L. Aguilar,W.-Y. Mai,T. L. Timme,M. T. Vlachaki,B. Miles,D. Kadmon,T. Wheeler,J. Caillouet,M. Davis,L. S. Carpenter,H. H. Lu,J. K. Chiu,S. Y. Woo,T. Thompson,E. Aguilar-Cordova, E. B. Butler (2004). "Phase I-II trial evaluating combined intensity-modulated radiotherapy and in situ gene therapy with or without hormonal therapy in treatment of prostate cancer-interim report on PSA response and biopsy data." International journal of radiation oncology, biology, physics 58(5): 1520-1529.

Tetzlaff, M. T.,B. S. Teh,T. L. Timme,T. Fujita,T. Satoh,K.-I. Tabata,W.-Y. Mai,M. T. Vlachaki,R. J. Amato,D. Kadmon,B. J. Miles,G. Ayala,T. M. Wheeler,E. Aguilar-Cordova,T. C. Thompson, E. B. Butler (2006). "Expanding the therapeutic index of radiation therapy by combining in situ gene therapy in the treatment of prostate cancer." Technology in cancer research & treatment 5(1): 23-36.

Thames, H.,D. Kuban,L. Levy,E. M. Horwitz,P. Kupelian,A. Martinez,J. Michalski,T. Pisansky,H. Sandler,W. Shipley,M. Zelefsky, A. Zietman (2003). "Comparison of alternative biochemical failure definitions based on clinical outcome in 4839 prostate cancer patients treated by external beam radiotherapy between 1986 and 1995." International journal of radiation oncology, biology, physics 57(4): 929-943.

Tong, X. W.,D. G. Engehausen,R. H. Kaufman,I. Agoulnik,C. Contant,C. T. F. Freund,M. K. Oehler,T. E. Kim,A. Hasenburg,S. L. C. Woo, D. G. Kieback (1998). "Improvement of gene therapy for ovarian cancer by using acyclovir instead of ganciclovir in adenovirus mediated thymidine kinase gene therapy." Anticancer Research 18(2 A): 713-718.

Trask, T. W.,R. P. Trask,E. Aguilar-Cordova,H. D. Shine,P. R. Wyde,J. C. Goodman,W. J. Hamilton,A. Rojas-Martinez,S. H. Chen,S. L. Woo, R. G. Grossman (2000). "Phase I study of adenoviral delivery of the HSV-tk gene and ganciclovir administration in patients with current malignant brain tumors." Molecular therapy : the journal of the American Society of Gene Therapy 1(2): 195-203.

Van-der-Linden, R. R. M.,B. L. Haagmans,P. Mongiat-Artus,G. J. van Doornum,R. Kraaij,D. Kadmon,E. Aguilar-Cordova,A. D. M. E. Osterhaus,T. H. van der Kwast,C. H. Bangma, R. R. M. Van-der-Linden (2005). "Virus specific immune responses after human neoadjuvant adenovirus-mediated suicide gene therapy for prostate cancer." European urology 48(1): 153-161.

Vance, W.,S. L. Tucker,R. de Crevoisier,D. A. Kuban, M. R. Cheung (2007). "The predictive value of 2-year posttreatment biopsy after prostate cancer radiotherapy for eventual biochemical outcome." International journal of radiation oncology, biology, physics 67(3): 828-833.

Vile, R. G.,S. Castleden,J. Marshall,R. Camplejohn,C. Upton, H. Chong (1997). "Generation of an anti-tumour immune response in a non-immunogenic tumour: HSVtk killing in vivo stimulates a mononuclear cell infiltrate and a Th1-like profile of intratumoural cytokine expression." International journal of cancer. Journal international du cancer 71(2): 267-274.

Vile, R. G.,J. A. Nelson,S. Castleden,H. Chong, I. R. Hart (1994). "Systemic gene therapy of murine melanoma using tissue specific expression of the HSVtk gene involves an immune component." Cancer research 54(23): 6228-6234.

Vlachaki, M. T.,M. Chhikara,L. Aguilar,X. Zhu,K. J. Chiu,S. Woo,B. S. Teh,T. C. Thompson,E. B. Butler, E. Aguilar-Cordova (2001). "Enhanced therapeutic effect of multiple injections of HSV-TK + GCV gene therapy in combination with ionizing radiation in a mouse mammary tumor model." International journal of radiation oncology, biology, physics 51(4): 1008-1017.

Weller, S.,M. R. Blum,M. Doucette,T. Burnette,D. M. Cederberg,P. de Miranda, M. L. Smiley (1993). "Pharmacokinetics of the acyclovir pro-drug valaciclovir after escalating single- and multiple-dose administration to normal volunteers." Clinical pharmacology and therapeutics 54(6): 595-605.

Zelefsky, M. J.,H. Chan,M. Hunt,Y. Yamada,A. M. Shippy, H. Amols (2006). "Long-term outcome of high dose intensity modulated radiation therapy for patients with clinically localized prostate cancer." The Journal of urology 176(4 Pt 1): 1415-1419.

Zelefsky, M. J.,Z. Fuks,M. Hunt,H. J. Lee,D. Lombardi,C. C. Ling,V. E. Reuter,E. S. Venkatraman, S. A. Leibel (2001). "High dose radiation delivered by intensity modulated conformal radiotherapy improves the outcome of localized prostate cancer." The Journal of urology 166(3): 876-881.

Zelefsky, M. J.,Z. Fuks,M. Hunt,Y. Yamada,C. Marion,C. C. Ling,H. Amols,E. S. Venkatraman, S. A. Leibel (2002). "High-dose intensity modulated radiation therapy for prostate cancer: early toxicity and biochemical outcome in 772 patients." International journal of radiation oncology, biology, physics 53(5): 1111-1116.

Zelefsky, M. J.,V. E. Reuter,Z. Fuks,P. Scardino, A. Shippy (2008). "Influence of local tumor control on distant metastases and cancer related mortality after external beam radiotherapy for prostate cancer." The Journal of urology 179(4): 1368-1373; discussion 1373.

Zietman, A. L. (2002). "Dose escalation in localized prostate cancer: make no assumptions." International journal of radiation oncology, biology, physics 54(3): 635-636.

Zietman, A. L.,M. L. DeSilvio,J. D. Slater,C. J. Rossi,D. W. Miller,J. A. Adams, W. U. Shipley (2005). "Comparison of conventional-dose vs high-dose conformal radiation therapy in clinically localized adenocarcinoma of the prostate: a randomized controlled trial." JAMA : the journal of the American Medical Association 294(10): 1233-1239.

STATISTICS ANALYSIS PLAN FOR PROTOCOL PRTK03 (IND 8437)

A RANDOMIZED CONTROLLED TRIAL OF PROSTATAK™ AS ADJUVANT TO UP-FRONT RADIATION THERAPY FOR LOCALIZED PROSTATE CANCER

Portions of this plan are incorporated in the protocol as Section 9- Statistics

1.0 STATISTICS

1.1 Study Design

This is a multicenter, randomized, placebo-controlled, double-blind study in newly diagnosed prostate cancer. The aim of the study is to assess the efficacy of AdV-tk + valacyclovir in combination with the standard of care (Test arm) compared to placebo + valacyclovir with standard of care (Control Arm), where the standard of care (SOC) is external beam radiation therapy with or without short-term androgen deprivation therapy (ADT). Eligible patients (per Inclusion/Exclusion criteria) will be randomly assigned onto the test arm or the control arm with a 2:1 randomization ratio. A stratification procedure will be used in the randomization on the variables of NCCN risk category and short-term ADT.

1.2 Primary Endpoints and Sample Size Calculations

The primary endpoint for the study is disease-free survival (DFS), which will be evaluated on the intent to treat (ITT) population. Enrolled at a rate of 10 subjects a month for the first 6 months and 30 patients a month during the next 22 months, 711 patients (474 subjects on the test arm and 237 on the control arm) are expected to provide 98 events within 58 months of the first patient enrollment to the study. A total sample size of 711 subjects will yield approximately 90% power to detect a 15 percent relative improvement at an overall alpha level of 0.05 to be statistically significant (0.003 at interim analysis and 0.047 at final analysis). The sample size estimation takes into account randomizing in favor of the test arm by a 2:1 ratio and up to 20% dropout rate. This calculation further assumes that, over 58 months of follow-up, there will be a DFS rate of 75% in the control arm and 86.5% in the treatment arm, or equivalently a hazard ratio of 0.5, along with a 4% dropout rate per year on each arm due to loss to follow-up. Patients will be stratified by the variable of ADT (present or absent) and NCCN risk category (intermediate or high risk with only one high risk feature) prior to study arm allocation.

An interim analysis is planned after the first 49 events, giving an information fraction of 0.5. The Lan-DeMets Family (Lan-DeMets (1983)) was chosen to formalize the error spending function for stopping the trial early to reject the null hypothesis at an alpha level of 0.003. There is 25% estimated probability of rejecting the null at such early stage.

The power calculations were done using East 5.2 (Cytel, Cambridge, MA).

The following selections were used for study power calculations:

Survival end point for superiority trial with two samples.

Logrank test given accrual duration and study duration.

The study was designed as two sided with a Significance Level of 0.05 and planned for a sample size of 711 patients. The sample size was based on previous protocol calculations.
Two equally spaced looks using Published Spending Functions (Pub) are planned.
The treatment fraction is 0.667, at a 2:1 randomization rate.
The null hypothesis is to be rejected.
The spending function boundary is symmetric and the Alpha spending function used is Lan-DeMets with OF parameter (conservative boundaries similar to O'Brien-Fleming).

Accrual information: Subjects are followed until end of study. Accrual duration is calculated at 28 months, based on a planned accrual of 10 patients per month for the first six months (60 patients accrued, 8.5% of total), and 30 patients per month for the remainder of the accrual period (22 additional months to accrue 711 patients, 100%). The study duration is planned for

30 months after last accrual, thus 58 months total. The accrual has two periods inputted as follows:

Period	At	% Accrued
1	6	8.5
2	28	100

Survival information: 6-hazard pieces were inputted using % survival rates with an overall hazard ratio of 0.5.
The cumulative survival rates were inputted as detailed in the table below with the rationale explained in the comment column on the right.

Period #	At (Months since randomization)	Cumulative % DFS in Control	Cumulative % DFS in Treatment*	Comment
1	10	99	99.5	Few recurrences immediately after radiation.
2	20	95	97.5	Few recurrence in short time frame. Clinical surveillance only.
3	30	80	89.4	Start of critical time frame for recurrence. Clinical and active histological evaluation.
4	40	79	88.9	Clinical surveillance only after active histological evaluation.
5	50	77	87.7	Clinical surveillance only.
6	58	75	86.6	Clinical surveillance only.

*Automatically calculated by East

Dropout information: Dropout rates were back-calculated based on an estimated total of 20% dropout per group at a rate of approximately 4% per year. The input method was dropout rates. Five periods were defined with months as the unit of time. The input table was as follows:

Period #	At (Months since randomization)	Cumulative % dropouts in Control	Cumulative % dropouts in Treatment
1	12	4	4
2	24	8	8
3	36	12	12
4	48	16	16
5	58	20	20

The log-hazard variance was set to Null.

This design generated a Power of 0.896.

1.2.1 Disease free survival

The disease-free survival (DFS) is defined as time from randomization to local failure, regional failure, distant metastases or death from any cause. The time to disease-free survival is

measured from the date of randomization to the earliest event or to the date of the most recent follow-up if no event occurred. The earliest event date will be defined as the date when the event is confirmed. For example, for a positive DRE detected recurrence, the date of the event will be the date of the confirmatory biopsy if medically possible, otherwise will be the date recurrence considered definitive by the investigator. For a symptomatic patient, the date of confirmatory image or biopsy will be considered the date of the event, even if the patient had missed follow-up visits. See Appendix 1 for additional details on handling of missing data and dating of events in specific sample scenarios. Analyses of DFS will be based on the intent to treat population. If patient is lost to follow-up, the data will be censored at the time of last follow-up when patient is event-free. If patient refuses to continue with the protocol every attempt will be made to continue follow-up and analyze as per intent to treat. If they refuse follow-up they will be censored at the last follow-up.

Patients who do not have pre-enrollment CT/MRI and are found to have locally advanced disease on the radiation therapy treatment planning CT/MRI, will continue on study as planned and be evaluated as per intent to treat.

Local failure is defined as:

1. Increase in tumor size by 50% or more as measured by palpation (product of length and width)
2. Reappearance of palpable tumor after clearing
3. Biopsy revealing adenocarcinoma of the prostate at least 2 years after randomization. Prostate biopsy will be performed at 2 years in all subjects except those who already experienced a DFS event.
4. Urinary obstruction secondary to tumor confirmed by biopsy.

Evaluation of a positive DRE or urinary obstruction as the basis for declaration of local failure requires confirmation by prostate biopsy or clear medical justification of why this is not medically possible.

Regional failure is defined as clinical with radiographic evidence of tumor in the pelvis.

Distant metastases are defined as clinical with radiographic evidence of disease beyond the pelvis.

The first site of suspected metastatic disease to the pelvis or distantly will be biopsied, if medically indicated, for confirmation in addition to radiographic and PSA documentation.

The use of adjuvant androgen deprivation therapy (ADT) with radiation therapy is not universally accepted as SOC for the target study population. Thus, the trial has ADT as optional, based on the clinical judgment of the treating physicians. ADT will be used as a stratification criterion and for subset analysis.

The DFS result for the Test Arm is conservatively projected at 86.25% based on the results from the ProstAtak™ Phase 2a trial, in which the results were 92% (57/62) for all patients; 94% (29/31) for Arm A (low risk) and 90% (28/31) for Arm B (intermediate-high risk). In that trial, DFS for the intermediate risk group was 95% (20/21) and 80% (8/10) for the high-risk group.

The DFS for the control group is estimated at 75% based on data from multiple recent studies described in the Background section.

1.2.2 Statistical and Analytical Plans

A test will be deemed significant if the corresponding p-value is less than or equal to 0.05, unless otherwise specified. All p-values will be 2-sided.

DFS curves will be estimated using the Kaplan-Meier technique (Kaplan et al. 1958). The primary analysis to compare the differences between the treatment arms for this endpoint (duration of DFS) will use the Cox multivariate regression model (Cox 1972) to estimate the hazard of disease free survival between the two treatment arms adjusting for the two stratification factors (ADT use and NCCN risk category) at an overall level of significance of 5%. Supplementary analysis will be done using a stratified log rank test adjusting for ADT use and disease risk category (Mantel 1966).

The primary analyses will be tested for robustness by exploratory sensitivity analyses adjusting the assumptions as described in Appendix 2.

1.3 Safety Monitoring Plan

The study will be monitored by a Data and Safety Monitoring Board (DSMB). It will follow established procedures for monitoring toxicity and progress of the trial.

The Acute Toxicity Monitoring period will be from the start of treatment until the end of radiation, which will be 6 weeks after the last AdV-tk injection and 4 weeks after completing the last course of valacyclovir. During this period, investigators will be asked to grade symptoms using the CTCAE ver4.0. Unexpected serious adverse events and symptoms >grade 2 will be reported with expedited reporting to the Sponsor, reviewed by the DSMB and reported to regulatory agencies according to Standard Operating Procedures.

The Late Toxicity Monitoring period will begin after the Acute Toxicity Monitoring period and will continue for 5 years from the end of radiation and after that, long-term follow up will continue as described in section 7.1.8 of the protocol. Data will be collected on new medical conditions according to categories including Allergy/Rheumatology, Cardiovascular, Endocrine, Hematology/Oncology and Neurologic. Genitourinary and gastrointestinal toxicities, which are expected after prostate radiation therapy, will be graded using the CTCAE ver4.0.

The DSMB will evaluate the safety data at every meeting at least twice per year for potential imbalance in toxicity between the treatment and control groups, based on the number of observed adverse events vs. the number of patients injected. In case of significant safety concern, the Sponsor will be alerted and the data discussed with the FDA prior to further enrollment.

Overall toxicity profile analyses will be based on all randomized patients who started protocol treatment according to the treatment actually started. Toxicity summary analysis and report will be done at the time of DFS analysis and after long term follow-up.

The Kruskal and Wallis test will be used for toxicity profile comparisons between the two arms while separated into three blinded groups (Kruskal et al. 1952). The proportion of toxicity events between the two arms once unblinded will be compared using the Chi-square test.

1.4 Secondary Endpoints

1.4.1 PSA nadir

A secondary endpoint is PSA nadir. This endpoint will be followed, but is not a primary endpoint for the study and thus was not used for sample size determination. Results from the ProstAtak™ Phase 2a study were described above in the Background section (section 1.6.1 and Table 2). The proportion of patients with PSA nadir ≤0.2 ng/ml in the ProstAtak™ arm will be compared to the control arm using Chi-square test. Logistic regression model will be used to estimate the odds of PSA nadir ≤0.2 ng/ml between the two arms to adjust for other covariates, if needed.

1.4.2 Freedom from biochemical failure, Prostate cancer specific survival and Overall Survival

Biochemical failure will be defined using the PSA nadir plus 2ng/ml definition, as recently recommended by a consensus panel of experts (Roach et al. 2006;Thames et al. 2003). Freedom from biochemical failure is defined as the date from randomization to date of the earliest confirmed biochemical failure. Prostate cancer specific survival is defined as the date from randomization to date of death due to prostate cancer. Overall survival is defined as date from randomization to date of death due to all causes. The data will be censored at the time of last follow-up when patient is event-free. If patient refuses to continue with the protocol every attempt will be made to continue follow-up and analyze as per intent to treat. If they refuse follow-up they will be censored at the last follow-up. These endpoints will be followed but are not the primary endpoints for the study and thus were not used for sample size or power determination. The data analyses will be performed on all randomized patients. The probability distribution of all these endpoints will be estimated using Kaplan-Meier method (Mantel *et al* 1966). The comparison between treatment groups will use a Cox multivariate regression model (Cox 1972) adjusting for stratification factors.

1.4.3 Quality of Life Assessment

Patient-reported Health Related Quality of Life outcomes will be collected using the Expanded Prostate Cancer Index Composite (EPIC-26) and SF-12 Health Survey (Befort et al. 2005;Sanda et al. 2008). Data will be collected at baseline and 3, 6, 12, 18 and 24 months after completion of radiation. This is not a primary endpoint for the study and was not used for sample size determination.

Quality of life will be evaluated including all patients entered in the study. All components and single items are scored on categorical scales and will be summarized as a global health/quality of life scale at each time point. All data will be analyzed using descriptive statistics for the subscales and single items for each study group at each of the assessment points. The global health status/QoL scale will be used as an overall measure for QoL. Quality of Life between the two treatment groups will be evaluated in a longitudinal fashion using the longitudinal mixed model (PROC Mixed in SAS). The model will allow the change of QoL in the two groups over time to be investigated.

1.4.4 Exploratory and Subset Analyses

There is some evidence that radiation therapy plus ProstAtak™ without ADT may be comparable to radiation therapy plus ADT in patients with defined intermediate risk (unpublished data). There is also evidence that the overall impact of ProstAtak™ may vary depending on the patients' specific risk categorization. The study will explore such associations using regression

models and Chi-square statistics to generate scientific hypothesis for further investigation. Exploratory subset analysis by geographical region of treatment (country) in addition to the stratification factors will be evaluated using stratified log rank test. Blood and tissue will be collected from patients agreeing to have their samples archived for future immune studies. The specific studies and assays have not yet been determined.

1.5 Randomization Scheme

The study will use a block stratified randomization method for treatment assignment. The randomization and treatment assignment will be done centrally. The treating physician and clinical site personnel, the central pathologist and the patients as well as the Sponsor will all be blinded to treatment assignment.

1.6 BIBLIOGRAPHY

Cox D.R.: Regression models and life-tables. J R Statist Soc 34:187-202, 1972.

Kaplan E.L., Meier P.: Nonparametric estimation from incomplete observations. Journal of the American Statistical Association 53:447-457, 1958.

Kruskal W.H., Walis W.A.: Use of ranks in one-criterion variance analysis. J Am Stat Ass 47:583-621, 1952.

Mantel N.: Evaluation of survival data and two new rank order statistics arising in its consideration. Cancer Chemother Rep 50:163-170, 1966.

Roach M., 3rd, Hanks G., Thames H., Jr., Schellhammer P., Shipley W.U., Sokol G.H., Sandler H.: Defining biochemical failure following radiotherapy with or without hormonal therapy in men with clinically localized prostate cancer: recommendations of the RTOG-ASTRO Phoenix Consensus Conference. Int J Radiat Oncol Biol Phys 65:965-974, 2006.

Thames H., Kuban D., Levy L., Horwitz E.M., Kupelian P., Martinez A., Michalski J., Pisansky T., Sandler H., Shipley W., Zelefsky M., Zietman A.: Comparison of alternative biochemical failure definitions based on clinical outcome in 4839 prostate cancer patients treated by external beam radiotherapy between 1986 and 1995. Int J Radiat Oncol Biol Phys 57:929-943, 2003.

Appendix 1

Plan for Handling Missing and Incomplete Data

Missing or incomplete data will be handled as outlined in Section 9 of the protocol. The table below exemplifies specific scenarios with their respective dates and outcomes to be recorded.

Situation	Date of recurrence or censoring	Outcome (censored or recurrence)
Treatment discontinuation for undocumented recurrence	Date of last follow-up when patient is event-free (section 9.2.1)	Censored
Treatment discontinuation for toxicity or other reason	Date of last follow-up when patient is event-free (section 9.2.1)	Censored
New anticancer treatment started	No change in patient follow-up since intent to treat would continue to govern. Confirmation of rationale for new anti-cancer treatment would be undertaken as per section 9.2.1. The patient ITT data will be handled as per section 9.4.2 “If patient refuses to continue with the protocol every attempt will be made to continue follow-up and analyze as per intent to treat. If they refuse follow-up they will be censored at the last follow-up.”	Recurrence if/when confirmed; Censored, if not confirmed and patient refuses to continue with the protocol.<br>Censored for secondary end-points if patient refuses to continue with the protocol.
Death before first recurrence assessment	Date of death.<br>DFS includes death from any cause, section 9.2.1 “The disease-free survival (DFS) is defined as time from randomization to local failure, regional failure, distant metastases or death from any cause.”	Recurrence
Death between assessment visits or after one missed visit	Date of death (section 9.2.1).	Recurrence
Death or recurrence after an extended lost-to-follow-up time (two or more missed assessments)	Date of death (section 9.2.1).<br><br>Date of confirmatory image or biopsy (section 9.2.1).	Recurrence
Loss to follow-up without death or recurrence information	Date of last follow-up when patient is event-free (section 9.2.1)	Censored.
Recurrence between assessments visits	Date of confirmatory image or biopsy (section 9.2.1).	Recurrence
Recurrence after one missed assessment visit	Date of confirmatory image or biopsy (section 9.2.1).	Recurrence

Appendix 2

Sensitivity Analyses to be Performed in Support of Primary Analyses

In addition to the planned primary analyses, the data will be analyzed with varying assumptions to test the robustness of the results.

1- To test for potential bias in distribution of patients with one high-risk characteristic, the data will be analyzed without inclusion of these patients in either treatment or control group.

2- To test for the potential effects of bias in time to event or missing data point analyses, the data will be evaluated with the assumptions of recurrence and time to recurrence altered as per the table below.

**Situation**	**Date of recurrence or censoring**	***Sensitivity Date of recurrence or censoring***	**Outcome (censored or recurrence)**	***Sensitivity Outcome (censored or recurrence)***
New anticancer treatment started	Date of confirmatory image or biopsy (section 9.2.1). Date of last follow-up when patient is event-free (section 9.2.1)	*Date of initiation of new treatment*	Recurrence if/when confirmed; Censored, if not confirmed and patient refuses to continue with the protocol.	*1-* Recurrence<br>*2- Censored*
Death before first recurrence assessment	Date of death.	Date of death.	Recurrence	*Censored*
Death or recurrence after an extended lost-to-follow-up time (two or more missed assessments)	Date of death (section 9.2.1).<br>Date of confirmatory image or biopsy (section 9.2.1).	*1- Date of first missed visit*	Recurrence	Recurrence<br>*2- Censored at date of last visit*
Recurrence after one missed assessment visit	Date of confirmatory image or biopsy (section 9.2.1).	*Date of first missed visit*	Recurrence	Recurrence

Appendix 3

DETAILED DESCRIPTIONS OF STUDY OBJECTIVES AND ESTIMAND FRAMEWORK

Study Objectives

The overall objective of this study as described in the clinical study protocol is to determine if adjuvant therapy with CAN-2409 (aglatimagene besadenovec, also referred to as AdV-tk) plus the prodrug valacyclovir (together referred to as ProstAtak®, hereafter referred to as "CAN-2409 plus prodrug") improves the clinical outcome of radiation therapy for newly diagnosed prostate cancer.

Study Estimands

An addendum to ICH E9(R1) Statistical Principles for Clinical Trials: Addendum: Estimands and Sensitivity Analysis in Clinical Trials, May 2021) introduces the concept of an estimand which translates the trial objective into a precise definition of the treatment effect that is to be estimated. The description of the estimand includes four attributes: the population, the variable (or endpoint) to be obtained for each patient, the specification of how to account for intercurrent events (ICEs), and the population-level summary for the variable. These attributes are described below.

The primary estimand is the relative hazard of DFS following prescribed therapy with CAN-2409 plus prodrug as adjunct to radiation therapy among adult patients with histologically confirmed adenocarcinoma of the prostate with NCCN risk criteria of intermediate risk or high risk with a single high-risk factor and an ECOG performance score of 0-2 that are planning and medically able to undergo standard prostate-only external beam radiation therapy and have not received prior therapy except transurethral resection of the prostate or ADT (no more than 6 months). The variable of interest is DFS, defined as time from randomization until the earliest of local failure, regional failure, distant metastases, or death from any cause (protocol section 9.2.1). The analysis will be performed on all randomized patients in an ITT analysis population. ICEs will be handled with the following strategies and allowing interpretations listed below:

- Treatment Policy Strategy: The occurrence of the ICE is considered irrelevant, and the data will be collected and analyzed in the same manner as if the event did not occur. This strategy will be employed for the primary analysis in accordance with the ITT principle.
- Composite Strategy: The occurrence of the ICE is incorporated into the endpoint.
- While on Treatment Strategy: The treatment effect will be estimated by including all measurements taken up until the ICE occurs.
- Hypothetical Strategy: The treatment effect will be estimated in a hypothetical scenario in which the ICE did not occur.

Table 1 and Table 2 below shows the strategies of handling ICEs for primary estimand and different supplementary estimands. Robustness of the primary estimand will be assessed through different supplementary estimands using different strategies for ICEs.

The population level summary is the hazard ratio between treatment groups (CAN-2409 plus prodrug vs placebo plus prodrug) for the event of recurrence of prostate cancer or death. Estimates are obtained from a Cox proportional hazard model using the ITT population, adjusted for the 2 randomization stratification factors: planned ADT use (yes/no) and baseline disease status (intermediate/high); statistical significance will be determined from a stratified log-rank test adjusting for the two randomization stratification factors.

Table 1: Strategies of Intercurrent Events for Primary Estimand

**Intercurrent events**	**Primary estimand**
Treatment discontinuation for undocumented recurrence	Treatment-policy strategy:<br>Censored at the date of last follow-up when patient is event-free
Treatment discontinuation for toxicity or other reason	Treatment-policy strategy:<br>Censored at the date of last follow-up when patient is event-free
New anticancer therapy started	Treatment-policy strategy:<br>• Censored at the date of last follow-up when patient is event-free if no event confirmed<br>• Event at date of recurrence if event confirmed
Death before first recurrence assessment	Composite Strategy:<br>Event at the date of death
Death between assessment visits or after one missed visit	Composite Strategy:<br>Event at the date first event (Date of death, or Date of confirmatory image or biopsy)
Death or recurrence after an extended lost-to-follow-up time (two or more consecutive missed assessments)	Composite Strategy:<br>Event at the date first event (Date of death, or Date of confirmatory image or biopsy)
Loss to follow-up without death or recurrence	Treatment-policy strategy:<br>Censored at the date of last follow-up when patient is event-free
Recurrence between assessment visits	Composite Strategy:<br>Event at date of recurrence (Date of confirmatory image or biopsy)
Recurrence after one missed assessment visit	Composite Strategy:<br>Event at date of recurrence (Date of confirmatory image or biopsy)

Table 2: Strategies of Intercurrent Events for Supplementary Estimands

**Intercurrent events**	**Supplementary Estimand #1 (Censor non-prostate cancer associated death)**	**Supplementary Estimand #2 (Censor new anticancer therapy at date of last visit)**	**Supplementary Estimand #3 (Censor unknown cause death)**
Treatment discontinuation for undocumented recurrence	Same as primary	Same as primary	Same as primary
Treatment discontinuation for toxicity or other reason	Same as primary	Same as primary	Same as primary
New anticancer therapy started	Same as primary	Hypothetical strategy: Censored at the last follow-up when patient is event-free before the new anticancer therapy.	Same as primary
Death before first recurrence assessment	If death is prostate cancer associated, Composite Strategy: Event at the date of death;<br>If death is non-prostate cancer associated (other causes or unknown), Hypothetical Strategy: Censored at the date of death	Same as primary	If the death is unknown cause, Hypothetical Strategy: Censored at the date of death Otherwise, Composite Strategy: Event at the date of death
Death between assessment visits or after one missed visit	If death is prostate cancer associated, Composite Strategy: Event at the date of death;<br>If death is non-prostate cancer associated (other causes or unknown), Hypothetical Strategy: Censored at the date of death	Same as primary	If the death is unknown cause, Hypothetical Strategy: Censored at the date of death Otherwise, Composite Strategy: Event at the date of death
Death or recurrence after an extended lost-to-follow-up time (two or more consecutive missed assessments)	If death is prostate cancer associated, Composite Strategy: Event at the date of death;<br>If death is non-prostate cancer associated (other causes or unknown), Hypothetical Strategy: Censored at the date of death	Same as primary	If the death is unknown cause, Hypothetical Strategy: Censored at the date of death Otherwise, Composite Strategy: Event at the date of death

**Intercurrent events**	**Supplementary Estimand #1 (Censor non-prostate cancer associated death)**	**Supplementary Estimand #2 (Censor new anticancer therapy at date of last visit)**	**Supplementary Estimand #3 (Censor unknown cause death)**
Loss to follow-up without death or recurrence	Same as primary	Same as primary	Same as primary
Recurrence between assessment visits	Same as primary	Same as primary	Same as primary
Recurrence after one missed assessment visit	Same as primary	Same as primary	Same as primary

**Intercurrent events**	**Supplementary Estimand #4 (Appendix 2 analysis: Censor events right after consecutive missed visits)**	**Supplementary Estimand #5 (New anticancer therapy will be considered as event)**	**Supplementary Estimand #6 (Appendix 2 analysis: Censor death before first recurrence assessment)**
Treatment discontinuation for undocumented recurrence	Same as primary	Same as primary	Same as primary
Treatment discontinuation for toxicity or other reason	Same as primary	Same as primary	Same as primary
New anticancer therapy started	Same as primary	Composite Strategy: Event at the date of first event (Date of death, or Date of confirmatory image or biopsy, or Date of new anticancer therapy	Same as primary
Death before first recurrence assessment	Same as primary	Same as primary	Hypothetical Strategy: Censored at the date of death
Death between assessment visits or after one missed visit	Same as primary	Same as primary	Same as primary
Death or recurrence after an extended lost-to-follow-up time (two or more consecutive missed assessments)	Hypothetical strategy: Censored at the date of at the last follow-up when patient is event-free prior to the consecutive missed visits	Composite Strategy: Event at the date of first missed follow-up among consecutive misses	Same as primary
Loss to follow-up without death or recurrence	Same as primary	Same as primary	Same as primary
Recurrence between assessment visits	Same as primary	Same as primary	Same as primary
Recurrence after one missed assessment visit	Same as primary	Same as primary	Same as primary

**Intercurrent events**	**Supplementary Estimand #7 (Appendix 2 analysis: Consider different event date for recurrence after one missed visit)**	**Supplementary Estimand #8 (Appendix 2 analysis: Censor new anticancer therapy at date of the therapy)**
Treatment discontinuation for undocumented recurrence	Same as primary	Same as primary
Treatment discontinuation for toxicity or other reason	Same as primary	Same as primary
New anticancer therapy started	Same as primary	Hypothetical strategy: Censored at the last follow-up when patient is event-free before the new anticancer therapy.
Death before first recurrence assessment	Same as primary	Same as primary
Death between assessment visits or after one missed visit	Same as primary	Same as primary
Death or recurrence after an extended lost-to-follow-up time (two or more consecutive missed assessments)	Same as primary	Same as primary
Loss to follow-up without death or recurrence	Same as primary	Same as primary
Recurrence between assessment visits	Same as primary	Same as primary
Recurrence after one missed assessment visit	Composite Strategy:<br>Event at date of first missed follow-up before the recurrence	Same as primary

Appendix 4

GENERAL CONSIDERATIONS FOR DATA ANALYSES AND HANDLING

General Summary Table and Individual Patient Data Listing Considerations

Data summaries will be presented by the treatment arm and overall.

Tabulations will be produced for appropriate demographic, baseline, efficacy, and safety parameters. For categorical variables, summary tabulations of the number and percentage of patients within each category (with a category for missing data) of the parameter will be presented. For continuous variables, the number of patients, mean, median, standard deviation, minimum, and maximum values will be presented.

Decimal precision will be as follows:

- Mean will contain one more decimal place than actual values.
- Median will contain one more decimal place than actual values.
- Standard deviation will contain two more decimal places than the actual values.
- Minimum and maximum will have the same precision as the actual values.

For time-to-event variables, percentages of patients experiencing that event will be presented and median time to event will be estimated using Kaplan-Meier methodology. As appropriate, a 95% confidence interval (CI) will be presented.

All data listings that contain an evaluation date will contain a relative study day (Rel Day). Pre-treatment and on-treatment study days are numbered relative to the day of the first dose of study medication which is designated as Day 1.

All output will be incorporated into Microsoft Word or Excel files, or Adobe Acrobat PDF files, sorted and labeled according to ICH recommendations, and formatted to the appropriate page size(s).

Data Management

All descriptive statistical analyses will be performed using SAS statistical software Version 9.4 or later, unless otherwise noted.
Medical history and adverse events will be coded using the Medical Dictionary for Regulatory Activities (MedDRA) Version 27.0. Concomitant medications will be coded using the World Health Organization (WHO) Drug Dictionary Version September 2024. Adverse events and clinical laboratory parameters will be graded per National Cancer Institute (NCI) Common Toxicity Criteria for Adverse Events (CTCAE) Version 4.0.

Data Presentation Conventions

Clinical Data Interchange Standards Consortium (CDISC) study data tabulation model (SDTM) datasets will be provided for the study source data. To facilitate data display and interpretation, analysis datasets will be produced in the CDISC analysis data model (ADaM).

Analysis Populations

The following analysis populations will be used in the reporting of this study:

- Intent-to-Treat (ITT): The ITT population is defined as all patients randomized, regardless of whether they received any study treatment. Patients will be grouped as randomized.
- Safety: The safety population is defined as all patients who received at least one CAN-2409/placebo injection.
- Per-protocol population: population that completed all 3 courses of CAN-2409/placebo+ valacyclovir (at least 7 days) and radiation therapy +/- ADT. Patients with major treatment deviations will be excluded (e.g. those who received long term ADT, pelvic or lymph node radiation, brachytherapy).

The ITT population will be the primary population for presentation of baseline, demographics, and efficacy parameters. The safety population will be the primary population for presentation of safety parameters.

Baseline Definition

For all analyses, baseline will be defined as the most recent measurement prior to the first CAN-2409 or placebo injection. Values collected at unscheduled visits prior to the first injection will be included in the calculation of baseline values.

Subgroup Analyses

The primary and the key secondary efficacy endpoint will be assessed in the following subgroups based on baseline characteristics:

- Baseline NCCN Risk group (intermediate vs high)
- Planned ADT use (yes vs no)
- Actual ADT use (yes vs no)
- Age (<65 vs ≥65)
- Race/Ethnicity

Additional safety assessments by subgroup may also be performed.

Derived and Transformed Data

Baseline Age

Age as reported by the site will be used for baseline age. If age was not reported, but full date-of-birth is available, age will be derived as follows, rounded down to the integer:
Age (years) = (date of informed consent – date of birth + 1) / 365.25

Study Day

All data listings that contain an evaluation date will contain a relative study day (Rel Day). Pre-treatment and on-treatment study days are numbered relative to the day of the first injection of CAN-2409 or placebo, which is designated as Day 1.

Visit Windows

It is expected that all visits should occur according to the protocol schedule. All data will be tabulated per the evaluation visit as recorded in the clinical database even if the assessment is outside of the visit window. Derivation or transformation of visit windowing is not planned.
In data listings, the relative day of all dates will be presented.

Handling of Missing Data

All data recorded in the clinical database will be included in data listings that will accompany the clinical study report.

Missing Efficacy Endpoints

There will be no imputation for missing data in the primary efficacy analysis. Patients that do not experience an event will be censored per the censoring rules described in Table 1. ICEs related to missed visits will be handled as described for the supplementary estimands in Table 2.
Similarly, there will be no imputation for missing data in the analysis of secondary time-to-event endpoints; analyses will be performed per censoring rules described in Table 1.
For the secondary endpoint of PSA nadir, patients with missing post-baseline data will be assumed as "non-responders" (i.e., as not having a PSA nadir ≤0.2 ng/ml).
For the QoL EPIC-26 questionnaire, missing data will be handled as described in the questionnaire scoring manual. If ≥20% of the items that comprise a domain score or subscale score are missing, the domain or subscale score will not be calculated.

Missing Efficacy Data Due to COVID-19 Pandemic

In the first quarter of 2020 an outbreak of respiratory disease caused by a novel coronavirus (COVID-19) caused shelter-in-place orders issued by governments around the world. As a result, the conduct of clinical trials could be impacted by voluntary/involuntary clinical sites closure.
To mitigate the effect COVID-19 pandemic may have on the assessment of efficacy endpoints, missing disease assessments at scheduled visit(s) due to COVID-19 will be assessed. The primary analyses will not be modified to accommodate data missing due to COVID-19 differently than other missing data; however, additional sensitivity analyses may be performed based on the extent of protocol violations due to COVID-19.

Missing Start and Stop Dates for Prior and Concomitant Medication

Prior or concomitant medications with incomplete start dates will be handled as specified in Table 3 for the purpose of determining whether a non-study medication is a concomitant medication. Missing or partial dates for start of new anticancer therapy will be handled using the same methods.

Missing Start and Stop Dates for Adverse Events

When tabulating adverse event data, missing and partial onset dates will be handled as specified in Table 3 for the purpose of determining whether an event is treatment emergent (including an acute or late toxicity). If the onset date is missing entirely and reporting date is

available, reporting dates will be used as the onset date before applying imputation rules in the table.

Table 3: Missing Data Imputation for Concomitant Medications and Adverse Events

Missing Information	Date Imputation
Onset day of month is missing, not same month and year as first study treatment	Date = First day of month
Onset day of month is missing, is same month and year as first study treatment	Date = First day of treatment
Onset day and month are missing, not same year as first study treatment	Date = 01 January
Onset day and month are missing, same year as first study treatment	Date = First day of study treatment
Missing onset date in entirety	Date = First day of treatment
End day is missing	Date = Last day of month
End of day and month is missing	Date = 31 December
Missing end date	Considered ongoing
Imputation resulted an end date after the death date	Stop date = death date
Imputation resulted in an end date before a start date	Start date = stop date

STUDY POPULATION

Patient Disposition

Patient disposition will be tabulated by treatment and overall, and include the number screened, the number randomized, the number treated (i.e., received at least one injection), the number in each patient population for analysis, and the number who withdrew from treatment or study prematurely, including primary reason for withdrawal. Survival status at end of study will be presented.
A by-patient data listing of study completion information, including the reason for premature treatment or study withdrawal as applicable, will be presented.

Screen Failures

The disposition summary will include the number of screen failures for the study. Data for screen failure patients will be included in by-patient data listings for disposition and inclusion and exclusion criteria not met. Screen failure patients will be excluded from all other by-patient data listings.

Listing of Patient Inclusion and Exclusion Criteria

A by-patient data listing of all eligibility criteria will be presented, noting any inclusion and exclusion criteria not met. Screen failure patients will be included in this data listing.

Protocol Deviations

The Sponsor or designee will be responsible for producing the final protocol deviations file (formatted as a Microsoft Excel file); this file will include a description of the protocol deviations and clearly identify whether or not they warrant exclusion from sensitivity analyses of efficacy results. This file will be finalized prior to hard database lock.
All protocol deviations will be presented in a data listing and included in the CSR following ICH E3 recommendations. The data listing will include a flag indicating whether the protocol deviation was a result of the COVID-19 pandemic.

Demographic and Baseline Characteristics
Demographics and baseline characteristics will be summarized by treatment group for the ITT population using descriptive statistics. No formal statistical comparisons will be performed. Demographic data will include age, race, and ethnicity. Baseline characteristics include age, and weight.
Demographic and baseline data for each patient will be provided in by-patient data listings.

Baseline Disease Characteristics and History
Baseline disease history will be summarized by treatment arm based on the ITT population using descriptive statistics and provided in by-patient data listings.
Baseline disease characteristics will be summarized and presented by treatment group and overall, including ECOG performance status, PSA, Gleason score, clinical tumor stage, EPIC-26, and planned ADT use (yes or no).

Medical History and Medical Conditions Present at Entry
Medical history will be summarized by treatment arm based on the ITT population using descriptive statistics and provided in by-patient data listings.
Medical history will be coded and summarized by system organ class (SOC) and preferred term (PT) using the number and percentage of patients who had at least one occurrence of an SOC or PT. The summary will be sorted alphabetically in SOC and by decreasing frequency of PT in the Test Arm within an SOC.

Prior Medications and Medications Present at Entry
Concomitant medications will be coded using the WHO Drug Dictionary. Results will be tabulated by anatomical therapeutic chemical (ATC) class Level 3 and preferred term.
Prior medications will be tabulated by treatment group and include medications with a start and stop date prior to first dose and medications with a start date prior to first dose that did not end prior to first dose (i.e., were either ongoing at time of first dose or with a missing end date).

EFFICACY

Statement of the Null and Alternate Hypotheses
The primary analysis of DFS will occur after at least 98 events (disease recurrence [local failure, regional failure, or distant metastases] or death are observed. DFS will be compared between

treatment arms for superiority of CAN-2409 versus placebo, in combination with valacyclovir and standard of care radiation therapy, and at a two-sided type I error rate of 0.05.
Null Hypothesis: The null hypothesis is that there is no difference in DFS between patients treated with CAN-2409 plus valacyclovir (Test Arm) versus placebo plus valacyclovir (Control Arm).

Alternative Hypothesis: The alternative hypothesis is that the DFS in the CAN-2409 plus valacyclovir treatment group (Test Arm) differs from that of the placebo plus valacyclovir treatment group (Control Arm).

Multiple Comparisons and Multiplicity
A statistical test will be deemed significant if the corresponding p-value is less than or equal to 0.05, unless otherwise specified. All p-values will be 2-sided. A closed-testing hierarchical approach will be used for type I error control in testing of multiple endpoints. If the primary efficacy endpoint is found to be statistically significant at the 0.05 level, secondary endpoints will also be assessed for superiority in the following order:

1. PSA nadir
2. Freedom from biochemical failure
3. Prostate cancer specific survival
4. Overall survival
5. QoL assessment

Analysis of the Primary Efficacy Endpoint

Primary Efficacy Analysis

Analyses of DFS will be based on the ITT population. Patients that do not experience an event, including patients that are lost to follow-up, will be censored at date of last recurrence follow-up visit (i.e., PSA and DRE assessment visits). Further details on the event/censoring method are described in Table 1 for primary estimand.
The primary analysis of DFS will be estimated using the Kaplan-Meier technique (Kaplan 1958). The primary analysis to compare the differences between the treatment arms for this endpoint will use a stratified log-rank test adjusting for the two randomization stratification factors (ADT use and NCCN baseline disease risk category) and a two-sided significance level of 0.05.
The number and percentage of patients with each type of event (biopsy proven recurrence of prostate cancer, treatment failures, death) and number and percentage of patients censored with primary reason for censoring will be reported. Median DFS and probability of DFS from 6 months to the end of the follow-up period will be reported at 6-month intervals. The two-sided 95% CI for the median and 6-month intervals will be calculated using the complementary log-log transformation method. The hazard ratio (Test Arm/Control Arm) and the corresponding 2-sided 95% CI, as estimated from the Cox proportional hazard model (Cox 1972) adjusting for the two randomization stratification factors (ADT use and NCCN baseline disease risk category), will be presented.

Sensitivity Analyses of the Primary Efficacy Results
Supplementary estimands of using different strategies for ICEs described in Table 2 will be used to show the robustness of the primary estimand. In addition, following sensitivity analyses will also be used to show the robustness of the primary estimand.

- Log-rank test and Cox proportional hazard model without adjusting stratification factors

- Log-rank test and Cox proportional hazard model adjusting actual stratification factors
- Log-rank test and Cox proportional hazard model adjusting stratification factors on per-protocol population.

Analysis of the Secondary Efficacy Endpoints

PSA Nadir

The proportion of patients with PSA nadir ≤0.2 ng/ml in the CAN-2409 plus prodrug arm will be compared to the control arm using a logistic regression model to estimate the odds of PSA nadir ≤0.2 ng/ml between the two arms, adjusting for the randomization stratification factors as covariates. Patients without a post-baseline PSA assessment will be assumed as "non-responders" (i.e., as not having a PSA nadir ≤0.2 ng/ml).

Freedom from Biochemical Failure, Prostate Cancer Specific Survival, and Overall Survival

Biochemical failure is defined as a PSA value greater than or equal to the PSA nadir plus 2ng/ml, as recommended by a consensus panel of experts (Roach 2006; Thames 2003).
The freedom from biochemical failure endpoint is defined as the date from randomization to date of the earliest confirmed biochemical failure. Patients that do not experience biochemical failure (including patients that are lost to follow-up) will be censored at date of last PSA assessment when they were event-free.
Prostate cancer specific survival is defined as the date from randomization to date of death due to prostate cancer, as confirmed through adjudication on cause of death. Deaths due to causes other than prostate cancer will be censored at the date of death. Overall survival is defined as date from randomization to date of death due to any cause. Patients that are alive at time of the analysis (including patients that are lost to follow-up) will be censored at date of last contact.
These time-to-event endpoints will be analyzed using methodology similar to the primary endpoint using the ITT population: the hazard ratio (Test Arm/Control Arm) and the corresponding 2-sided 95% CI will be estimated from a Cox proportional hazard model adjusting for the two randomization stratification factors, and statistical significance of the comparison between treatment groups will be determined from a stratified log-rank test.

Quality of Life Assessment

Patient-reported Health Related QoL outcomes will be collected using the EPIC-26 questionnaire, assessed at baseline and 3, 6, 12, 18 and 24 months after completion of radiation.

The questionnaire contains 26 items and 5 domains: Urinary Incontinence, Urinary Irritative/Obstructive, Bowel, Sexual, and Hormonal. All response items are scored on a Likert scale, and multi-item scale domain scores will be transformed linearly to a 0-100 scale per the EPIC-26 scoring manual. Individual item and domain scores will be summarized at each time point using the ITT population.

Assessment of the EPIC-26 questionnaire over time between the two treatment groups will be evaluated by domain in a longitudinal fashion using a longitudinal mixed model (PROC Mixed in SAS). The model will allow the change of QoL in the two groups over time to be investigated.

SAFETY AND TOLERABILITY

Overall Summary of Tolerability

The overall toxicity profile analyses will be based on the safety population, including all randomized patients who started study treatment and presented by treatment, according to the treatment actually received, and overall.

Adverse Event Preferred Term and Body/Organ System Summary Tables

All adverse events (AEs) will be coded using the MedDRA coding system Version 27.0 and displayed in tables and data listings using SOC and PT. AEs are summarized by patient incidence rates; therefore, in any tabulation, a patient contributes only once to the count for a given adverse event.

Analyses of AEs will be performed for those events that are considered treatment-emergent, where treatment-emergent is defined as any AE with onset after the administration of study medication (i.e., the first CAN-2409 or placebo injection) until 30 days after the last administration of study treatment , or any event that was present at baseline but worsened in intensity through 30 days after the last administration of study treatment. AEs will be further categorized into acute or long-term toxicity events.

- Acute toxicity TEAEs include all events with a start date on or after the first study medication injection until 30 days after the last administration of study treatment.
- Late toxicity AEs include all events with a start date on or after the end of the acute toxicity monitoring period up to 5 years post radiation completion.

Summaries of Adverse Event Incidence Rates for All Patients

A summary of AEs will be presented overall and by reporting period (i.e., acute, late) and include the number and percentage of patients with at least one of the following:

- TEAEs
- TEAEs assessed by the Investigator as related to treatment (definitely, likely, or possibly related)
- SAEs
- SAEs assessed by the Investigator as related to treatment
- Severe AEs (≥ Grade 3)
- AEs leading to withdrawal of treatment
- AEs leading to death

In addition to the overall summary, separate summaries of the above AE categories will be presented by SOC and PT. In the summary of severe AEs, events will be presented overall and by maximum grade.
All AEs occurring on-study will be listed in patient data listings. By-patient listings also will be provided for SAEs, AEs leading to withdrawal of treatment, and AEs leading to death.

Missing and Partial AE Onset Dates

Handling of missing and partial AE onset dates will be performed as described in Table 3.

Study Drug Exposure

Study exposure will be summarized for each treatment component while on study, presented by treatment group using descriptive statistics.

The following parameters will be presented:

- The number of patients that completed each course of treatment (i.e., each injection followed by oral valacyclovir)
- The number and percentage of patients that received radiation, overall and by radiation type (standard fractionated radiation or moderate hypofractionated radiation)
- Total radiation dose received (Gy)
- The number of patients that received ADT, including LHRH (GnRH) agonist or antagonist, and/or anti-androgen therapy
- The duration of ADT therapy

All exposure data, including all modifications and interruptions to treatment will be presented in by-patient data listings.

Concomitant and Other Medications

Concomitant medications will be coded using the WHO Drug Dictionary.
Concomitant medications will be tabulated by treatment group and include medications with a start date on or after first dose and medications with a start date prior to first dose that did not end prior to first dose (i.e., were either ongoing at time of first dose or with a missing end date). The use of concomitant medications will be included in a by-patient data listing.

Routine Laboratory Data

Clinical laboratory values will be expressed in the International System of units.
If data permits, the actual value and change from baseline to each scheduled on-study evaluation will be summarized for each clinical laboratory parameter, including hematology and clinical chemistry. In the event of repeat values, the last non-missing value per study day/time will be used.
All laboratory data will be provided in data listings. A subset listing will be presented for all abnormal laboratory values.

Revision History

Version #	Version Date	Description and Reason of Modification
1.0	30JUL2010	Initial draft
2.0	26SEP2011	Revised SAP: 1. Clarification added on page 2 section 1.1 "compared to standard of care alone (Control Arm)" changed to "compared to placebo + valacyclovir + standard of care (Control Arm);" 2. Correction made in Appendix 2 item 1, the word "on" changed to "one" so that the phrase now reads "one high-risk characteristic."
3.0	01OCT2024	Revised SAP: The SAP was amended to update content to accurately reflect current product nomenclature, sponsor name and version number, and to update data handling conventions in alignment with current Guidance for Industry: E9(R1) Statistical Principles for Clinical Trials: Addendum: Estimands and Sensitivity Analysis in Clinical Trials (May 2021). Other editorial updates reflect general considerations for data analyses and data handling conventions, in alignment with current best practices.
